# Unveiling the Untapped Potential of Bertagnini’s Salts in Microwave-Assisted Synthesis of Quinazolinones

**DOI:** 10.3390/molecules29091986

**Published:** 2024-04-26

**Authors:** Shyamal Kanti Bera, Sourav Behera, Lidia De Luca, Francesco Basoccu, Rita Mocci, Andrea Porcheddu

**Affiliations:** 1 Dipartimento di Scienze Chimiche e Geologiche, Università degli Studi di Cagliari, Cittadella Universitaria, 09042 Cagliari, Italy; shymal.bera@niser.ac.in (S.K.B.); sourav.behera@unica.it (S.B.); francesco.basoccu@unica.it (F.B.); rita.mocci@unica.it (R.M.); 2 Dipartimento di Scienze Chimiche, Fisiche, Matematiche e Naturali, Università degli Studi di Sassari, Via Vienna 2, 07100 Sassari, Italy; ldeluca@uniss.it

**Keywords:** microwave irradiation, Bertagnini’s salts, green solvent, quinazolinones

## Abstract

Microwave-assisted organic synthesis (MAOS) has emerged as a transformative technique in organic chemistry, significantly enhancing the speed, efficiency, and selectivity of chemical reactions. In our research, we have employed microwave irradiation to expedite the synthesis of quinazolinones, using water as an eco-friendly solvent and thereby adhering to the principles of green chemistry. Notably, the purification of the product was achieved without the need for column chromatography, thus streamlining the process. A key innovation in our approach is using aldehyde bisulfite adducts (Bertagnini’s salts) as solid surrogates of aldehydes. Bertagnini’s salts offer several advantages over free aldehydes, including enhanced stability, easier purification, and improved reactivity. Green metrics and Eco-Scale score calculations confirmed the sustainability of this approach, indicating a reduction in waste generation and enhanced sustainability outcomes. This methodology facilitates the synthesis of a diverse array of compounds, offering substantial contributions to the field, with potential for widespread applications in pharmaceutical research and beyond.

## 1. Introduction

**Figure 1:**
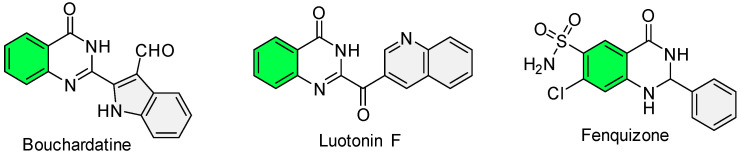
Examples of bioactive quinazolinone scaffolds.

The construction of *N*-heterocyclic scaffolds [1,2,3,4] is pivotal in pharmaceutical research and drug development due to their diverse pharmacological effects and versatile structures. In this framework, quinazolinone derivatives exhibit various biological effects [5], including antihypertensive [6], antibacterial [7,8], anticancer [9,10], and anti-inflammatory properties [11]. Additionally, these scaffolds are valuable tools in biochemical and biological studies [12,13], helping to elucidate disease mechanisms and identify potential drug targets [14]. This diverse range of properties positions them as promising candidates for therapeutic intervention, as illustrated in Figure 1. Bouchardatine exhibits anti-proliferative effects on various colorectal cancer cell lines (CRCs) [15]. In contrast, Luotonin has antifungal properties [16], and Fenquizone is used as an antidiuretic to treat hypertension [17].

The synthesis of quinazolinone scaffolds [18] plays a vital role in designing and refining new drug molecules. Developing efficient methodologies for synthesizing quinazolinone derivatives enables the creation of structurally diverse analogs and facilitates structure–activity relationship (SAR) studies, which are crucial for enhancing drug efficacy and selectivity.

Numerous synthetic procedures for quinazolinone synthesis rely on metal catalysis [19,20], stoichiometric oxidants [21,22], or visible light photocatalysis [23,24], highlighting the extensive exploration and innovative advancements within the field. These methodologies often involve using costly reagents and hazardous solvents, resulting in cumbersome purification processes.

A mild and environmentally sustainable approach is necessary to overcome traditional challenges in quinazolinone synthesis. Utilizing green solvents is crucial for ensuring environmental responsibility while maintaining an enhanced level of efficiency. As the demand for sustainability grows in the chemical industry, green chemistry has emerged as a pivotal framework for chemists. It steers the design of processes and products towards minimizing or eradicating the use and generation of hazardous substances, aligning with the broader goal of promoting sustainable human development [25]. Consequently, the move towards using green solvents in chemical synthesis has become critically important, reflecting a necessary response to growing environmental concerns [26]. Water has become a leading choice in pursuing sustainability thanks to its abundant availability and eco-friendly properties [27,28]. When used as a solvent, water offers significant advantages in organic chemistry [29]. It enhances levels of reactivity and selectivity, streamlines workup procedures, facilitates catalyst recycling, and enables unique kinds of reactivity and selectivity under mild conditions [30]. Employing water as a sustainable solvent, combined with microwave-assisted methodologies for synthesizing quinazolinone scaffolds, epitomizes the principles of green chemistry. This strategy provides an environmentally mindful approach to their production.

In the constantly evolving field of organic synthesis, microwave irradiation has emerged as a catalytic force driving innovation and efficiency [31,32,33,34,35]. Leveraging the capabilities of electromagnetic waves within the microwave spectrum, chemists have discovered a potent methodology to expedite chemical reactions, thereby broadening the horizons of synthetic opportunities [36,37]. This technology’s significance lies in its ability to accelerate reaction times and improve yields, enhance levels of selectivity, and synthesize complex molecules with unprecedented ease [38,39]. Adopting microwave-assisted techniques for synthesizing nitrogen- and oxygen-containing frameworks has become a cornerstone strategy in organic chemistry [40,41,42,43,44,45], offering unparalleled advantages in reaction efficiency [46] and sustainability [47]. Microwave irradiation facilitates the rapid and uniform heating of reaction mixtures, leading to accelerated reaction rates and higher yields than conventional heating methods [48]. Within this technological framework, the application of the microwave-assisted synthesis of quinazolinone derivatives represents a leap forward in green chemistry and pharmaceutical development. This technique significantly diminishes the consumption of energy and resources, providing a more efficient pathway to synthesize these compounds with a reduced incidence of undesirable side reactions. By enabling rapid and controlled heating, microwave irradiation ensures that reaction conditions are optimal, thus leading to higher yields, greater levels of purity, and a reduction in the synthesis time compared to traditional methods. The streamlined production process facilitated by microwave-assisted synthesis enhances the efficiency of chemical reactions and broadens the scope of accessible chemical libraries. This expansion is particularly valuable in drug discovery efforts, in which the diversity and quality of chemical libraries can dramatically influence the identification and optimization of lead compounds.

Substitutions at the 2-position of quinazoline-4 (3H)-ones are crucial as they significantly influence the pharmacological effectiveness of the resulting compounds [49]. This substituent originates from the aldehyde group involved in ring closure. However, the use of aldehydes often leads to the formation of side products, especially under elevated temperatures and in aqueous solvents. Bertagnini’s salts [50,51], historically employed for purifying aldehydes [52], can now serve as substitutes for this moiety to circumvent potential side reactions, such as redox or (self-)condensation processes. In addition to their technical benefits [53], adopting aldehyde bisulfite adducts supports safer laboratory practices by eliminating the need to handle volatile and potentially hazardous free aldehydes. This approach reduces the risk of exposure to harmful volatile organic compounds (VOCs) and minimizes the potential for accidents associated with these reactive compounds. By incorporating aldehyde bisulfite adducts into synthetic procedures, researchers can create a safer working environment, enhancing the overall well-being and safety of laboratory personnel. This safer alternative highlights the importance of health and safety considerations when selecting reagents and procedures in chemical research and development.

Additionally, Bertagnini’s salts are distinguished by their substantial dipole moment, enabling highly efficient interactions with microwave radiation. This feature is particularly advantageous in MAOS, in which their ability to absorb microwave energy can lead to more rapid and energy-efficient reactions. The efficiency of Bertagnini’s salts in these processes enhances the reaction rates and contributes to a more streamlined and environmentally friendly purification process. By reducing the need for traditional, often energy-intensive purification steps, the use of Bertagnini’s salts aligns with the principles of green chemistry, emphasizing waste reduction and energy-efficient methodologies [54,55].

**Figure 2:**
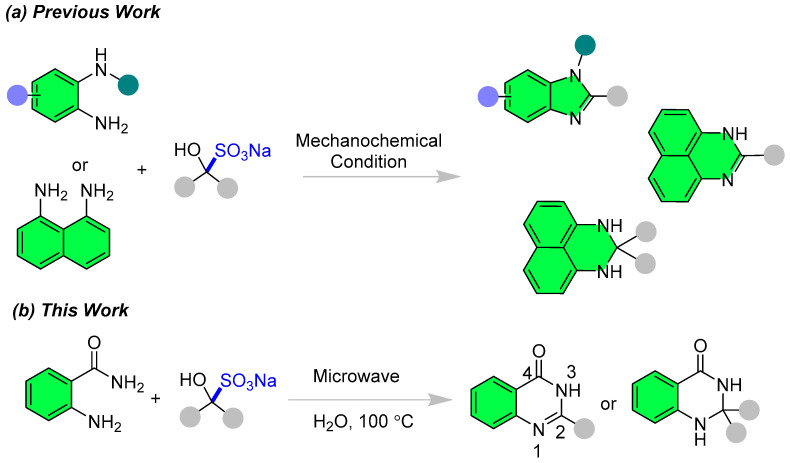
Potentiality of Bertagnini’s salts. (**a**) Mechanochemical synthesis of aza-heterocycles. (**b**) Our work is based on the microwave-induced synthesis of quinazolinones. Different colour shows different substitutions.

Our group recently investigated aza-heterocycle synthesis under mechanochemical conditions, employing Bertagnini’s salts as the sole reagent (Figure 2a) [56]. Our ongoing research on bisulfite adducts successfully demonstrated the microwave-assisted synthesis of quinazolinone scaffolds starting from anthranilamide and Bertagnini’s salts, with water as a sustainable solvent. This method simplifies the purification process and leads to an efficient yield of the desired product, as depicted in Figure 2b. This approach facilitates a cleaner synthesis process and underscores our commitment to developing sustainable and eco-friendly chemical methodologies.

## 2. Results and Discussion

### 2.1. Optimization

To optimize the reaction conditions, we selected anthranilamide (**1a**) and sodium hydroxy(phenyl)methanesulfonate (**2a**) as the standard substrates, as shown in Table 1. The preliminary experiment, which was carried out in water for 8 h, resulted in the synthesis of the products **3aa** and **3aa′**, demonstrating the potential of these conditions for effective compound formation. An NMR analysis revealed that the products (**3aa**:**3aa′**) were synthesized in a ratio of 15:85. Elevating the reaction temperature led to a marked enhancement in the conversion of **3aa**, as evidenced in entries 2, 3, and 4 (Table 1). Notably, at a temperature of 100 °C for 10 h (entry 5, Table 1), the conversion of **3aa** was fully achieved, resulting in an isolated yield of 91%. In contrast, the reaction at ambient temperature (25 °C) did not yield the anticipated product. Without a solvent, the products **3aa** and **3aa′** were produced in a ratio of 80:12 (entry 7, Table 1).

Impressively, employing DMSO as the solvent was as effective as using water, resulting in the exclusive formation of the targeted product **3aa** with an isolated yield of 86% (entry 8, Table 1). However, employing toluene as a solvent did not favor the formation of the desired product (**3aa**), highlighting the influence of solvent choice and reaction conditions on the outcome of the synthesis (entry 9, Table 1). Finally, the optimized reaction conditions were achieved by conducting the reaction with 0.5 mmol of anthranilamide (**1a**) and 0.5 mmol of sodium hydroxy(phenyl)methanesulfonate (**2a**) in 2 mL of water at 100 °C in a microwave reactor for 10 h.

When conducting the reaction in water, we noted that the final product precipitated from the aqueous medium upon completion. This phenomenon enables the straightforward recovery of the quinazolinone product **3aa** through simple filtration, obviating the need for chromatographic purification or acid–base extraction procedures. Consequently, this approach significantly diminishes the generation of additional waste, adhering to the principles of green chemistry by promoting a more sustainable and environmentally friendly synthetic process. This method not only streamlines the synthesis of quinazolinones but also highlights the potential of water as a solvent to enhance the efficiency and eco-friendliness of chemical reactions. Furthermore, the analysis of its green metrics and EcoScale score indicates that our process yields lower levels of waste and demonstrates a higher level of sustainability than those of reported methodologies (see the Supplementary Materials File for more details).

### 2.2. Synthesis of Quinazolinones

**Scheme 1:**
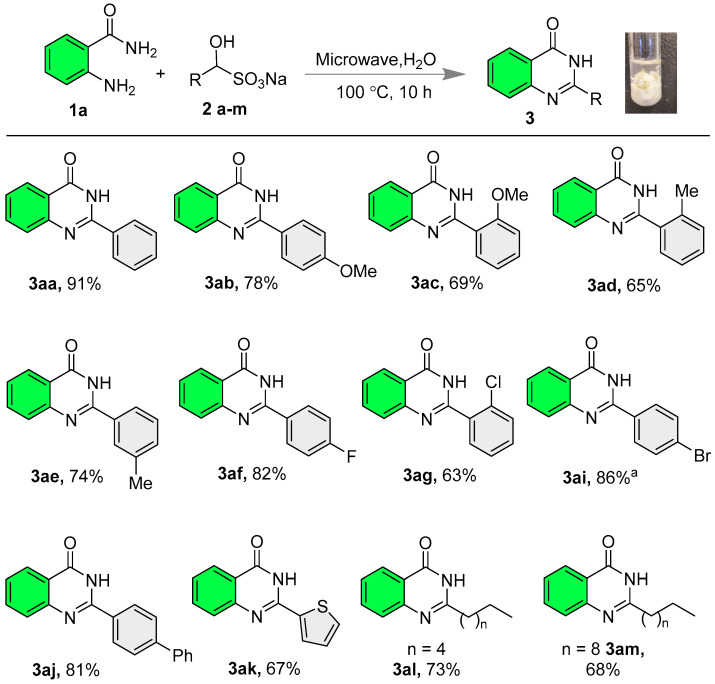
Synthesis of quinazolin-4(3*H*)-ones using unsubstituted anthranilamides. Unless otherwise stated, all the reactions were performed in water as a solvent. ^a^ The reaction was performed in DMSO.

Having refined and simplified our reaction protocol, we broadened its application to include the synthesis of various quinazolinone derivatives. This involved using unsubstituted anthranilamide **1a** alongside various substituted aldehyde bisulfite adducts, as described in Scheme 1. This expansion showcases our approach’s versatility and effectiveness and opens new avenues for synthesizing a broad spectrum of quinazolinone derivatives.

Introducing a methoxy group to the bisulfite adducts enabled the efficient synthesis of **3ab** and **3ac**, achieving 78% and 69% yields, respectively. Bisulfite adducts featuring methyl groups also showed good levels of compatibility, yielding **3ad** and **3ae** with 65% and 74% yields, respectively. Halogen-substituted bisulfite adducts showed an excellent level of reactivity, leading to the production of **3af** through **3ai** with satisfactory yields. Remarkably, using bisulfite adducts with a biphenyl group facilitated the synthesis of **3aj**, resulting in an 81% yield. Incorporating thiophene into bisulfite adducts also resulted in the efficient production of **3ak** with a 67% yield. Furthermore, using bisulfite adducts of aliphatic aldehyde resulted in high yields of **3al** and **3am**.

**Scheme 2:**
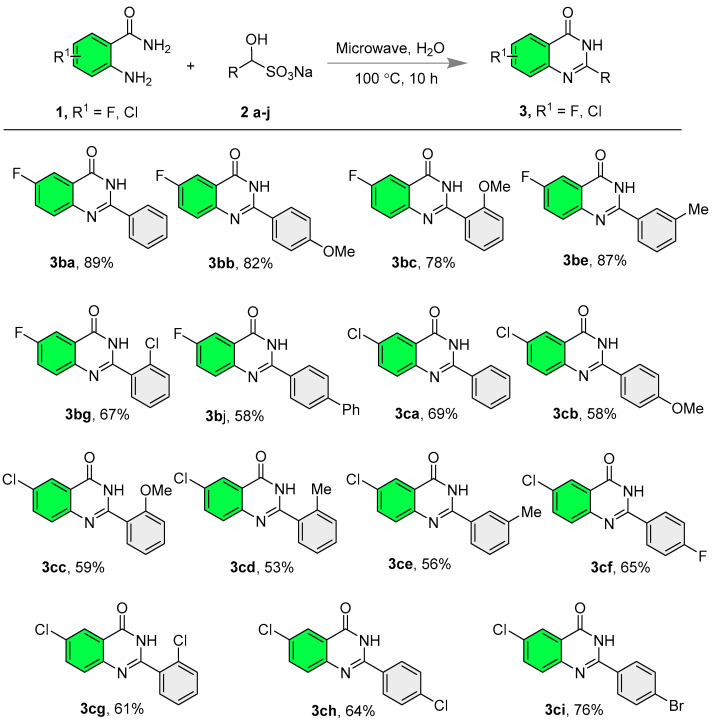
Synthesis of quinazolin-4(3*H*)-ones using fluoro- and chloro-substituted anthranilamides.

To evaluate the feasibility of the methodology, we expanded it to fluoro- and chloro-substituted anthranilamides, reacting them in the presence of various substituted aldehyde bisulfite adducts, as shown in Scheme 2. To begin with, we tested the substitution effect on the aldehyde bisulfite adducts using 2-amino-5-fluorobenzamide (**1b**) under standard reaction conditions. We found that sodium hydroxy(phenyl)methanesulfonate (**2a**) reacted with 2-amino-5-fluorobenzamide (**1b**) and produced the expected product (**3ba**) with a yield of 89%. Moreover, when methoxy and methyl groups were incorporated into the aldehyde bisulfite adducts, they effectively facilitated the conversion to the desired product (**3bb**–**3be**) with a satisfactory yield. The chloro-substituted aldehyde bisulfite adduct was efficiently transformed into the desired product **3bg**, with a yield of 67%. Again, sodium [1,1′-biphenyl]-4-yl(hydroxy)methanesulfonate (**2j**) resulted in the corresponding product (**3bj**) with a 58% yield. Further, we utilized chloro-substituted anthranilamides as coupling partners alongside a range of substituted aldehyde bisulfite adducts, yielding products (**3ca**–**3ci**) with satisfactory yields. Under standard reaction conditions, the reaction between 2-amino-5-chlorobenzamide (**1c**) and sodium hydroxy(phenyl)methanesulfonate (**2a**) yielded the desired product (**3ca**) with a 69% yield. Further, the methoxy and methyl groups containing bisulfite adducts were smoothly converted to the corresponding products (**3cb**–**3ce**) with a good yield. Also, halogen- (such as fluoro- and chloro-) substituted bisulfite adducts afforded the products (**3cf**–**3ci**) under the optimized reaction conditions. In this context, the synthesized compounds **3be**, **3bg**, **3bj**, and **3ce** have not been previously reported in the literature.

**Scheme 3:**
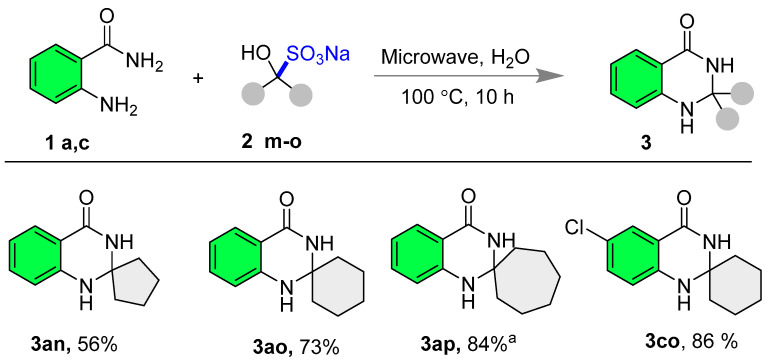
Synthesis of 2,3-dihydroquinazolin-4(1*H*)-ones using bisulfite adducts of ketones. Unless otherwise stated, all the reactions were performed in water as a solvent. ^a^ The reaction was performed in DMSO.

Furthermore, we investigated the reactivity of bisulfite adducts derived from cyclic ketones under the optimized conditions outlined in Scheme 3. Combining these bisulfite adducts with anthranilamide results in the formation of dihydroquinazolinones, a preferred structural motif in drug development [57]. Anthranilamide effectively underwent reactions with bisulfite adducts of five-, six-, and seven-membered ketones, yielding cyclized dihydro products **3an**–**3ap** with satisfactory yields. Furthermore, the 2-amino-5-chlorobenzamide smoothly reacted with bisulfite adducts of a six-membered ketone, resulting in the cyclized product **3co** with an 86% yield.

**Scheme 4:**
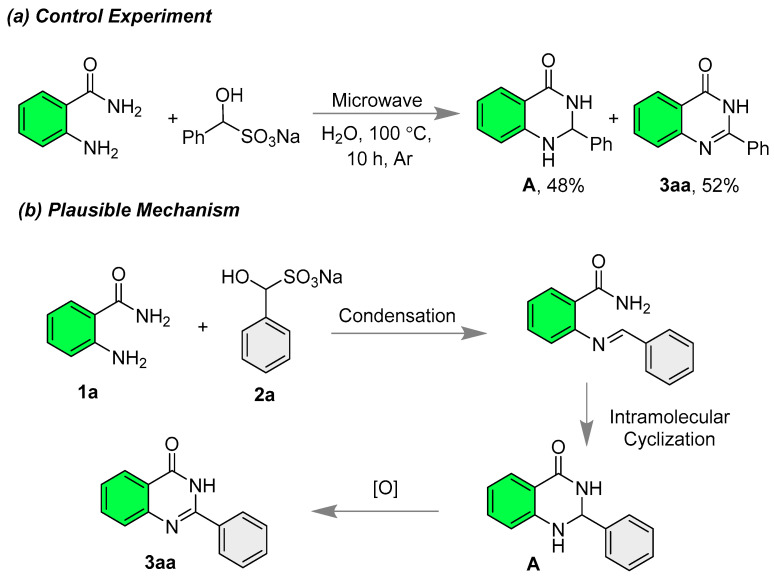
(**a**) Control experiment and (**b**) plausible mechanism.

Drawing upon the findings presented in the literature [58], within a control experiment executed under an argon atmosphere [59], it becomes clear that oxygen plays a crucial role in the reaction mechanism that leads to the formation of the quinazolinone product. The 48% yield of the dihydroproduct (**A**) under argon conditions confirms its role as an intermediate within the reaction sequence, as illustrated in Scheme 4a.

Scheme 4 illustrates the proposed reaction mechanism, in which Bertagnini’s salts initially react with anthranilamide to form an imine intermediate. This is followed by intramolecular cyclization and aerobic oxidation, culminating in the formation of the 2-phenyl quinazolin(3*H*)-one product (**3aa**). The transformation of 2-phenyl-2,3-dihydroquinazolin-4(1*H*)-one into the 2-phenylquinazolin-4(3*H*)-one product (**3aa**) mirrors the auto-oxidation processes previously observed in benzaldehyde and benzyl alcohol [60], highlighting a critical aspect of the reaction’s oxygen-dependent pathway.

## 3. Materials and Methods

### 3.1. Materials

Commercially available reagents were purchased from Acros, Aldrich, Strem Chemicals, Alfa-Aesar, and TCI Europe and were used as received. All reactions were monitored by thin-layer chromatography (TLC) performed on glass-backed silica gel plates (60 F254, 0.2 mm) (Merck), and compounds were visualized under UV light (254 nm). All reactions were performed using microwave instrument (Model: DISCOVER SP; SERIAL NO: DC8609; MODEL NO: 909155). The eluents were technical grade. ^1^H and ^13^C NMR spectra were recorded on a Varian 600 MHz and Bruker Avance III HD 600 MHz NMR spectrometer and were calibrated using trimethylsilane (TMS). Proton chemical shifts are expressed in parts per million (ppm, δ scale) and are referred to as the residual hydrogen in the solvent (CHCl_3_, 7.260 ppm or DMSO, 2.50 ppm). Data are represented as follows: chemical shift, multiplicity (s = singlet, d = doublet, t = triplet, q = quartet, m = multiplet and/or multiple resonances, bs = broad singlet, and combination of thereof), coupling constant (*J*) in Hertz (Hz), and integration. Carbon chemical shifts are expressed in parts per million (ppm, δ scale) and refer to the carbon resonances of the NMR solvent (CDCl_3_, δ 77.16 ppm or δ DMSO-d_6_, δ 39.52 ppm). Deuterated NMR solvents were obtained from Aldrich. Infrared (IR) spectra were recorded using the Jasco FTIR-4X (MODEL: PKS-D1) instrument, and data are reported in wavenumber (cm^−1^). Positive ESI-MS spectra were recorded on a high-resolution LTQ Orbitrap Elite™ mass spectrometer (Thermo Fisher Scientific, Waltham, MA, USA). The solutions were infused into the ESI source at a 5.00 μL/min flow rate. Spectra were recorded with a resolution of 120,000 (FWHM). Instrument conditions were as follows: spray voltage of 3500 V, capillary temperature of 275 °C, 12 (arbitrary units) sheath gas, 3 (arbitrary units) auxiliary gas, 0 (arbitrary units) sweep gas, and probe heater temperature of 50 °C. Yields refer to pure isolated materials after filtration only (no column chromatography).

### 3.2. Procedure A. General Procedure for the Synthesis of 2-Phenylquinazolin-4(3H)-One

Anthranilamide (0.5 mmol) and sodium hydroxy(phenyl)methanesulfonate (**2a**) (0.5 mmol) were placed in a 10 mL microwave vial equipped with a stir bar. After adding 2 mL of water, the vial was correctly capped, and then the reaction mixture underwent microwave irradiation at 100 °C and was stirred for 10 h. The resulting solid crude product was filtered and washed with water to yield 2-phenyl quinazoline-4 (3H)-one (**3aa**). In certain instances, the crude reaction mixture was quenched by pouring it into ice water to enhance product yield.

### 3.3. Characterization Data of the Synthesized Compounds

*2-Phenylquinazolin-4*(*3H*)*-one* (**3aa**) [21]: The title compound was synthesized according to general procedure A; White solid; Yield: 91% (101 mg); ^1^H NMR (600 MHz, DMSO-d_6_) δ 12.53 (s, 1H), 8.20–8.13 (m, 3H), 7.82 (t, *J* = 7.2 Hz, 1H), 7.74 (d, *J* = 8.4 Hz, 1H), and 7.60–7.48 (m, 4H); ^13^C NMR (151 MHz, DMSO-d_6_) δ 162.3, 152.3, 148.8, 134.6, 132.7, 131.4, 128.6, 127.8, 127.5, 126.6, 125.9, and 121.0.
*2-*(*4-Methoxyphenyl*)*quinazolin-4*(*3H*)*-one* (**3ab**) **[61]**: The title compound was synthesized according to general procedure A; White solid; Yield: 78% (98.5 mg); ^1^H NMR (600 MHz, DMSO-d_6_) δ 12.39 (s, 1H), 8.19 (d, *J* = 5.4 Hz, 2H), 8.13 (s, 1H), 7.80 (s, 1H), 7.70 (s, 1H), 7.47 (s, 1H), 7.08 (s, 2H), and 3.84 (s, 3H); ^13^C NMR (151 MHz, DMSO-d_6_) δ 162.3, 161.9, 151.8, 148.9, 134.5, 129.4, 127.3, 126.1, 125.8, 124.8, 120.7, 113.9, and 55.4.
*2-*(*2-Methoxyphenyl*)*quinazolin-4*(*3H*)*-one* (**3ac**) [62]: The title compound was synthesized according to general procedure A; White solid; Yield: 69% (87 mg); ^1^H NMR (600 MHz, DMSO-d_6_) δ 12.08 (s, 1H), 8.15 (dd, *J* = 7.8, 1.2 Hz, 1H), 7.85–7.79 (m, 1H), 7.71 (dd, *J* = 12.0, 4.8 Hz, 2H), 7.56–7.51 (m, 2H), 7.19 (d, *J* = 8.4 Hz, 1H), 7.10 (t, *J* = 7.2 Hz, 1H), and 3.86 (s, 3H); ^13^C NMR (151 MHz, DMSO-d_6_) δ 161.3, 157.2, 152.4, 148.9, 134.4, 132.2, 130.5, 127.3, 126.6, 125.8, 122.6, 120.9, 120.5, 111.9, and 55.8.
*2-*(*o-Tolyl*)*quinazolin-4*(*3H*)*-one* (**3ad**) [63]: The title compound was synthesized according to general procedure A; White solid; Yield: 65% (77 mg); ^1^H NMR (600 MHz, DMSO-d_6_) δ 12.43 (s, 1H), 8.17 (d, *J* = 7.8 Hz, 1H), 7.83 (t, *J* = 7.2 Hz, 1H), 7.69 (d, *J* = 7.8 Hz, 1H), 7.54 (d, *J* = 7.2 Hz, 1H), 7.53–7.49 (m, 1H), 7.43 (t, *J* = 7.2 Hz, 1H), 7.36–7.30 (m, 2H), and 2.39 (s, 3H); ^13^C NMR (151 MHz, DMSO-d_6_) δ 161.8, 154.4, 148.7, 136.1, 134.4, 134.2, 130.5, 129.9, 129.1, 127.4, 126.6, 125.8, 125.7, 120.9, and 19.5.
*2-*(*m-Tolyl*)*quinazolin-4*(*3H*)*-one* (**3ae**) [23]: The title compound was synthesized according to general procedure A; White solid; Yield: 74% (88 mg); ^1^H NMR (600 MHz, DMSO-d_6_) δ 12.45 (s, 1H), 8.15 (d, *J* = 7.2 Hz, 1H), 8.02 (s, 1H), 7.97 (d, *J* = 7.8 Hz, 1H), 7.85–7.81 (m, 1H), 7.74 (d, *J* = 7.8 Hz, 1H), 7.54–7.49 (m, 1H), 7.45–7.37 (m, 2H), and 2.40 (s, 3H); ^13^C NMR (151 MHz, DMSO-d_6_) δ 162.2, 152.4, 148.8, 137.9, 134.6, 132.7, 132.0, 128.5, 128.3, 127.5, 126.5, 125.9, 124.9, 120.9, and 20.9.
*2-*(*4-Fluorophenyl*)*quinazolin-4*(*3H*)*-one* (**3af**) [64]: The title compound was synthesized according to general procedure A; White solid; Yield: 83% (99.5 mg); ^1^H NMR (600 MHz, DMSO-d_6_) δ 12.55 (s, 1H), 8.27–8.22 (m, 2H), 8.15 (d, *J* = 7.8 Hz, 1H), 7.82 (t, *J* = 7.2 Hz, 1H), 7.72 (d, *J* = 8.4 Hz, 1H), 7.51 (t, *J* = 7.2 Hz, 1H), and 7.38 (t, *J* = 8.4 Hz, 2H); ^13^C NMR (151 MHz, DMSO-d_6_) δ 164.1 (d, ^1^*J_C-F_* = 249.4 Hz), 162.2, 151.4, 148.7, 134.6, 130.4 (d, ^3^*J_C-F_* = 9.0 Hz), 129.2 (d, ^4^*J_C-F_* = 2.5 Hz), 127.5, 126.6, 125.9, 120.9, and 115.6 (d, ^2^*J_C-F_* = 21.9 Hz).
*2-*(*2-Chlorophenyl*)*quinazolin-4*(*3H*)*-one* (**3ag**) [65]: The title compound was synthesized according to general procedure A; White solid; Yield: 63% (81 mg); ^1^H NMR (600 MHz, DMSO-d_6_) δ 12.65 (s, 1H), 8.18 (dd, *J* = 7.8, 1.2 Hz, 1H), 7.88–7.82 (m, 1H), 7.71 (d, *J* = 7.8 Hz, 1H), 7.67 (dd, *J* = 7.8, 1.2 Hz, 1H), 7.63–7.60 (m, 1H), 7.58–7.55 (m, 2H), and 7.50 (td, *J* = 7.8, 1.2 Hz, 1H); ^13^C NMR (151 MHz, DMSO-d_6_) δ 161.5, 152.3, 148.5, 134.6, 133.8, 131.6, 131.5, 130.9, 129.6, 127.4, 127.2, 127.1, 125.9, and 121.2.
*2-*(*4-Bromophenyl*)*quinazolin-4*(*3H*)*-one* (**3ai**) [21]: The reaction was performed in DMSO solvent and reaction mixture was poured into water to get the solid precipitate. Further crude product was washed with excess water to obtain the pure product. White solid; Yield: 86% (129 mg); ^1^H NMR (600 MHz, DMSO-d_6_) δ 12.60 (s, 1H), 8.15 (dd, *J* = 7.8, 1.2 Hz, 1H), 8.13 (s, 1H), 8.11 (s, 1H), 7.86–7.81 (m, 1H), 7.76 (d, *J* = 1.8 Hz, 1H), 7.75 (d, *J* = 2.4 Hz, 1H), 7.73 (s, 1H), and 7.55–7.51 (m, 1H); ^13^C NMR (151 MHz, DMSO-d_6_) δ 162.2, 151.5, 148.5, 134.7, 131.9, 131.6, 129.8, 127.4, 126.8, 125.9, 125.2, and 120.9.
*2-*(*[1,1′-Biphenyl]-4-yl*)*quinazolin-4*(*3H*)*-one* (**3aj**) [23]: The title compound was synthesized according to general procedure A; White solid; Yield: 81% (120.5 mg); ^1^H NMR (600 MHz, DMSO-d_6_) δ 12.58 (s, 1H), 8.30 (d, *J* = 7.8 Hz, 2H), 8.17 (d, *J* = 7.8 Hz, 1H), 7.84 (t, *J* = 9.0 Hz, 3H), 7.76 (d, *J* = 3.6 Hz, 3H), 7.56–7.47 (m, 3H), and 7.42 (t, *J* = 7.2 Hz, 1H); ^13^C NMR (151 MHz, DMSO-d_6_) δ 162.3, 151.9, 148.7, 142.9, 138.9, 134.6, 131.5, 129.1, 128.4, 128.2, 127.4, 126.8, 126.7, 126.6, 125.9, and 120.9.
*2-*(*Thiophen-2-yl*)*quinazolin-4*(*3H*)*-one* (**3ak**) [66]: The title compound was synthesized according to general procedure A; White solid; Yield: 67% (76 mg); ^1^H NMR (600 MHz, DMSO-d_6_) δ 12.48 (s, 1H), 8.12 (d, *J* = 7.8 Hz, 1H), 7.99 (s, 1H), 7.80 (t, *J* = 7.6 Hz, 1H), 7.68 (d, *J* = 8.1 Hz, 1H), 7.63 (d, *J* = 2.7 Hz, 1H), 7.48 (t, *J* = 7.5 Hz, 1H), and 6.74 (s, 1H); ^13^C NMR (151 MHz, DMSO-d_6_) δ 161.6, 148.7, 146.6, 146.1, 144.0, 134.6, 127.2, 126.4, 125.9, 121.2, 114.5, and 112.5.
*2-Hexylquinazolin-4*(*3H*)*-one* (**3al**) [67]: The title compound was synthesized according to general procedure A; White solid; Yield: 73% (84 mg); ^1^H NMR (600 MHz, DMSO-d_6_) δ 12.14 (s, 1H), 8.07 (dd, *J* = 7.8, 1.2 Hz, 1H), 7.77–7.72 (m, 1H), 7.58 (d, *J* = 7.8 Hz, 1H), 7.46–7.41 (m, 1H), 2.60–2.55 (m, 2H), 1.73–1.66 (m, 2H), 1.31–1.26 (m, 2H), 1.27–1.19 (m, 4H), and 0.86–0.79 (m, 3H); ^13^C NMR (151 MHz, DMSO-d_6_) δ 161.9, 157.6, 149.0, 134.3, 126.8, 125.9, 125.7, 120.8, 34.6, 30.9, 28.2, 26.8, 21.9, and 13.9.
*2-Decylquinazolin-4*(*3H*)*-one* (**3am**) [68]: The title compound was synthesized according to general procedure A; White solid; Yield: 68% (97 mg); ^1^H NMR (600 MHz, DMSO-d_6_) δ 12.13 (s, 1H), 8.07 (dd, *J* = 7.8, 1.2 Hz, 1H), 7.78–7.73 (m, 1H), 7.57 (d, *J* = 7.8 Hz, 1H), 7.46–7.41 (m, 1H), 2.61–2.54 (m, 2H), 1.73–1.66 (m, 2H), 1.33–1.25 (m, 5H), 1.25–1.14 (m, 9H), and 0.84–0.81 (m, 3H); ^13^C NMR (151 MHz, DMSO-d_6_) δ 161.9, 157.6, 148.9, 134.3, 126.8, 125.9, 125.7, 120.8, 34.5, 31.3, 28.9, 28.9, 28.7, 28.5, 26.8, 22.1, and 13.9.
*6-Fluoro-2-phenylquinazolin-4*(*3H*)*-one* (**3ba**) [69]: The title compound was synthesized according to general procedure A; White solid; Yield: 89% (107 mg); 89%; ^1^H NMR (600 MHz, DMSO-d_6_) δ 12.65 (s, 1H), 8.17 (s, 1H), 8.16–8.15 (m, 1H), 7.84–7.82 (m, 1H), 7.81 (d, *J* = 5.6 Hz, 1H), 7.72 (td, *J* = 8.4, 3.0 Hz, 1H), 7.59 (d, *J* = 7.2 Hz, 1H), and 7.56–7.53 (m, 2H); ^13^C NMR (151 MHz, DMSO-d_6_) δ 161.6, 159.9 (d, ^1^*J_C-F_* = 245.5 Hz), 151.8, 145.6, 132.6, 131.4, 130.3 (d, ^3^*J_C-F_* = 8.3 Hz), 128.6, 127.7, 123.1 (d, ^2^*J_C-F_* = 24.1 Hz), 122.2 (d, ^3^*J_C-F_* = 8.6 Hz), and 110.5 (d, ^2^*J_C-F_* = 23.3 Hz).
*6-Fluoro-2-*(*4-methoxyphenyl*)*quinazolin-4*(*3H*)*-one* (**3bb**) **[19]**: The title compound was synthesized according to general procedure A; White solid; Yield: 82% (111 mg); ^1^H NMR (600 MHz, DMSO-d_6_) δ 12.51 (s, 1H), 8.17 (s, 2H), 7.83–7.66 (m, 3H), 7.09 (s, 2H), and 3.85 (s, 3H); ^13^C NMR (151 MHz, DMSO-d_6_) δ 161.9, 159.7 (d, ^1^*J_C-F_* = 245.7 Hz), 151.5, 145.8, 130.0, 129.4, 124.6, 123.1, 122.9, 121.8 (d, ^3^*J_C-F_* = 10.6 Hz), 114.0, 110.4 (d, ^2^*J_C-F_* = 23.4 Hz), and 55.5.
*6-Fluoro-2-*(*2-methoxyphenyl*)*quinazolin-4*(*3H*)*-one* (**3bc**) [19]: The title compound was synthesized according to general procedure A; White solid; Yield: 78% (106 mg); ^1^H NMR (600 MHz, DMSO-d_6_) δ 12.22 (s, 1H), 7.82 (dd, *J* = 8.4, 3.0 Hz, 1H), 7.79–7.76 (m, 1H), 7.73–7.70 (m, 1H), 7.69–7.67 (m, 1H), 7.56–7.52 (m, 1H), 7.19 (d, *J* = 8.4 Hz, 1H), 7.09 (t, *J* = 7.2 Hz, 1H), and 3.86 (s, 3H); ^13^C NMR (151 MHz, DMSO-d_6_) δ 160.7, 159.9 (d, ^1^*J_C-F_* = 245.3 Hz), 157.1, 151.9, 145.9, 132.3, 130.4, 130.2 (d, ^3^*J_C-F_* = 8.3 Hz), 122.9 (d, ^2^*J_C-F_* = 24.0 Hz), 122.5, 122.2 (d, ^3^*J_C-F_* = 8.4 Hz), 120.4, 111.9, 110.4 (d, ^2^*J_C-F_* = 23.3 Hz), and 55.8.
*6-Fluoro-2-*(*m-tolyl*)*quinazolin-4*(*3H*)*-one* (**3be**) The title compound was synthesized according to general procedure A; White solid; Yield: 87% (110 mg); ^1^H NMR (600 MHz, DMSO-d_6_) δ 12.58 (s, 1H), 8.00 (s, 1H), 7.95 (d, *J* = 6.6 Hz, 1H), 7.81 (d, *J* = 6.0 Hz, 2H), 7.72 (d, *J* = 6.0 Hz, 1H), 7.46–7.37 (m, 2H), and 2.40 (s, 3H); ^13^C NMR (151 MHz, DMSO-d_6_) δ 161.7, 159.9 (d, ^1^*J_C-F_* = 244.8 Hz), 151.9, 145.6, 137.9, 132.5, 132.0, 130.3, 130.2 (d, ^4^*J_C-F_* = 4.9 Hz), 128.4 (d, ^3^*J_C-F_* = 7.9 Hz), 124.9, 123.1 (d, ^2^*J_C-F_* = 24.2 Hz), 122.2 (d, ^3^*J_C-F_* = 8.7 Hz), and 110.5 (d, ^2^*J_C-F_* = 23.2 Hz); FTIR $\tilde{\nu }$max = 3116, 3075, 1675, 1571, 1484, 1294, 879 cm^−1^; HRMS: calculated for C_15_H_12_FN_2_O: 255.0928 [M+H]^+^; found: 255.0939.
*2-*(*2-Chlorophenyl*)*-6-fluoroquinazolin-4*(*3H*)*-one* (**3bg**)**:** The title compound was synthesized according to general procedure A; White solid; Yield: 67% (92 mg); ^1^H NMR (600 MHz, DMSO-d_6_) δ 12.76 (s, 1H), 7.85 (dd, *J* = 8.4, 3.0 Hz, 1H), 7.80 (dd, *J* = 8.4, 4.8 Hz, 1H), 7.74 (td, *J* = 8.4, 3.0 Hz, 1H), 7.67 (dd, *J* = 7.8, 1.8 Hz, 1H), 7.63–7.60 (m, 1H), 7.57 (td, *J* = 7.8, 1.8 Hz, 1H), and 7.50 (td, *J* = 7.8, 1.2 Hz, 1H); ^13^C NMR (151 MHz, DMSO-d_6_) δ 160.9, 160.3 (d, ^1^*J_C-F_* = 246.0 Hz), 151.8, 145.4, 133.6, 131.7, 131.5, 130.9, 130.4 (d, ^3^*J_C-F_* = 10.0 Hz), 129.6, 127.3, 123.1 (d, ^2^*J_C-F_* = 24.1 Hz), 122.5 (d, ^3^*J_C-F_* = 8.4 Hz), and 110.6 (d, ^2^*J_C-F_* = 23.3 Hz); FTIR $\tilde{\nu }$max = 3045, 2977, 1679, 1604, 1481, 927, 763 cm^−1^; HRMS: calculated for C_14_H_9_ClFN_2_O: 275.0387 [M+H]^+^; found: 275.0396.
*2-*(*[1,1′-Biphenyl]-4-yl*)*-6-fluoroquinazolin-4*(*3H*)*-one* (**3bj**): The title compound was synthesized according to general procedure A; White solid; Yield: 58% (92 mg); ^1^H NMR (600 MHz, DMSO-d_6_) δ 12.70 (s, 1H), 8.29 (s, 2H), 7.86 (s, 4H), 7.78 (s, 3H), and 7.55–7.49 (m, 3H); ^13^C NMR (151 MHz, DMSO-d_6_) δ 161.7, 159.9 (d, ^1^*J_C-F_* = 246.1 Hz), 157.4, 154.1, 151.5, 145.7, 142.9, 138.9, 130.3, 129.1, 128.4, 128.2, 126.8 (d, ^3^*J_C-F_* = 13.3 Hz), 123.1 (d, ^2^*J_C-F_* = 23.5 Hz), 122.2, and 110.5 (d, ^2^*J_C-F_* = 22.4 Hz); FTIR $\tilde{\nu }$max = 3029, 2952, 1660, 1596, 1481, 1301, 836 cm^−1^; HRMS: calculated for C_20_H_14_FN_2_O: 317.1090 [M+H]^+^; found: 317.1103.
*6-Chloro-2-phenylquinazolin-4*(*3H*)*-one* (**3ca**) [21]: The title compound was synthesized according to general procedure A; White solid; Yield: 69% (89 mg); ^1^H NMR (600 MHz, DMSO-d_6_) δ 12.71 (s, 1H), 8.18 (s, 2H), 8.09 (s, 1H), 7.86 (s, 1H), 7.77 (s, 1H), 7.60 (s, 1H), and 7.56 (s, 2H); ^13^C NMR (151 MHz, DMSO-d_6_) δ 161.3, 152.9, 147.5, 134.7, 132.4, 131.6, 130.8, 129.7, 128.6, 127.8, 124.9, and 122.2.
*6-Chloro-2-*(*4-methoxyphenyl*)*quinazolin-4*(*3H*)*-one* (**3cb**) [70]: The title compound was synthesized according to general procedure A; White solid; Yield: 58% (83 mg); ^1^H NMR (600 MHz, DMSO-d_6_) δ 12.56 (s, 1H), 8.19 (s, 1H), 8.17 (s, 1H), 8.06 (d, *J* = 2.4 Hz, 1H), 7.83 (dd, *J* = 8.4, 2.4 Hz, 1H), 7.72 (d, *J* = 8.4 Hz, 1H), 7.09 (d, *J* = 8.8 Hz, 2H), and 3.85 (s, 3H); ^13^C NMR (151 MHz, DMSO-d_6_) δ 162.0, 161.3, 152.4, 147.7, 134.6, 130.2, 129.6, 129.5, 124.8, 124.5, 121.9, 114.0, and 55.5.
*6-Chloro-2-*(*2-methoxyphenyl*)*quinazolin-4*(*3H*)*-one* (**3cc**) **[71]**: The title compound was synthesized according to general procedure A; White solid; Yield: 59% (85 mg); ^1^H NMR (600 MHz, DMSO-d_6_) δ 12.27 (s, 1H), 8.08 (d, *J* = 2.4 Hz, 1H), 7.85 (dd, *J* = 8.4, 2.4 Hz, 1H), 7.73 (d, *J* = 8.4 Hz, 1H), 7.70 (dd, *J* = 7.8, 1.7 Hz, 1H), 7.56–7.51 (m, 1H), 7.20 (d, *J* = 8.4 Hz, 1H), 7.09 (t, *J* = 7.8 Hz, 1H), and 3.86 (s, 3H); ^13^C NMR (151 MHz, DMSO-d_6_) δ 160.3, 157.1, 152.8, 147.8, 134.5, 132.4, 130.8, 130.5, 129.6, 124.8, 122.4, 122.2, 120.4, 111.9, and 55.8.
*6-Chloro-2-*(*o-tolyl*)*quinazolin-4*(*3H*)*-one* (**3cd**) [72]: The title compound was synthesized according to general procedure A; White solid; Yield: 53% (72 mg); ^1^H NMR (600 MHz, DMSO-d_6_) δ 12.61 (s, 1H), 8.10 (d, *J* = 2.4 Hz, 1H), 7.86 (dd, *J* = 8.4, 2.4 Hz, 1H), 7.71 (d, *J* = 8.4 Hz, 1H), 7.51 (d, *J* = 7.2 Hz, 1H), 7.44 (t, *J* = 7.2 Hz, 1H), 7.35 (d, *J* = 8.4 Hz, 1H), 7.32 (d, *J* = 7.2 Hz, 1H), and 2.38 (s, 3H); ^13^C NMR (151 MHz, DMSO-d_6_) δ 160.8, 154.9, 147.4, 136.2, 134.5, 133.9, 130.8, 130.6, 130.0, 129.6, 129.1, 125.7, 124.8, 122.2, and 19.5.
*6-Chloro-2-*(*m-tolyl*)*quinazolin-4*(*3H*)*-one* (**3ce**): The title compound was synthesized according to general procedure A; White solid; Yield: 56% (76 mg); ^1^H NMR (600 MHz, DMSO-d_6_) δ 12.63 (s, 1H), 8.08 (d, *J* = 2.4 Hz, 1H), 8.01 (s, 1H), 7.96 (d, *J* = 7.2 Hz, 1H), 7.85 (dd, *J* = 8.4, 2.4 Hz, 1H), 7.76 (d, *J* = 8.4 Hz, 1H), 7.45–7.40 (m, 2H), and 2.41 (s, 3H); ^13^C NMR (151 MHz, DMSO-d_6_) δ 161.3, 152.9, 137.9, 134.8, 134.7, 132.9, 132.4, 132.2, 130.7, 129.7, 128.5, 128.4, 124.9, 124.9, and 20.9; FTIR $\tilde{\nu }$max = 3023, 2925, 1671, 1579, 1467, 1309, 842 cm^−1^; HRMS: calculated for C_15_H_12_ClN_2_O: 271.0638 [M+H]^+^; found: 271.0649.
*6-Chloro-2-*(*4-fluorophenyl*)*quinazolin-4*(*3H*)*-one* (**3cf**) [73]: The title compound was synthesized according to general procedure A; White solid; Yield: 65% (89 mg); ^1^H NMR (600 MHz, DMSO-d_6_) δ 12.73 (s, 1H), 8.26–8.21 (m, 2H), 8.08 (d, *J* = 2.4 Hz, 1H), 7.85 (dd, *J* = 8.4, 2.4 Hz, 1H), 7.75 (d, *J* = 8.4 Hz, 1H), and 7.39 (t, *J* = 8.4 Hz, 2H); ^13^C NMR (151 MHz, DMSO-d_6_) δ 164.1 (d, ^1^*J_C-F_* = 249.9 Hz), 161.3, 151.9, 147.4, 134.7, 130.8, 130.5 (d, ^3^*J_C-F_* = 9.0 Hz), 129.7, 128.9 (d, ^4^*J_C-F_* = 2.8 Hz), 124.9, 122.1, and 115.7 (d, ^2^*J_C-F_* = 22.0 Hz).
*6-Chloro-2-*(*2-chlorophenyl*)*quinazolin-4*(*3H*)*-one* (**3cg**) [73]: The title compound was synthesized according to general procedure A; White solid; Yield: 61% (89 mg); ^1^H NMR (600 MHz, DMSO-d_6_) δ 12.82 (s, 1H), 8.12 (d, *J* = 2.4 Hz, 1H), 7.89 (dd, *J* = 8.4, 2.4 Hz, 1H), 7.75 (d, *J* = 8.4 Hz, 1H), 7.67 (dd, *J* = 7.2, 1.2 Hz, 1H), 7.62 (d, *J* = 8.4 Hz, 1H), 7.58 (td, *J* = 7.2, 1.2 Hz, 1H), and 7.50 (t, *J* = 7.2 Hz, 1H); ^13^C NMR (151 MHz, DMSO-d_6_) δ 160.5, 152.7, 147.3, 134.7, 133.5, 131.8, 131.4, 131.4, 130.9, 129.7, 129.6, 127.2, 124.9, and 122.5.
*6-Chloro-2-*(*4-chlorophenyl*)*quinazolin-4*(*3H*)*-one* (**3ch**) [73]: The title compound was synthesized according to general procedure A; White solid; Yield: 64% (93 mg); ^1^H NMR (600 MHz, DMSO-d_6_) δ 12.67 (s, 1H), 8.20 (d, *J* = 7.8 Hz, 2H), 8.09 (s, 1H), 7.85 (d, *J* = 8.4 Hz, 1H), 7.76 (d, *J* = 8.4 Hz, 1H), and 7.62 (d, *J* = 7.8 Hz, 2H); ^13^C NMR (151 MHz, DMSO-d_6_) δ 161.3, 152.3, 141.4, 139.2, 136.3, 134.4, 129.8, 129.5, 128.5, 127.6, 124.7, and 122.2.
*2-*(*4-Bromophenyl*)*-6-chloroquinazolin-4*(*3H*)*-one* (**3ci**) [74]: The title compound was synthesized according to general procedure A; White solid; Yield: 76% (127 mg); ^1^H NMR (600 MHz, DMSO-d_6_) δ 12.66 (s, 1H), 8.14 (s, 1H), 8.12 (s, 1H), 8.08 (d, *J* = 2.5 Hz, 1H), 7.84 (dd, *J* = 8.7, 2.5 Hz, 1H), and 7.76 (d, *J* = 8.4 Hz, 3H); ^13^C NMR (151 MHz, DMSO-d_6_) δ 161.4, 152.2, 134.4, 131.4, 131.4, 130.6, 129.7, 129.4, 128.8, 125.1, 124.7, and 122.2.
*1′H-spiro[cyclopentane-1,2′-quinazolin]-4′*(*3′H*)*-one* (**3an**) [75] The title compound was synthesized according to general procedure A; White solid; Yield: 56% (56.6 mg); ^1^H NMR (600 MHz, DMSO-d_6_) δ 8.07 (s, 1H), 7.57 (dd, *J* = 7.8, 1.2 Hz, 1H), 7.25–7.14 (m, 1H), 6.72 (s, 1H), 6.69 (d, *J* = 7.8 Hz, 1H), 6.63 (t, *J* = 7.8 Hz, 1H), 1.83–1.75 (m, 4H), and 1.68–1.63 (m, 4H); ^13^C NMR (151 MHz, DMSO-d_6_) δ 163.5, 147.5, 133.0, 127.3, 116.6, 114.6, 114.3, 77.1, 39.3, and 21.9.
*1′H-spiro[cyclohexane-1,2′-quinazolin]-4′*(*3′H*)*-one* (**3ao**) [75]: The title compound was synthesized according to general procedure A; White solid; Yield: 73% (79 mg); ^1^H NMR (600 MHz, DMSO-d_6_) δ 7.90 (s, 1H), 7.56 (dd, *J* = 7.8, 1.2 Hz, 1H), 7.24–7.16 (m, 1H), 6.80 (d, *J* = 7.8 Hz, 1H), 6.62 (d, *J* = 7.8 Hz, 1H), 6.60 (d, *J* = 5.4 Hz, 1H), 1.78–1.67 (m, 2H), 1.65–1.58 (m, 2H), 1.58–1.51 (m, 4H), 1.46–1.36 (m, 1H), and 1.29–1.20 (m, 1H); ^13^C NMR (151 MHz, DMSO-d_6_) δ 163.2, 146.8, 133.1, 127.1, 116.5, 114.6, 114.5, 67.8, 37.2, 24.6, and 20.9.
*1′H-spiro[cycloheptane-1,2′-quinazolin]-4′*(*3′H*)*-one* (**3ap**) [75]: The reaction was performed in DMSO solvent, and the reaction mixture was poured into water to get the solid precipitate. Further crude product was washed with excess water to obtain the pure product. White solid; Yield: 84% (97 mg); ^1^H NMR (600 MHz, DMSO-d_6_) δ 8.00 (s, 1H), 7.55 (dd, *J* = 7.8, 1.2 Hz, 1H), 7.23–7.16 (m, 1H), 6.71 (s, 1H), 6.70 (s, 1H), 6.62–6.58 (m, 1H), 1.92–1.81 (m, 4H), and 1.51 (s, 8H); ^13^C NMR (151 MHz, DMSO-d_6_) δ 162.9, 146.7, 133.1, 127.1, 116.3, 114.4, 114.3, 71.9, 41.0, 29.2, and 20.9.
*6′-Chloro-1′H-spiro[cyclohexane-1,2′-quinazolin]-4′*(*3′H*)*-one* (**3co**) [75]: The title compound was synthesized according to general procedure A; White solid; Yield: 86% (108 mg); ^1^H NMR (600 MHz, DMSO-d_6_) δ 8.10 (s, 1H), 7.49 (d, *J* = 2.4 Hz, 1H), 7.24 (dd, *J* = 8.4, 2.4 Hz, 1H), 6.87–6.78 (m, 2H), 1.78–1.70 (m, 2H), 1.63–1.57 (m, 2H), 1.57–1.50 (m, 4H), 1.47–1.38 (m, 1H), and 1.27–1.19 (m, 1H); ^13^C NMR (151 MHz, DMSO-d_6_) δ 162.0, 145.5, 132.9, 126.2, 120.1, 116.6, 115.6, 68.0, 37.1, 24.5, and 20.8.

## 4. Conclusions

In conclusion, the employment of water as a green solvent in the microwave-assisted synthesis of quinazolinones utilizing aldehyde bisulfite adducts represents a significant advancement in developing sustainable and efficient chemical processes. This innovative approach not only achieves high product yields but also streamlines the purification process, fully embodying the principles of green chemistry. It was also supported by green metrics and Eco-Scale score calculations. Remarkably, the broad library scope includes the compounds **3be**, **3bg**, **3bj**, and **3ce**, which were prepared in favorable yields but have not been previously reported in the literature. Water as a solvent is instrumental in minimizing environmental impacts and enhancing procedural efficiency, underscoring its vital role in fostering eco-friendly synthetic strategies. This method enables the construction of quinazolinone frameworks while eliminating the need for column chromatography, further reducing waste and energy consumption. Such a methodology marks a progressive step towards greener and more sustainable chemical production, contributing to ongoing efforts to achieve a cleaner and more sustainable future in chemical manufacturing.

## Figures and Tables

**Table 1:** Optimization of the reaction conditions ^a^.

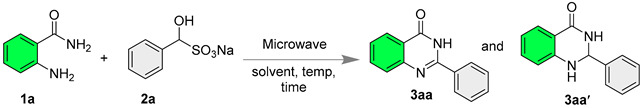				
Entry	Temp (°C)	Solvent	Time (h)	Conversion (3aa: 3aa′) ^b^
1	80	H_2_O	8	15:85
2	90	H_2_O	8	65:35
3	100	H_2_O	6	80:20
4	100	H_2_O	8	98:2
5	100	H_2_O	10	>99 (91%) ^c^
6	25	H_2_O	10	-
7	100	-	10	80:12
8	100	DMSO	10	>99 (86%) ^c^
9	100	Toluene	10	47:35

^a^ Reaction condition: All the reactions were performed using 0.5 mmol of anthranilamide (**1a**) and 0.5 mmol of sodium hydroxy(phenyl)methanesulfonate (**2a**) in 2 mL of solvent at 100 °C which was stirred for 10 h. ^b^ Yields were determined by NMR analysis. ^c^ Isolated yield after filtration.

## Data Availability

The data presented in this study are available in article and Supplementary Materials.

## Supplementary Materials

The following supporting information can be downloaded at: https://www.mdpi.com/article/10.3390/molecules29091986/s1, including general information, the synthesis of the compounds, the characterization data, and the NMR spectra.

## References

[1] Arcadi A., Morlacci V., Palombi L. (2023). Synthesis of Nitrogen-Containing Heterocyclic Scaffolds through Sequential Reactions of Aminoalkynes with Carbonyls. Molecules.

[2] Majee S., Shilpa, Sarav M., Banik B.K., Ray D. (2023). Recent Advances in the Green Synthesis of Active *N*-Heterocycles and Their Biological Activities. Pharmaceuticals.

[3] Yu H., Xu F. (2023). Advances in the synthesis of nitrogen-containing heterocyclic compounds by in situ benzyne cycloaddition. RSC Adv..

[4] Sun Z., Bottari G., Barta K. (2015). Supercritical methanol as solvent and carbon source in the catalytic conversion of 1,2-diaminobenzenes and 2-nitroanilines to benzimidazoles. Green Chem..

[5] Abdullaha M., Mohammed S., Ali M., Kumar A., Vishwakarma R.A., Bharate S.B. (2019). Discovery of Quinazolin-4(3 *H*)-ones as NLRP3 Inflammasome Inhibitors: Computational Design, Metal-Free Synthesis, and in Vitro Biological Evaluation. J. Org. Chem..

[6] Ma J., Chen L., Fan J., Cao W., Zeng G., Wang Y., Li Y., Zhou Y., Deng X. (2019). Dual-targeting Rutaecarpine-NO donor hybrids as novel anti-hypertensive agents by promoting release of CGRP. Eur. J. Med. Chem..

[7] Gatadi S., Lakshmi T.V., Nanduri S. (2019). 4(3*H*)-Quinazolinone derivatives: Promising antibacterial drug leads. Eur. J. Med. Chem..

[8] Bai S., Feng S., Zhang W., Mou H., Tang S., Zhou H., Luo J., Wei X., Zhu Y., Wu R. (2024). Design, synthesis and antibacterial activity of quinazolinone derivatives containing glycosides. Mol. Cryst. Liq. Cryst..

[9] Xia Y., Yang Z.-Y., Hour M.-J., Kuo S.-C., Xia P., Bastow K.F., Nakanishi Y., Nampoothiri P., Hackl T., Hamel E. (2001). Antitumor Agents. Part 204:1 Synthesis and Biological Evaluation of Substituted 2-Aryl Quinazolinones. Bioorg. Med. Chem. Lett..

[10] Noser A.A., El-Barbary A.A., Salem M.M., El Salam H.A.A., shahien M. (2024). Synthesis and molecular docking simulations of novel azepines based on quinazolinone moiety as prospective antimicrobial and antitumor hedgehog signaling inhibitors. Sci. Rep..

[11] Balakumar C., Lamba P., Pran Kishore D., Lakshmi Narayana B., Venkat Rao K., Rajwinder K., Raghuram Rao A., Shireesha B., Narsaiah B. (2010). Synthesis, anti-inflammatory evaluation and docking studies of some new fluorinated fused quinazolines. Eur. J. Med. Chem..

[12] Kim S.J., Lee S.J., Lee S., Chae S., Han M.D., Mar W., Nam K.W. (2009). Rutecarpine ameliorates bodyweight gain through the inhibition of orexigenic neuropeptides NPY and AgRP in mice. Biochem. Biophys. Res. Commun..

[13] Dai J.-R., Carté B.K., Sidebottom P.J., Sek Yew A.L., Ng S.-B., Huang Y., Butler M.S. (2001). Circumdatin G, a New Alkaloid from the Fungus Aspergillus ochraceus. J. Nat. Prod..

[14] Chen Z., Hu G., Li D., Chen J., Li Y., Zhou H., Xie Y. (2009). Synthesis and vasodilator effects of rutaecarpine analogues which might be involved transient receptor potential vanilloid subfamily, member 1 (TRPV1). Biorg. Med. Chem..

[15] Xu Y.-H., Hu Y.-T., Xu S.-M., Song B.-B., Yuan H., Zhao D.-D., Guo S.-Y., Jiang Z., Wei L.-Y., Rao Y. (2023). Design and Synthesis of Bouchardatine Derivatives as a Novel AMP-Activated Protein Kinase Activator for the Treatment of Colorectal Cancer. J. Med. Chem..

[16] Wang R.-X., Du S.-S., Wang J.-R., Chu Q.-R., Tang C., Zhang Z.-J., Yang C.-J., He Y.-H., Li H.-X., Wu T.-L. (2021). Design, Synthesis, and Antifungal Evaluation of Luotonin A Derivatives against Phytopathogenic Fungi. J. Agric. Food. Chem..

[17] Sarfraz M., Wang C., Sultana N., Ellahi H., Rehman M.F.u., Jameel M., Akhter S., Kanwal F., Tariq M.I., Xue S. (2021). 2,3-Dihydroquinazolin-4(1*H*)-one as a New Class of Anti-Leishmanial Agents: A Combined Experimental and Computational Study. Crystals.

[18] Kshirsagar U., Rohokale R. (2016). Advanced Synthetic Strategies for Constructing Quinazolinone Scaffolds. Synthesis.

[19] Liu Z., Zeng L.Y., Li C., Yang F., Qiu F., Liu S., Xi B. (2018). “On-Water” Synthesis of Quinazolinones and Dihydroquinazolinones Starting from o-Bromobenzonitrile. Molecules.

[20] Hakim Siddiki S.M.A., Kon K., Touchy A.S., Shimizu K.-i. (2014). Direct synthesis of quinazolinones by acceptorless dehydrogenative coupling of o-aminobenzamide and alcohols by heterogeneous Pt catalysts. Catal. Sci. Technol..

[21] Bera S.K., Bhanja R., Mal P. (2022). DDQ in mechanochemical C–N coupling reactions. Beilstein J. Org. Chem..

[22] Bera S.K., Mal P. (2022). Regiodivergent C–N Coupling of Quinazolinones Controlled by the Dipole Moments of Tautomers. Org. Lett..

[23] Huang S., Jin L., Liu Y., Yang G., Wang A., Le Z., Jiang G., Xie Z. (2024). Visible light-mediated synthesis of quinazolinones from benzyl bromides and 2-aminobenzamides without using any photocatalyst or additive. Org. Biomol. Chem..

[24] Bhanja R., Bera S.K., Mal P. (2023). Regioselective synthesis of phenanthridine-fused quinazolinones using a 9-mesityl-10-methylacridinium perchlorate photocatalyst. Chem. Commun..

[25] Anastas P., Eghbali N. (2010). Green Chemistry: Principles and Practice. Chem. Soc. Rev..

[26] Kitanosono T., Masuda K., Xu P., Kobayashi S. (2018). Catalytic Organic Reactions in Water toward Sustainable Society. Chem. Rev..

[27] Li C.J., Chen L. (2006). Organic chemistry in water. Chem. Soc. Rev..

[28] Russo C., Brunelli F., Tron G.C., Giustiniano M. (2023). Visible-Light Photoredox Catalysis in Water. J. Org. Chem..

[29] Cortes-Clerget M., Yu J., Kincaid J.R.A., Walde P., Gallou F., Lipshutz B.H. (2021). Water as the reaction medium in organic chemistry: From our worst enemy to our best friend. Chem. Sci..

[30] Simon M.O., Li C.J. (2012). Green chemistry oriented organic synthesis in water. Chem. Soc. Rev..

[31] Khanna A., Dubey P., Sagar R. (2021). Exploiting Microwave-Assisted Organic Synthesis (MAOS) for Accessing Bioactive Scaffolds. Curr. Org. Chem..

[32] Varma R.S. (1999). Solvent-free organic syntheses. using supported reagents and microwave irradiation. Green Chem..

[33] Henary M., Kananda C., Rotolo L., Savino B., Owens E.A., Cravotto G. (2020). Benefits and applications of microwave-assisted synthesis of nitrogen containing heterocycles in medicinal chemistry. RSC Adv..

[34] Polshettiwar V., Varma R.S. (2008). Microwave-Assisted Organic Synthesis and Transformations using Benign Reaction Media. Acc. Chem. Res..

[35] Lidström P., Tierney J., Wathey B., Westman J. (2001). Microwave assisted organic synthesis—A review. Tetrahedron.

[36] Gawande M.B., Shelke S.N., Zboril R., Varma R.S. (2014). Microwave-Assisted Chemistry: Synthetic Applications for Rapid Assembly of Nanomaterials and Organics. Acc. Chem. Res..

[37] Martina K., Cravotto G., Varma R.S. (2021). Impact of Microwaves on Organic Synthesis and Strategies toward Flow Processes and Scaling Up. J. Org. Chem..

[38] Polshettiwar V., Varma R.S. (2008). Aqueous microwave chemistry: A clean and green synthetic tool for rapid drug discovery. Chem. Soc. Rev..

[39] Geetanjali, Singh R. (2021). Microwave-assisted Organic Synthesis in Water. Curr. Microw. Chem..

[40] Tiwari G., Khanna A., Mishra V.K., Sagar R. (2023). Recent developments on microwave-assisted organic synthesis of nitrogen- and oxygen-containing preferred heterocyclic scaffolds. RSC Adv..

[41] Nardi M., Bonacci S., Herrera Cano N., Oliverio M., Procopio A. (2022). The Highly Efficient Synthesis of 1,2-Disubstituted Benzimidazoles Using Microwave Irradiation. Molecules.

[42] Meera G., Rohit K.R., Saranya S., Anilkumar G. (2020). Microwave assisted synthesis of five membered nitrogen heterocycles. RSC Adv..

[43] Majumder A., Gupta R., Jain A. (2013). Microwave-assisted synthesis of nitrogen-containing heterocycles. Green Chem. Lett. Rev..

[44] Ranu B.C., Hajra A., Dey S.S., Jana U. (2003). Efficient microwave-assisted synthesis of quinolines and dihydroquinolines under solvent-free conditions. Tetrahedron.

[45] Ranu B.C., Hajra A., Jana U. (2000). Microwave-assisted simple synthesis of quinolines from anilines and alkyl vinyl ketones on the surface of silica gel in the presence of indium(III) chloride. Tetrahedron Lett..

[46] Adhikari A., Bhakta S., Ghosh T. (2022). Microwave-assisted synthesis of bioactive heterocycles: An overview. Tetrahedron.

[47] Banerjee B., Kaur G. (2020). Microwave Assisted Catalyst-free Synthesis of Bioactive Heterocycles. Curr. Microw. Chem..

[48] Saleem Q., Torabfam M., Fidan T., Kurt H., Yüce M., Clarke N., Bayazit M.K. (2021). Microwave-Promoted Continuous Flow Systems in Nanoparticle Synthesis—A Perspective. ACS Sustain. Chem. Eng..

[49] Karelou M., Kampasis D., Kalampaliki A.D., Persoons L., Krämer A., Schols D., Knapp S., De Jonghe S., Kostakis I.K. (2023). Synthesis and Biological Evaluation of 2-Substituted Quinazolin-4(3*H*)-Ones with Antiproliferative Activities. Molecules.

[50] He M., Beahm B.J., Bode J.W. (2008). Chiral NHC-Catalyzed Oxodiene Diels−Alder Reactions with α-Chloroaldehyde Bisulfite Salts. Org. Lett..

[51] Khosropour A.R., Khodaei M.M., Beygzadeh M. (2007). Highly convenient one-pot conversion of aryl acylals or aryl aldehyde bisulfites into dihydropyrimidones using BI(NO_3_)_3_·5H_2_O. Heteroat. Chem..

[52] Furigay M.H., Boucher M.M., Mizgier N.A., Brindle C.S. (2018). Separation of Aldehydes and Reactive Ketones from Mixtures Using a Bisulfite Extraction Protocol. J. Vis. Exp..

[53] Kissane M.G., Frank S.A., Rener G.A., Ley C.P., Alt C.A., Stroud P.A., Vaid R.K., Boini S.K., McKee L.A., Vicenzi J.T. (2013). Counterion effects in the preparation of aldehyde–bisulfite adducts. Tetrahedron Lett..

[54] Li X., Iyer K.S., Thakore R.R., Leahy D.K., Bailey J.D., Lipshutz B.H. (2021). Bisulfite Addition Compounds as Substrates for Reductive Aminations in Water. Org. Lett..

[55] Betti M., Genesio E., Marconi G., Sanna Coccone S., Wiedenau P. (2014). A Scalable Route to the SMO Receptor Antagonist SEN826: Benzimidazole Synthesis via Enhanced in Situ Formation of the Bisulfite–Aldehyde Complex. Org. Proc. Res. Dev..

[56] Behera S., Bera S.K., Basoccu F., Cuccu F., Caboni P., De Luca L., Porcheddu A. (2024). Application of Bertagnini’s Salts in a Mechanochemical Approach Toward Aza-Heterocycles and Reductive Aminations via Imine Formation. Adv. Synth. Catal..

[57] Badolato M., Aiello F., Neamati N. (2018). 2,3-Dihydroquinazolin-4(1*H*)-one as a privileged scaffold in drug design. RSC Adv..

[58] Jang Y., Lee S.B., Hong J., Chun S., Lee J., Hong S. (2020). Synthesis of 2-aryl quinazolinones via iron-catalyzed cross-dehydrogenative coupling (CDC) between N–H and C–H bonds. Org. Biomol. Chem..

[59] Tiwari A.R., Bhanage B.M. (2016). Synthesis of quinazolines from 2-aminobenzylamines with benzylamines and *N*-substituted benzylamines under transition metal-free conditions. Org. Biomol. Chem..

[60] Sankar M., Nowicka E., Carter E., Murphy D.M., Knight D.W., Bethell D., Hutchings G.J. (2014). The benzaldehyde oxidation paradox explained by the interception of peroxy radical by benzyl alcohol. Nat. Commun..

[61] Li Z., Dong J., Chen X., Li Q., Zhou Y., Yin S.-F. (2015). Metal- and Oxidant-Free Synthesis of Quinazolinones from β-Ketoesters with o-Aminobenzamides via Phosphorous Acid-Catalyzed Cyclocondensation and Selective C–C Bond Cleavage. J. Org. Chem..

[62] Sardar B., Jamatia R., Samanta A., Srimani D. (2022). Ru Doped Hydrotalcite Catalyzed Borrowing Hydrogen-Mediated *N*-Alkylation of Benzamides, Sulfonamides, and Dehydrogenative Synthesis of Quinazolinones. J. Org. Chem..

[63] Alam M.T., Maiti S., Mal P. (2018). The mechanochemical synthesis of quinazolin-4(3*H*)-ones by controlling the reactivity of IBX. Beilstein J. Org. Chem..

[64] Huang J., Chen W., Liang J., Yang Q., Fan Y., Chen M.-W., Peng Y. (2021). α-Keto Acids as Triggers and Partners for the Synthesis of Quinazolinones, Quinoxalinones, Benzooxazinones, and Benzothiazoles in Water. J. Org. Chem..

[65] Dutta B., Dutta N., Dutta A., Gogoi M., Mehra S., Kumar A., Deori K., Sarma D. (2023). [DDQM][HSO(4)]/TBHP as a Multifunctional Catalyst for the Metal Free Tandem Oxidative Synthesis of 2-Phenylquinazolin-4(3H)-ones. J. Org. Chem..

[66] Yang X., Cheng G., Shen J., Kuai C., Cui X. (2015). Cleavage of the C–C triple bond of ketoalkynes: Synthesis of 4(3H)-quinazolinones. Org. Chem. Front..

[67] Zhang Z., Wang M., Zhang C., Zhang Z., Lu J., Wang F. (2015). The cascade synthesis of quinazolinones and quinazolines using an α-MnO_2_ catalyst and tert-butyl hydroperoxide (TBHP) as an oxidant. Chem. Commun..

[68] Mahmoud M.R., El-Bordany E.A.A., Hassan N.F., El-Azm F.S.M.A. (2007). New 2,3-Disubstituted Quinazolin-4(3*H*)-Ones from 2-Undecyl-3,1-Benzoxazin-4-One. J. Chem. Res..

[69] Feng Y., Li Y., Cheng G., Wang L., Cui X. (2015). Copper-Catalyzed Synthesis of 2-Arylquinazolinones from 2-Arylindoles with Amines or Ammoniums. J. Org. Chem..

[70] Xu G., Wang L., Li M., Tao M., Zhang W. (2017). Phosphorous acid functionalized polyacrylonitrile fibers with a polarity tunable surface micro-environment for one-pot C–C and C–N bond formation reactions. Green Chem..

[71] Shang Y.-H., Fan L.-Y., Li X.-X., Liu M.-X. (2015). Y(OTf)_3_-catalyzed heterocyclic formation via aerobic oxygenation: An approach to dihydro quinazolinones and quinazolinones. Chin. Chem. Lett..

[72] Li H., He L., Neumann H., Beller M., Wu X.-F. (2014). Cascade synthesis of quinazolinones from 2-aminobenzonitriles and aryl bromides via palladium-catalyzed carbonylation reaction. Green Chem..

[73] Patel S.M., Chada H., Biswal S., Sharma S., Sharada D.S. (2019). Copper-Catalyzed Intramolecular α-C–H Amination via Ring-Opening Cyclization Strategy to Quinazolin-4-ones: Development and Application in Rutaecarpine Synthesis. Synthesis.

[74] Majumdar B., Sarma D., Jain S., Sarma T.K. (2018). One-Pot Magnetic Iron Oxide–Carbon Nanodot Composite-Catalyzed Cyclooxidative Aqueous Tandem Synthesis of Quinazolinones in the Presence of tert-Butyl Hydroperoxide. ACS Omega.

[75] Gnyawali K., Kirinde Arachchige P.T., Yi C.S. (2022). Synthesis of Flavanone and Quinazolinone Derivatives from the Ruthenium-Catalyzed Deaminative Coupling Reaction of 2′-Hydroxyaryl Ketones and 2-Aminobenzamides with Simple Amines. Org. Lett..

