# Supplementary material for: Unveiling the Untapped Potential of Bertagnini’s Salts in Microwave-Assisted Synthesis of Quinazolinones
Source: Molecules. 2024 Apr 26;29(9):1986. doi: 10.3390/molecules29091986 (PMC11085446; doi:10.3390/molecules29091986)
Supplement: Supplementary file 1 [file molecules-29-01986-s001.zip › molecules-2963558-supplementary.pdf]

molecules

SUPPORTING INFORMATION

Unveiling the Untapped Potential of Bertagnini's Salts in Microwave-Assisted Synthesis of Quinazolinones

Shyamal Kanti Bera, Sourav Behera, Lidia De Luca, Francesco Basoccu, Rita Mocci, and Andrea Porcheddu*

* Università degli Studi di Cagliari, Dipartimento di Scienze Chimiche e Geologiche, Cittadella Universitaria, 09042 Monserrato (CA), Italy.

E-mail: porcheddu@unica.it

TABLE OF CONTENT

GENERAL INFORMATION AND EXPERIMENTAL PROCEDURE 3

Table S1. Calculation of Ecoscale score[a] 7

Calculation of the Green Chemistry Metrics for the the microwave asssited synthesis of 2-Phenylquinazolin-4(3*H*)-one (3aa) 9

Calculation of the Green Chemistry Metrics for the reported synthesis of 2-Phenylquinazolin-4(3*H*)-one (3aa) 10

CHARACTERIZATION DATA OF THE SYNTHESIZED COMPOUNDS 11

NMR SPECTRA 22

References 55

GENERAL INFORMATION AND EXPERIMENTAL PROCEDURE

Commercially available reagents were purchased from Acros, Aldrich, Strem Chemicals, Alfa-Aesar, TCI Europe and used as received. All reactions were monitored by thin-layer chromatography (TLC) performed on glass-backed silica gel 60 F254, 0.2 mm plates (Merck), and compounds were visualized under UV light (254 nm). All reactions were performed using microwave instrument (Model: DISCOVER SP; SERIAL NO: DC8609; MODEL NO: 909155). The eluents were technical grade. ^{1}H and ^{13}C NMR spectra were recorded on a Varian 600 MHz and Bruker Avance III HD 600 MHz NMR spectrometer and were calibrated using trimethylsilane (TMS). Proton chemical shifts are expressed in parts per million (ppm, δ scale) and are referred to the residual hydrogen in the solvent ($CHCl_3$, 7.260 ppm or DMSO 2.50 ppm). Data are represented as follows: chemical shift, multiplicity (s = singlet, d = doublet, t = triplet, q = quartet, m = multiplet and/or multiple resonances, bs = broad singlet, and combination of thereof), coupling constant (*J*) in Hertz (Hz) and integration. Carbon chemical shifts are expressed in parts per million (ppm, δ scale) and are referenced to the carbon resonances of the NMR solvent ($CDCl_3$, δ 77.16 ppm or δ DMSO-d_6 δ 39.52 ppm). Deuterated NMR solvents were obtained from Aldrich. Infrared (IR) spectral was recorded in the Jasco FTIR-4X (MODEL: PKS-D1) instrument and data are reported in wavenumber (cm^{-1}). Positive ESI-MS spectra were recorded on a high-resolution LTQ Orbitrap Elite™ mass spectrometer (Thermo Fisher Scientific, Waltham, MA, USA). The solutions were infused into the ESI source at a flow rate of 5.00 μL/min. Spectra were recorded with a resolution of 120,000 (FWHM). Instrument conditions were as follows: spray voltage 3500 V, capillary temperature 275 °C, sheath gas 12 (arbitrary units),

auxiliary gas 3 (arbitrary units), sweep gas 0 (arbitrary units), probe heater temperature 50 °C. Yields refer to pure isolated materials after filtration only (No column chromatography).

Instrumental details of Microwave

Model: DISCOVER SP
SERIAL NO: DC8609
MODEL NO: 909155
VOLTS: 180/240 VAC
MAX. MICROWAVE POWER: 300 W
MAX. CUR: 6.3 A
MAX PWR: 1100
FREQ: 50/60 Hz.

Pictorial diagram of Microwave instrument

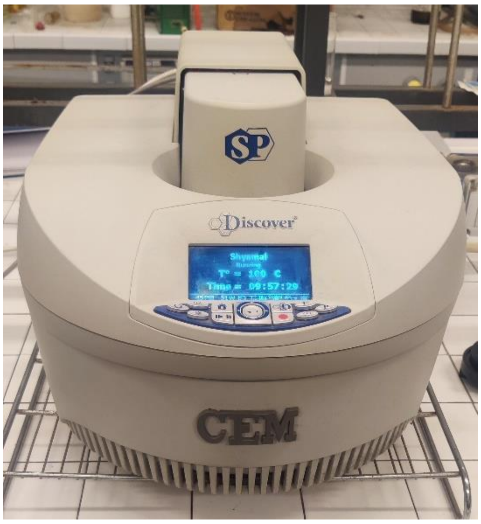

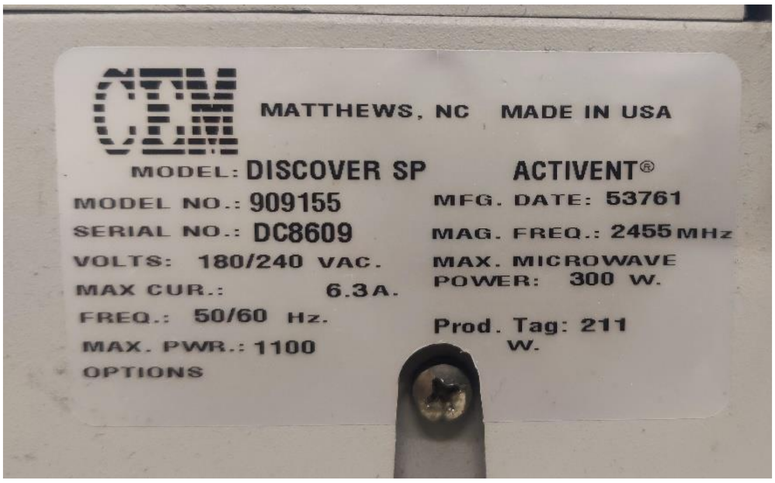

FTIR Instrumental details

Jasco FTIR-4X

MODEL: PKS-D1

SERIAL#: C149061818

POWER: AC V

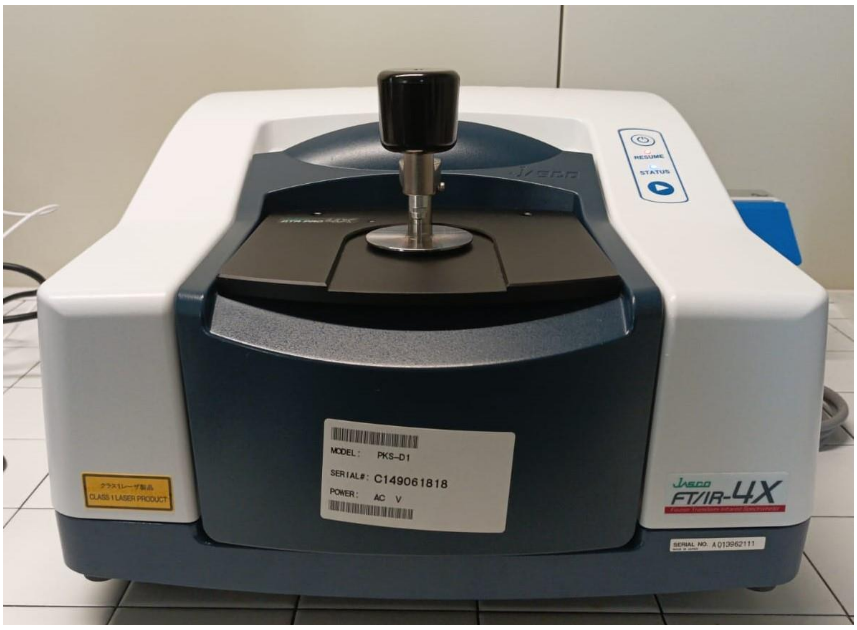

Procedure A. GENERAL PROCEDURE FOR THE SYNTHESIS OF 2-PHENYLQUINAZOLIN-4(3*H*)-ONE

Anthranilamide (0.5 mmol) and sodium hydroxy(phenyl)methanesulfonate (**2a**) (0.5 mmol) were placed in a 10 mL microwave vial equipped with a stir bar. After addition of 2 mL of water the vial was capped properly, then the reaction mixture underwent microwave irradiation at 100 °C with stirring for 10 hours. The resulting solid crude product was then filtered and washed with water to yield 2-phenylquinazolin-4(3*H*)-one (**3aa**). In certain instances, the crude reaction mixture was quenched by pouring it into ice water to enhance product yield.

LIST OF BERTAGNINI'S SALTS:

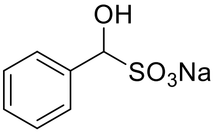

2a

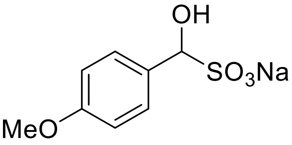

2b

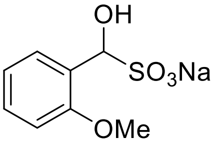

2c

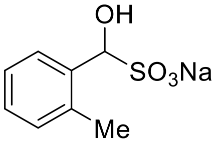

2d

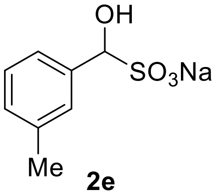

2e

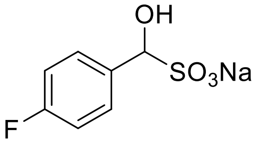

2f

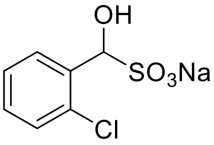

2g

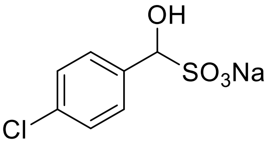

2h

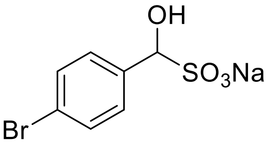

2i

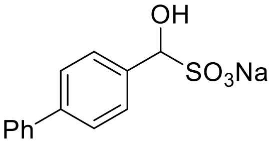

2j

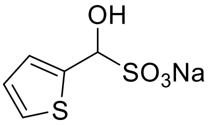

2k

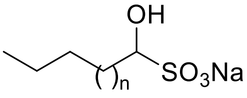

n = X

2l

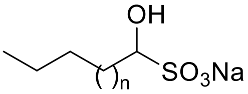

n = X

2m

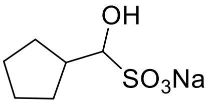

2n

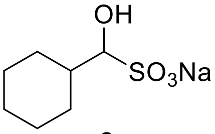

2o

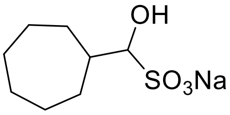

2p

LIST OF ANTHRANILAMIDES:

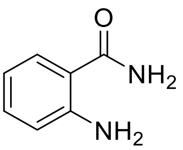

1a

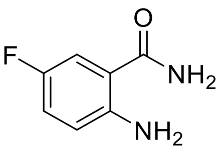

1b

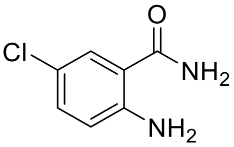

1c

Table S1. Calculation of Ecoscale score[a]

The Eco-scale Score for the microwave asssited synthesis of 2-Phenylquinazolin-4(3*H*)-one (**3aa)**

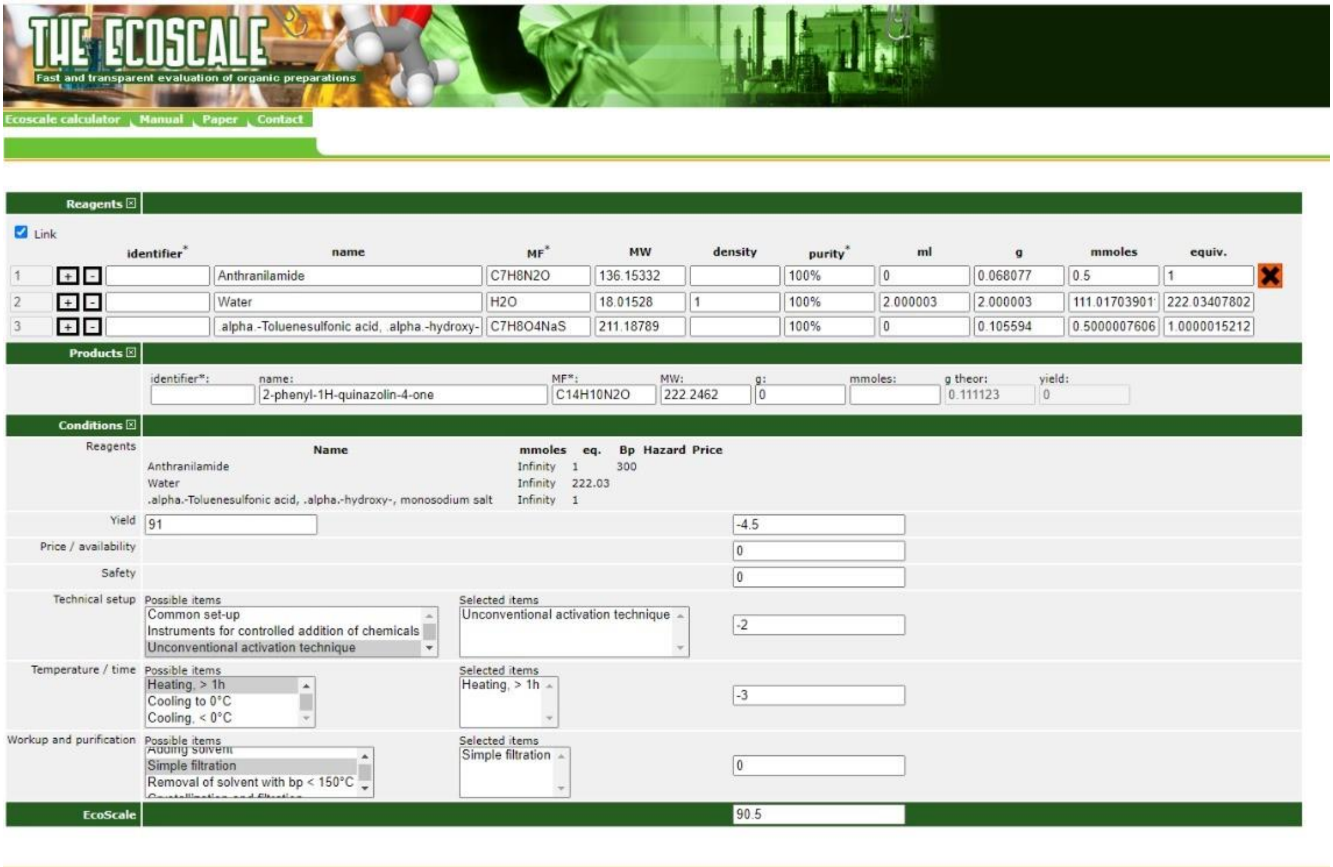

Entry	Parameters	Penalty Points
1	Yield (91%)	-4.5
2	Price/availability	0
3	Safety	0
4	Technical set-up (Common set-up)	-2
5	Temperature/time (r.t.; < 24 h)	-3
6	Work-up and purification (Adding solvent, Simple filtration, removal of solvent with bp <150 °C)	0
	**EcoScale Score**	90.5

[a] Values calculated using the eco scale calculator software available at the link: http://ecoscale.cheminfo.org/calculator

The Eco-scale Score for the reported synthesis of 2-Phenylquinazolin-4(3*H*)-one (**3aa)**

Yang, X.; Cheng, G.; Shen, J.; Kuai, C.; Cui, X,. *Org. Chem. Front.* **2015,** *2*, 366-368.

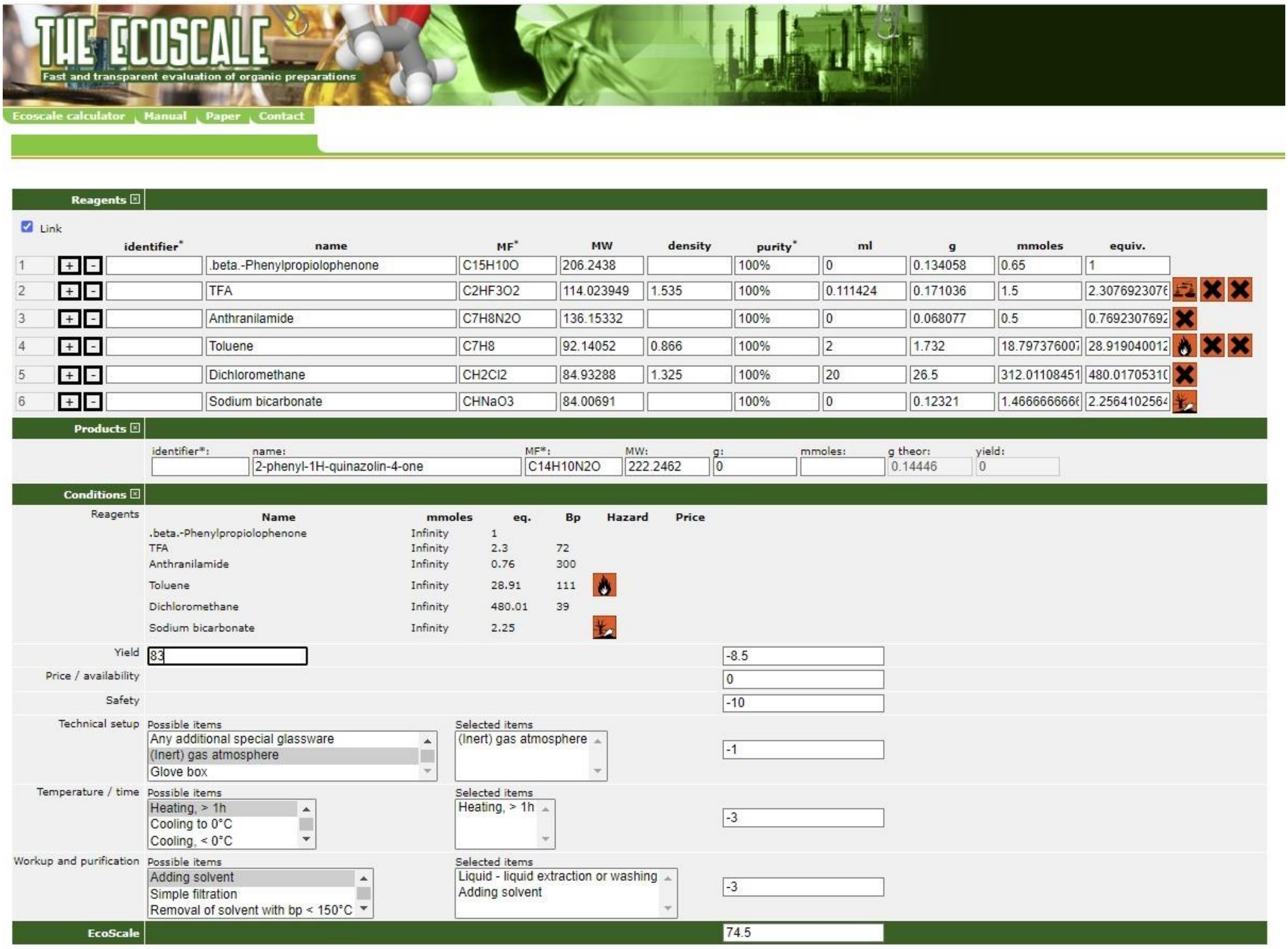

Entry	Parameters	Penalty Points
1	Yield (83%)	-8.5
2	Price/availability	0
3	Safety	-10
4	Technical set-up (Common set-up)	-1
5	Temperature/time (r.t.; < 24 h)	-3
6	Work-up and purification (Adding solvent, Simple filtration, removal of solvent with bp <150 °C)	-3
	**EcoScale Score**	74.5

[a]Values calculated using the eco scale calculator software available at the link: http://ecoscale.cheminfo.org/calculator

Calculation of the Green Chemistry Metrics for the the microwave asssited synthesis of 2-Phenylquinazolin-4(3*H*)-one (3aa)

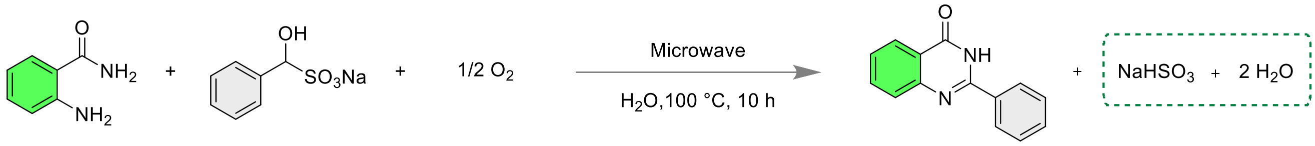

**1a**	**2a**		**3aa**	
Molecular Weight: 136,15	Molecular Weight: 210,18	Molecular Weight: 16,00	Molecular Weight: 222,25	Molecular Weight: 140,08
0.5 mmol	0.5 mmol	0.5 mmol	0.455 mmol	
			91% yield	
**68.07 mg**	**105.1 mg**	**8,00 mg**	**(101,1 mg)**	**70,04 mg**

Solvent: 2000 mg

Atom **E**conomy (***AE***) = $\frac{\text{mass of desired product}}{\text{total mass of reagents}} = \frac{222,25}{136,15 + 210,18 + 16,00} \times 100 = \frac{222,25}{362,33} \times 100 =$ **61,3**

Enviromental factor (***E***) = $\frac{\text{mass of total waste}}{\text{mass of desired product}} = \frac{(70,04 + 2000)\text{ mg}}{101,1\text{ mg}} = \frac{2070.04\text{ mg}}{101,1\text{ mg}} =$ **20,4**

Work-up: H_2O (6000 mg)

E (after purification: filtration and washing = $\frac{(70,04 + 2000 + 6000)\text{ mg}}{101.1\text{ mg}} = \frac{8070,04\text{ mg}}{101.1\text{ mg}} =$ **79,8**

Reaction **M**ass **E**ffinciecy (***RMS***)= $\frac{\text{actual mass of desired product}}{\text{total mass of reagents}} = \frac{101,1\text{ mg}}{(68,07 + 105,1 + 8,00)\text{ mg}} \times 100 = \frac{101,10\text{ mg}}{181,17\text{ mg}} \times 100 =$ **55,8**

Calculation of the Green Chemistry Metrics for the reported synthesis of 2-Phenylquinazolin-4(3*H*)-one (3aa)

Yang, X.; Cheng, G.; Shen, J.; Kuai, C.; Cui, X,. *Org. Chem. Front.* **2015,** *2*, 366-368.

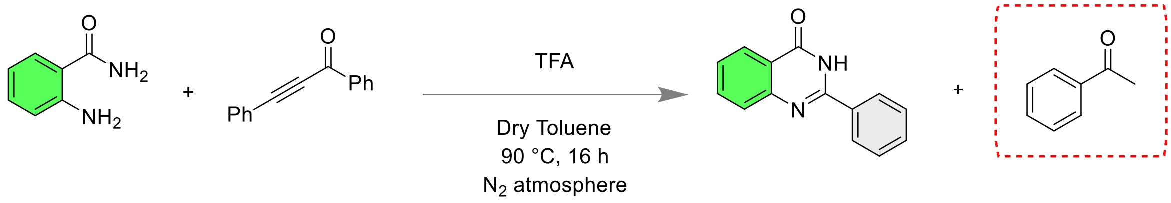

Molecular Weight: 136,15	Molecular Weight: 206,24	Molecular Weight: 114.02	Molecular Weight: 222,25	Molecular Weight: 120,15
0.5 mmol	0.65 mmol	1.5 mmol	0.415	
			83% yield	
**68.07 mg**	**134.1 mg**	**171.03 mg**	**(92.23 mg)**	**60.08 mg**

Excess Reagent **: 30,94 mg + 171,03 mg**

Solvent: **1734,00 mg**

Atom **E**conomy (***AE***) = $\frac{\textit{mass of desired product}}{\textit{total mass of reagents}} = \frac{222,25}{136,15 + 206,24} \text{ X } 100 = \frac{222,25}{342,39} \text{ X } 100$ = **64,9**

Enviromental factor (***E***) = $\frac{\textit{mass of total waste}}{\textit{mass of desired product}} = \frac{(30,94 + 171,03 + 60,08 + 1734,00) \text{ mg}}{92,23 \text{ mg}} = \frac{1996,05 \text{ mg}}{92,23 \text{ mg}}$ = **21,6**

Work-up: DCM (300 mL, 399000,00 mg, H_2O (30 mL, 30000,00 mg), $NaHCO_3$ 126,01 mg + EtOH (1000,00 mg)

E (after purification: filtration and washing = $\frac{(30,94 + 171,03 + 60,08 + 1734,00 + 399000,00 + 30000,00 + 126,01 + 1000,00) \text{ mg}}{92.23 \text{ mg}} = \frac{432131,06 \text{ mg}}{92.23 \text{ mg}}$ = **4684,61**

Reaction **M**ass **E**ffinciecy (***RMS***)= $\frac{\textit{actual mass of desired product}}{\textit{total mass of reagents}} = \frac{92,23 \text{ mg}}{(68,07 + 134,10) \text{ mg}} \text{ X } 100 = \frac{92,23 \text{ mg}}{202,17 \text{ mg}} \text{ X } 100$ = **45,6**

CHARACTERIZATION DATA OF THE SYNTHESIZED COMPOUNDS

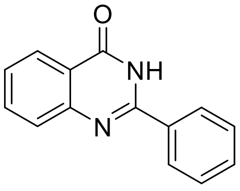

2-Phenylquinazolin-4(3*H*)-one (3aa):[1] The title compound was synthesized according to the general procedure **A**; White solid; Yield: 91% (101 mg); ^{1}H NMR (600 MHz, DMSO-*d*$_6$) δ 12.53 (s, 1H), 8.20 – 8.13 (m, 3H), 7.82 (t, *J* = 7.2 Hz, 1H), 7.74 (d, *J* = 8.4 Hz, 1H), 7.60 – 7.48 (m, 4H); ^{13}C NMR (151 MHz, DMSO-*d*$_6$) δ 162.3, 152.3, 148.8, 134.6, 132.7, 131.4, 128.6, 127.8, 127.5, 126.6, 125.9, 121.0.

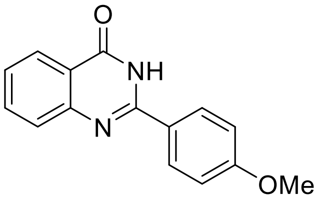

2-(4-Methoxyphenyl)quinazolin-4(3*H*)-one (3ab):[2] The title compound was synthesized according to the general procedure **A**; White solid; Yield: 78% (98.5 mg); ^{1}H NMR (600 MHz, DMSO-*d*$_6$) δ 12.39 (s, 1H), 8.19 (d, *J* = 5.4 Hz, 2H), 8.13 (s, 1H), 7.80 (s, 1H), 7.70 (s, 1H), 7.47 (s, 1H), 7.08 (s, 2H), 3.84 (s, 3H); ^{13}C NMR (151 MHz, DMSO-*d*$_6$) δ 162.3, 161.9, 151.8, 148.9, 134.5, 129.4, 127.3, 126.1, 125.8, 124.8, 120.7, 113.9, 55.4.

2-(2-Methoxyphenyl)quinazolin-4(3*H*)-one (3ac):[3] The title compound was synthesized according to the general procedure **A**; White solid; Yield: 69% (87 mg); ^{1}H NMR (600 MHz, DMSO-*d*$_6$) δ 12.08 (s, 1H), 8.15 (dd, *J* = 7.8, 1.2 Hz, 1H), 7.85 – 7.79 (m, 1H), 7.71 (dd, *J* = 12.0, 4.8 Hz, 2H), 7.56 – 7.51 (m, 2H), 7.19 (d, *J* = 8.4 Hz, 1H), 7.10 (t, *J* = 7.2 Hz, 1H), 3.86 (s, 3H); ^{13}C NMR (151 MHz, DMSO-*d*$_6$) δ 161.3, 157.2, 152.4, 148.9, 134.4, 132.2, 130.5, 127.3, 126.6, 125.8, 122.6, 120.9, 120.5, 111.9, 55.8.

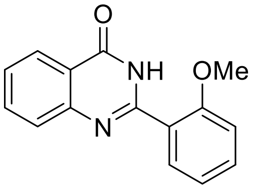

2-(o-Tolyl)quinazolin-4(3*H*)-one (**3ad**)[4]: The title compound was synthesized according to the general procedure **A**; White solid; Yield: 65% (77 mg); ^{1}H NMR (600 MHz, DMSO-d_6) δ 12.43 (s, 1H), 8.17 (d, *J* = 7.8 Hz, 1H), 7.83 (t, *J* = 7.2 Hz, 1H), 7.69 (d, *J* = 7.8 Hz, 1H), 7.54 (d, *J* = 7.2 Hz, 1H), 7.53 – 7.49 (m, 1H), 7.43 (t, *J* = 7.2 Hz, 1H), 7.36 – 7.30 (m, 2H), 2.39 (s, 3H); ^{13}C NMR (151 MHz, DMSO-d_6) δ 161.8, 154.4, 148.7, 136.1, 134.4, 134.2, 130.5, 129.9, 129.1, 127.4, 126.6, 125.8, 125.7, 120.9, 19.5.

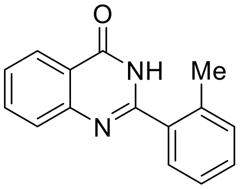

2-(m-Tolyl)quinazolin-4(3*H*)-one (3ae):[5] The title compound was synthesized according to the general procedure **A**; White solid; Yield: 74% (88 mg); ^{1}H NMR (600 MHz, DMSO-d_6) δ 12.45 (s, 1H), 8.15 (d, *J* = 7.2 Hz, 1H), 8.02 (s, 1H), 7.97 (d, *J* = 7.8 Hz, 1H), 7.85 – 7.81 (m, 1H), 7.74 (d, *J* = 7.8 Hz, 1H), 7.54 – 7.49 (m, 1H), 7.45 – 7.37 (m, 2H), 2.40 (s, 3H); ^{13}C NMR (151 MHz, DMSO-d_6) δ 162.2, 152.4, 148.8, 137.9, 134.6, 132.7, 132.0, 128.5, 128.3, 127.5, 126.5, 125.9, 124.9, 120.9, 20.9.

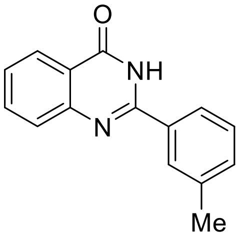

2-(4-Fluorophenyl)quinazolin-4(3*H*)-one (3af):[6] The title compound was synthesized according to the general procedure **A**; White solid; Yield: 83% (99.5 mg); ^{1}H NMR (600 MHz, DMSO-d_6) δ 12.55 (s, 1H), 8.27 – 8.22 (m, 2H), 8.15 (d, *J* = 7.8 Hz, 1H), 7.82 (t, *J* = 7.2 Hz, 1H), 7.72 (d, *J* = 8.4 Hz, 1H), 7.51 (t, *J* = 7.2 Hz, 1H), 7.38 (t, *J* = 8.4 Hz, 2H); ^{13}C NMR (151 MHz, DMSO-d_6) δ 164.1 (d, $^1J_{C\text{-}F}$ = 249.4 Hz), 162.2, 151.4, 148.7, 134.6, 130.4 (d, $^3J_{C\text{-}F}$ = 9.0 Hz), 129.2 (d, $^4J_{C\text{-}F}$ = 2.5 Hz), 127.5, 126.6, 125.9, 120.9, 115.6 (d, $^2J_{C\text{-}F}$ = 21.9 Hz).

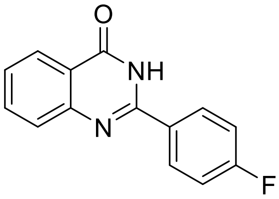

2-(2-Chlorophenyl)quinazolin-4(3*H*)-one (**3ag**):[7] The title compound was synthesized according

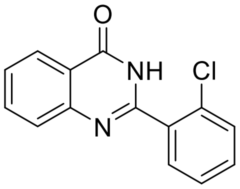

to the general procedure **A**; White solid; Yield: 63% (81 mg); ^{1}H NMR (600 MHz, DMSO-d_6) δ 12.65 (s, 1H), 8.18 (dd, *J* = 7.8, 1.2 Hz, 1H), 7.88 – 7.82 (m, 1H), 7.71 (d, *J* = 7.8 Hz, 1H), 7.67 (dd, *J* = 7.8, 1.2 Hz, 1H), 7.63 – 7.60 (m, 1H), 7.58 – 7.55 (m, 2H), 7.50 (td, *J* = 7.8, 1.2 Hz, 1H); ^{13}C NMR (151 MHz, DMSO-d_6) δ 161.5, 152.3, 148.5, 134.6, 133.8, 131.6, 131.5, 130.9, 129.6, 127.4, 127.2, 127.1, 125.9, 121.2.

2-(4-Bromophenyl)quinazolin-4(3*H*)-one (3ai):[1] The reaction was performed in DMSO solvent

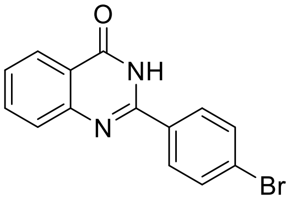

and reaction mixture was poured into water to get the solid precipitate. Further crude product was washed with excess water to obtain the pure product. White solid; Yield: 86% (129 mg); ^{1}H NMR (600 MHz, DMSO-d_6) δ 12.60 (s, 1H), 8.15 (dd, *J* = 7.8, 1.2 Hz, 1H), 8.13 (s, 1H), 8.11 (s, 1H), 7.86 – 7.81 (m, 1H), 7.76 (d, *J* = 1.8 Hz, 1H), 7.75 (d, *J* = 2.4 Hz, 1H), 7.73 (s, 1H), 7.55 – 7.51 (m, 1H); ^{13}C NMR (151 MHz, DMSO-d_6) δ 162.2, 151.5, 148.5, 134.7, 131.9, 131.6, 129.8, 127.4, 126.8, 125.9, 125.2, 120.9.

2-([1,1'-Biphenyl]-4-yl)quinazolin-4(3*H*)-one (**3aj**):[5] The title compound was synthesized

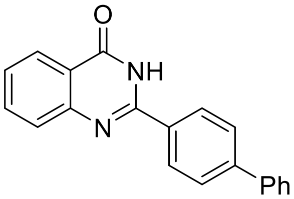

according to the general procedure **A**; White solid; Yield: 81% (120.5 mg); ^{1}H NMR (600 MHz, DMSO-d_6) δ 12.58 (s, 1H), 8.30 (d, *J* = 7.8 Hz, 2H), 8.17 (d, *J* = 7.8 Hz, 1H), 7.84 (t, *J* = 9.0 Hz, 3H), 7.76 (d, *J* = 3.6 Hz, 3H), 7.56 – 7.47 (m, 3H), 7.42 (t, *J* = 7.2 Hz, 1H); ^{13}C NMR (151 MHz, DMSO-d_6) δ 162.3, 151.9, 148.7, 142.9, 138.9, 134.6, 131.5, 129.1, 128.4, 128.2, 127.4, 126.8, 126.7, 126.6, 125.9, 120.9.

2-(Thiophen-2-yl)quinazolin-4(3*H*)-one (3ak):[8] The title compound was synthesized according to

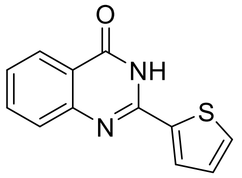

the general procedure **A**; White solid; Yield: 67% (76 mg); ^{1}H NMR (600 MHz, DMSO-d_6) δ 12.48 (s, 1H), 8.12 (d, *J* = 7.8 Hz, 1H), 7.99 (s, 1H), 7.80 (t, *J* = 7.6 Hz, 1H), 7.68 (d, *J* = 8.1 Hz, 1H), 7.63 (d, *J* = 2.7 Hz, 1H), 7.48 (t, *J* = 7.5 Hz, 1H), 6.74 (s, 1H); ^{13}C NMR (151 MHz, DMSO-d_6) δ 161.6, 148.7, 146.6, 146.1, 144.0, 134.6, 127.2, 126.4, 125.9, 121.2, 114.5, 112.5.

2-Hexylquinazolin-4(3*H*)-one (3al):[9] The title compound was synthesized according to the general procedure **A**; White solid; Yield: 73% (84 mg); ^{1}H NMR (600 MHz, DMSO-d_6) δ 12.14 (s, 1H), 8.07

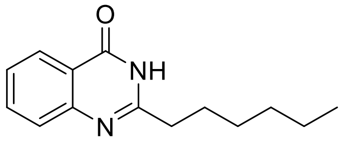

(dd, *J* = 7.8, 1.2 Hz, 1H), 7.77 – 7.72 (m, 1H), 7.58 (d, *J* = 7.8 Hz, 1H), 7.46 – 7.41 (m, 1H), 2.60 – 2.55 (m, 2H), 1.73 – 1.66 (m, 2H), 1.31 – 1.26 (m, 2H), 1.27 – 1.19 (m, 4H), 0.86 – 0.79 (m, 3H); ^{13}C NMR (151 MHz, DMSO-d_6) δ 161.9, 157.6, 149.0, 134.3, 126.8, 125.9, 125.7, 120.8, 34.6, 30.9, 28.2, 26.8, 21.9, 13.9.

2-Decylquinazolin-4(3*H*)-one (3am):[10] The title compound was synthesized according to the

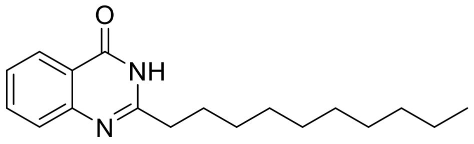

general procedure **A**; White solid; Yield: 68% (97 mg); ^{1}H NMR (600 MHz, DMSO-d_6) δ 12.13 (s, 1H), 8.07 (dd, *J* = 7.8, 1.2 Hz, 1H), 7.78 – 7.73 (m, 1H), 7.57 (d, *J* = 7.8 Hz, 1H), 7.46 – 7.41 (m, 1H), 2.61 – 2.54 (m, 2H), 1.73 – 1.66 (m, 2H), 1.33 – 1.25 (m, 5H), 1.25 – 1.14 (m, 9H), 0.84 – 0.81 (m, 3H); ^{13}C NMR (151 MHz, DMSO-d_6) δ 161.9, 157.6, 148.9, 134.3, 126.8, 125.9, 125.7, 120.8, 34.5, 31.3, 28.9, 28.9, 28.7, 28.5, 26.8, 22.1, 13.9.

6-Fluoro-2-phenylquinazolin-4(3*H*)-one (3ba):[11] The title compound was synthesized according to

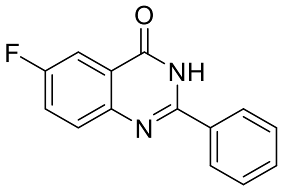

the general procedure **A**; White solid; Yield: 89% (107 mg); 89%; ^{1}H NMR (600 MHz, DMSO-d_6) δ 12.65 (s, 1H), 8.17 (s, 1H), 8.16 – 8.15 (m, 1H), 7.84 – 7.82 (m, 1H), 7.81 (d, *J* = 5.6 Hz, 1H), 7.72 (td, *J* = 8.4, 3.0 Hz, 1H), 7.59 (d, *J* = 7.2 Hz, 1H), 7.56 – 7.53 (m, 2H); ^{13}C NMR (151 MHz, DMSO-d_6) δ 161.6, 159.9 (d, $^1J_{C\text{-}F}$ = 245.5 Hz), 151.8, 145.6, 132.6, 131.4, 130.3 (d, $^3J_{C\text{-}F}$ = 8.3 Hz), 128.6, 127.7, 123.1 (d, $^2J_{C\text{-}F}$ = 24.1 Hz), 122.2 (d, $^3J_{C\text{-}F}$ = 8.6 Hz), 110.5 (d, $^2J_{C\text{-}F}$ = 23.3 Hz).

6-Fluoro-2-(4-methoxyphenyl)quinazolin-4(3*H*)-one (3bb):[12] The title compound was synthesized

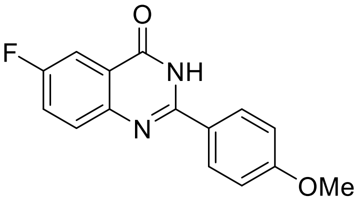

according to the general procedure **A**; White solid; Yield: 82% (111 mg); ^{1}H NMR (600 MHz, DMSO-d_6) δ 12.51 (s, 1H), 8.17 (s, 2H), 7.83 - 7.66 (m, 3H), 7.09 (s, 2H), 3.85 (s, 3H); ^{13}C NMR (151 MHz, DMSO-d_6) δ 161.9, 159.7 (d, $^1J_{C\text{-}F}$ = 245.7 Hz), 151.5, 145.8, 130.0, 129.4, 124.6, 123.1, 122.9, 121.8 (d, $^3J_{C\text{-}F}$ = 10.6 Hz), 114.0, 110.4 (d, $^2J_{C\text{-}F}$ = 23.4 Hz), 55.5.

6-Fluoro-2-(2-methoxyphenyl)quinazolin-4(3*H*)-one (**3bc**):[12] The title compound was synthesized according to the general procedure **A**; White solid; Yield: 78% (106 mg); ^{1}H NMR (600 MHz, DMSO-

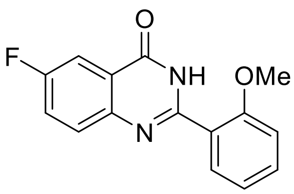

d_6) δ 12.22 (s, 1H), 7.82 (dd, *J* = 8.4, 3.0 Hz, 1H), 7.79 – 7.76 (m, 1H), 7.73 – 7.70 (m, 1H), 7.69 – 7.67 (m, 1H), 7.56 – 7.52 (m, 1H), 7.19 (d, *J* = 8.4 Hz, 1H), 7.09 (t, *J* = 7.2 Hz, 1H), 3.86 (s, 3H); ^{13}C NMR (151 MHz, DMSO-d_6) δ 160.7, 159.9 (d, $^1J_{C\text{-}F}$ = 245.3 Hz), 157.1, 151.9, 145.9, 132.3, 130.4, 130.2 (d, $^3J_{C\text{-}F}$ = 8.3 Hz), 122.9 (d, $^2J_{C\text{-}F}$ = 24.0 Hz), 122.5, 122.2 (d, $^3J_{C\text{-}F}$ = 8.4 Hz), 120.4, 111.9, 110.4 (d, $^2J_{C\text{-}F}$ = 23.3 Hz), 55.8.

6-Fluoro-2-(m-tolyl)quinazolin-4(3*H*)-one (**3be**)**:** The title compound was synthesized according to the general procedure **A**; White solid; Yield: 87% (110 mg); ^{1}H NMR (600 MHz, DMSO-d_6) δ 12.58 (s, 1H), 8.00 (s, 1H), 7.95 (d, *J* = 6.6 Hz, 1H), 7.81 (d, *J* = 6.0 Hz, 2H), 7.72 (d, *J* = 6.0 Hz, 1H), 7.46 – 7.37 (m, 2H), 2.40 (s, 3H); ^{13}C NMR (151 MHz, DMSO-d_6) δ 161.7, 159.9 (d, $^1J_{C\text{-}F}$ = 244.8 Hz), 151.9, 145.6, 137.9, 132.5, 132.0, 130.3, 130.2 (d, $^4J_{C\text{-}F}$ = 4.9 Hz), 128.4 (d, $^3J_{C\text{-}F}$ = 7.9 Hz), 124.9, 123.1 (d, $^2J_{C\text{-}F}$ = 24.2 Hz), 122.2 (d, $^3J_{C\text{-}F}$ = 8.7 Hz), 110.5 (d, $^2J_{C\text{-}F}$ = 23.2 Hz); FTIR $\tilde{\nu}$ max = 3116, 3075, 1675, 1571, 1484, 1294, 879 cm^{-1}; HRMS: calculated for $C_{15}H_{12}FN_2O$: 255.0928 [M+H]$^+$; found: 255.0939.

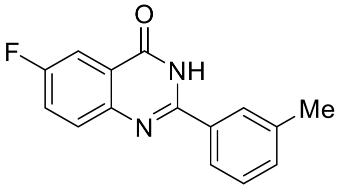

2-(2-Chlorophenyl)-6-fluoroquinazolin-4(3*H*)-one (3bg): The title compound was synthesized according to the general procedure **A**; White solid; Yield: 67% (92 mg); ^{1}H NMR (600 MHz, DMSO-d_6) δ 12.76 (s, 1H), 7.85 (dd, *J* = 8.4, 3.0 Hz, 1H), 7.80 (dd, *J* = 8.4, 4.8 Hz, 1H), 7.74 (td, *J* = 8.4, 3.0 Hz, 1H), 7.67 (dd, *J* = 7.8, 1.8 Hz, 1H), 7.63 – 7.60 (m, 1H), 7.57 (td, *J* = 7.8, 1.8 Hz, 1H), 7.50 (td, *J* = 7.8, 1.2 Hz, 1H); ^{13}C NMR (151 MHz, DMSO-d_6) δ 160.9, 160.3 (d, $^1J_{C\text{-}F}$ = 246.0 Hz), 151.8, 145.4, 133.6, 131.7, 131.5, 130.9, 130.4 (d, $^3J_{C\text{-}F}$ = 10.0 Hz), 129.6, 127.3, 123.1 (d, $^2J_{C\text{-}F}$ = 24.1 Hz), 122.5 (d, $^3J_{C\text{-}F}$ = 8.4 Hz), 110.6 (d, $^2J_{C\text{-}F}$ = 23.3 Hz); FTIR $\tilde{\nu}$ max = 3045, 2977, 1679, 1604, 1481, 927, 763 cm^{-1}; HRMS: calculated for $C_{14}H_9ClFN_2O$: 275.0387 [M+H]$^+$; found: 275.0396.

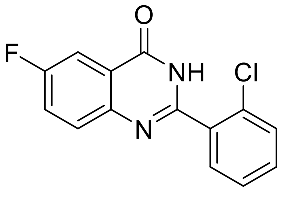

2-([1,1'-Biphenyl]-4-yl)-6-fluoroquinazolin-4(3*H*)-one (**3bj**): The title compound was synthesized

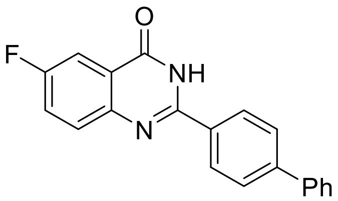

according to the general procedure **A**; White solid; Yield: 58% (92 mg); ^{1}H NMR (600 MHz, DMSO-*d*$_6$) δ 12.70 (s, 1H), 8.29 (s, 2H), 7.86 (s, 4H), 7.78 (s, 3H), 7.55 - 7.49 (m, 3H); ^{13}C NMR (151 MHz, DMSO-*d*$_6$) δ 161.7, 159.9 (d, J = 246.1 Hz), 157.4, 154.1, 151.5, 145.7, 142.9, 138.9, 130.3, 129.1, 128.4, 128.2, 126.8 (d, $^3J_{C\text{-}F}$ = 13.3 Hz), 123.1 (d, $^2J_{C\text{-}F}$ = 23.5 Hz), 122.2, 110.5 (d, $^2J_{C\text{-}F}$ = 22.4 Hz); FTIR $\tilde{\nu}$ max = 3029, 2952, 1660, 1596, 1481, 1301, 836 cm^{-1}; HRMS: calculated for $C_{20}H_{14}FN_2O$: 317.1090 [M+H]$^+$; found: 317.1103.

6-Chloro-2-phenylquinazolin-4(3*H*)-one (**3ca**):[1] The title compound was synthesized according to

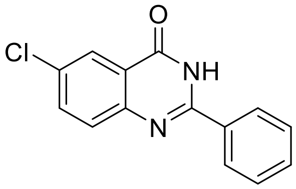

the general procedure **A**; White solid; Yield: 69% (89 mg); ^{1}H NMR (600 MHz, DMSO-d$_6$) δ 12.71 (s, 1H), 8.18 (s, 2H), 8.09 (s, 1H), 7.86 (s, 1H), 7.77 (s, 1H), 7.60 (s, 1H), 7.56 (s, 2H); ^{13}C NMR (151 MHz, DMSO-*d*$_6$) δ 161.3, 152.9, 147.5, 134.7, 132.4, 131.6, 130.8, 129.7, 128.6, 127.8, 124.9, 122.2.

6-Chloro-2-(4-methoxyphenyl)quinazolin-4(3*H*)-one (3cb):[13] The title compound was synthesized

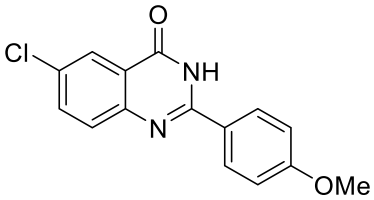

according to the general procedure **A**; White solid; Yield: 58% (83 mg); ^{1}H NMR (600 MHz, DMSO-*d*$_6$) δ 12.56 (s, 1H), 8.19 (s, 1H), 8.17 (s, 1H), 8.06 (d, J = 2.4 Hz, 1H), 7.83 (dd, J = 8.4, 2.4 Hz, 1H), 7.72 (d, J = 8.4 Hz, 1H), 7.09 (d, J = 8.8 Hz, 2H), 3.85 (s, 3H); ^{13}C NMR (151 MHz, DMSO-*d*$_6$) δ 162.0, 161.3, 152.4, 147.7, 134.6, 130.2, 129.6, 129.5, 124.8, 124.5, 121.9, 114.0, 55.5.

6-Chloro-2-(2-methoxyphenyl)quinazolin-4(3*H*)-one (**3cc**):[14] The title compound was synthesized according to the general procedure **A**; White solid; Yield: 59% (85 mg); ^{1}H NMR (600 MHz, DMSO-d_6) δ 12.27 (s, 1H), 8.08 (d, *J* = 2.4 Hz, 1H), 7.85 (dd, *J* = 8.4, 2.4 Hz, 1H), 7.73 (d, *J* = 8.4 Hz, 1H), 7.70 (dd, *J* = 7.8, 1.7 Hz, 1H), 7.56 – 7.51 (m, 1H), 7.20 (d, *J* = 8.4 Hz, 1H), 7.09 (t, *J* = 7.8 Hz, 1H), 3.86 (s, 3H); ^{13}C NMR (151 MHz, DMSO-d_6) δ 160.3, 157.1, 152.8, 147.8, 134.5, 132.4, 130.8, 130.5, 129.6, 124.8, 122.4, 122.2, 120.4, 111.9, 55.8.

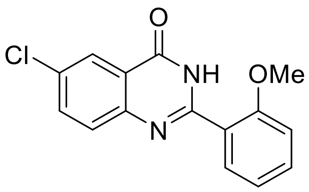

6-Chloro-2-(o-tolyl)quinazolin-4(3*H*)-one (**3cd**):[15] The title compound was synthesized according to the general procedure **A**; White solid; Yield: 53% (72 mg); ^{1}H NMR (600 MHz, DMSO-d_6) δ 12.61 (s, 1H), 8.10 (d, *J* = 2.4 Hz, 1H), 7.86 (dd, *J* = 8.4, 2.4 Hz, 1H), 7.71 (d, *J* = 8.4 Hz, 1H), 7.51 (d, *J* = 7.2 Hz, 1H), 7.44 (t, *J* = 7.2 Hz, 1H), 7.35 (d, *J* = 8.4 Hz, 1H), 7.32 (d, *J* = 7.2 Hz, 1H), 2.38 (s, 3H); ^{13}C NMR (151 MHz, DMSO-d_6) δ 160.8, 154.9, 147.4, 136.2, 134.5, 133.9, 130.8, 130.6, 130.0, 129.6, 129.1, 125.7, 124.8, 122.2, 19.5.

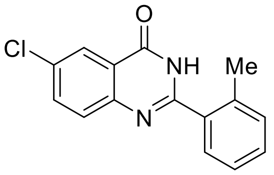

6-Chloro-2-(m-tolyl)quinazolin-4(3*H*)-one (**3ce**): The title compound was synthesized according to the general procedure **A**; White solid; Yield: 56% (76 mg); ^{1}H NMR (600 MHz, DMSO-d_6) δ 12.63 (s, 1H), 8.08 (d, *J* = 2.4 Hz, 1H), 8.01 (s, 1H), 7.96 (d, *J* = 7.2 Hz, 1H), 7.85 (dd, *J* = 8.4, 2.4 Hz, 1H), 7.76 (d, *J* = 8.4 Hz, 1H), 7.45 – 7.40 (m, 2H), 2.41 (s, 3H); ^{13}C NMR (151 MHz, DMSO-d_6) δ 161.3, 152.9, 137.9, 134.8, 134.7, 132.9, 132.4, 132.2, 130.7, 129.7, 128.5, 128.4, 124.9, 124.9, 20.9; FTIR $\tilde{\nu}$ max = 3023, 2925, 1671, 1579, 1467, 1309, 842 cm^{-1}; HRMS: calculated for $C_{15}H_{12}ClN_2O$: 271.0638 [M+H]$^+$; found: 271.0649.

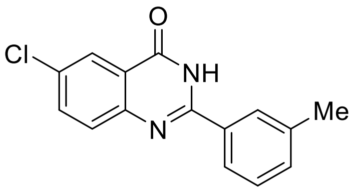

6-Chloro-2-(4-fluorophenyl)quinazolin-4(3*H*)-one (**3cf**):[16] The title compound was synthesized according to the general procedure **A**; White solid; Yield: 65% (89 mg); ^{1}H NMR (600 MHz, DMSO-*d*$_6$) δ 12.73 (s, 1H), 8.26 – 8.21 (m, 2H), 8.08 (d, *J* = 2.4 Hz, 1H), 7.85 (dd, *J* = 8.4, 2.4 Hz, 1H), 7.75 (d, *J* = 8.4 Hz, 1H), 7.39 (t, *J* = 8.4 Hz, 2H); ^{13}C NMR (151 MHz, DMSO-*d*$_6$) δ 164.1 (d, $^{1}J_{C-F}$ = 249.9 Hz), 161.3, 151.9, 147.4, 134.7, 130.8, 130.5 (d, $^{3}J_{C-F}$ = 9.0 Hz), 129.7, 128.9 (d, $^{4}J_{C-F}$ = 2.8 Hz), 124.9, 122.1, 115.7 (d, $^{2}J_{C-F}$ = 22.0 Hz).

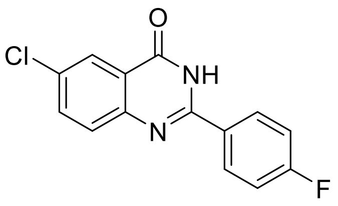

6-Chloro-2-(2-chlorophenyl)quinazolin-4(3*H*)-one (**3cg**):[16] The title compound was synthesized according to the general procedure **A**; White solid; Yield: 61% (89 mg); ^{1}H NMR (600 MHz, DMSO-*d*$_6$) δ 12.82 (s, 1H), 8.12 (d, *J* = 2.4 Hz, 1H), 7.89 (dd, *J* = 8.4, 2.4 Hz, 1H), 7.75 (d, *J* = 8.4 Hz, 1H), 7.67 (dd, *J* = 7.2, 1.2 Hz, 1H), 7.62 (d, *J* = 8.4 Hz, 1H), 7.58 (td, *J* = 7.2, 1.2 Hz, 1H), 7.50 (t, *J* = 7.2 Hz, 1H); ^{13}C NMR (151 MHz, DMSO-*d*$_6$) δ 160.5, 152.7, 147.3, 134.7, 133.5, 131.8, 131.4, 131.4, 130.9, 129.7, 129.6, 127.2, 124.9, 122.5.

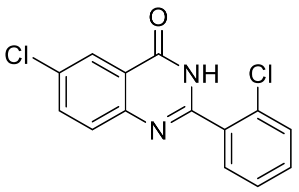

6-Chloro-2-(4-chlorophenyl)quinazolin-4(3*H*)-one (**3ch**):[16] The title compound was synthesized according to the general procedure **A**; White solid; Yield: 64% (93 mg); ^{1}H NMR (600 MHz, DMSO-*d*$_6$) δ 12.67 (s, 1H), 8.20 (d, *J* = 7.8 Hz, 2H), 8.09 (s, 1H), 7.85 (d, *J* = 8.4 Hz, 1H), 7.76 (d, *J* = 8.4 Hz, 1H), 7.62 (d, *J* = 7.8 Hz, 2H); ^{13}C NMR (151 MHz, DMSO-*d*$_6$) δ 161.3, 152.3, 141.4, 139.2, 136.3, 134.4, 129.8, 129.5, 128.5, 127.6, 124.7, 122.2.

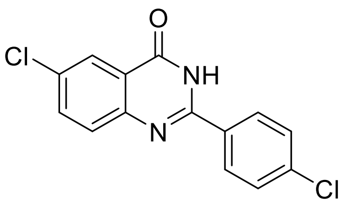

2-(4-Bromophenyl)-6-chloroquinazolin-4(3*H*)-one (3ci):[17] The title compound was synthesized

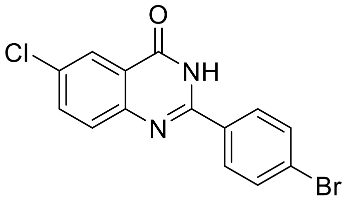

according to the general procedure **A**; White solid; Yield: 76% (127 mg); ^{1}H NMR (600 MHz, DMSO-d_6) δ 12.66 (s, 1H), 8.14 (s, 1H), 8.12 (s, 1H), 8.08 (d, *J* = 2.5 Hz, 1H), 7.84 (dd, *J* = 8.7, 2.5 Hz, 1H), 7.76 (d, *J* = 8.4 Hz, 3H); ^{13}C NMR (151 MHz, DMSO-d_6) δ 161.4, 152.2, 134.4, 131.4, 131.4, 130.6, 129.7, 129.4, 128.8, 125.1, 124.7, 122.2.

1'*H*-spiro[cyclopentane-1,2'-quinazolin]-4'(3'*H*)-one (3an)[18] The title compound was synthesized

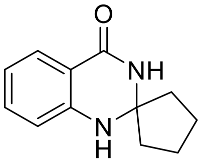

according to the general procedure **A**; White solid; Yield: 56% (56.6 mg); ^{1}H NMR (600 MHz, DMSO-d_6) δ 8.07 (s, 1H), 7.57 (dd, *J* = 7.8, 1.2 Hz, 1H), 7.25 – 7.14 (m, 1H), 6.72 (s, 1H), 6.69 (d, *J* = 7.8 Hz, 1H), 6.63 (t, *J* = 7.8 Hz, 1H), 1.83 – 1.75 (m, 4H), 1.68 – 1.63 (m, 4H); ^{13}C NMR (151 MHz, DMSO-d_6) δ 163.5, 147.5, 133.0, 127.3, 116.6, 114.6, 114.3, 77.1, 39.3, 21.9.

1'*H*-spiro[cyclohexane-1,2'-quinazolin]-4'(3'*H*)-one (3ao):[18] The title compound was synthesized

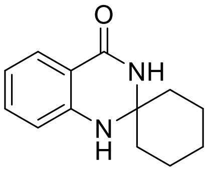

according to the general procedure **A**; White solid; Yield: 73% (79 mg); ^{1}H NMR (600 MHz, DMSO-d_6) δ 7.90 (s, 1H), 7.56 (dd, *J* = 7.8, 1.2 Hz, 1H), 7.24 – 7.16 (m, 1H), 6.80 (d, *J* = 7.8 Hz, 1H), 6.62 (d, *J* = 7.8 Hz, 1H), 6.60 (d, *J* = 5.4 Hz, 1H), 1.78 – 1.67 (m, 2H), 1.65 – 1.58 (m, 2H), 1.58 – 1.51 (m, 4H), 1.46 – 1.36 (m, 1H), 1.29 – 1.20 (m, 1H); ^{13}C NMR (151 MHz, DMSO-d_6) δ 163.2, 146.8, 133.1, 127.1, 116.5, 114.6, 114.5, 67.8, 37.2, 24.6, 20.9.

1'*H*-spiro[cycloheptane-1,2'-quinazolin]-4'(3'*H*)-one (3ap):[18] The reaction was performed in DMSO solvent and reaction mixture was poured into water to get the solid precipitate. Further crude product was washed with excess water to obtaine the pure product. White solid; Yield: 84% (97 mg); ^{1}H NMR (600 MHz, DMSO-d_6) δ 8.00 (s, 1H), 7.55 (dd, *J* = 7.8, 1.2 Hz, 1H), 7.23 – 7.16 (m, 1H), 6.71 (s, 1H), 6.70 (s, 1H), 6.62 – 6.58 (m, 1H), 1.92 – 1.81 (m, 4H), 1.51 (s, 8H); ^{13}C NMR (151 MHz, DMSO-d_6) δ 162.9, 146.7, 133.1, 127.1, 116.3, 114.4, 114.3, 71.9, 41.0, 29.2, 20.9.

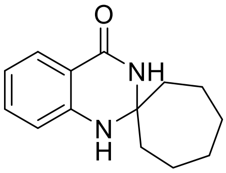

6'-Chloro-1'*H*-spiro[cyclohexane-1,2'-quinazolin]-4'(3'*H*)-one (3co):[18] The title compound was synthesized according to the general procedure **A**; White solid; Yield: 86 % (108 mg); ^{1}H NMR (600 MHz, DMSO-d_6) δ 8.10 (s, 1H), 7.49 (d, *J* = 2.4 Hz, 1H), 7.24 (dd, *J* = 8.4, 2.4 Hz, 1H), 6.87 – 6.78 (m, 2H), 1.78 – 1.70 (m, 2H), 1.63 – 1.57 (m, 2H), 1.57 – 1.50 (m, 4H), 1.47 – 1.38 (m, 1H), 1.27- 1.19 (m, 1H); ^{13}C NMR (151 MHz, DMSO-d_6) δ 162.0, 145.5, 132.9, 126.2, 120.1, 116.6, 115.6, 68.0, 37.1, 24.5, 20.8.

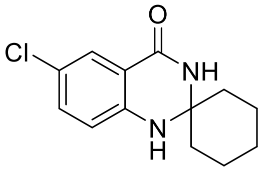

NMR SPECTRA

^{1}H NMR (600 MHz, DMSO-d$_6$)

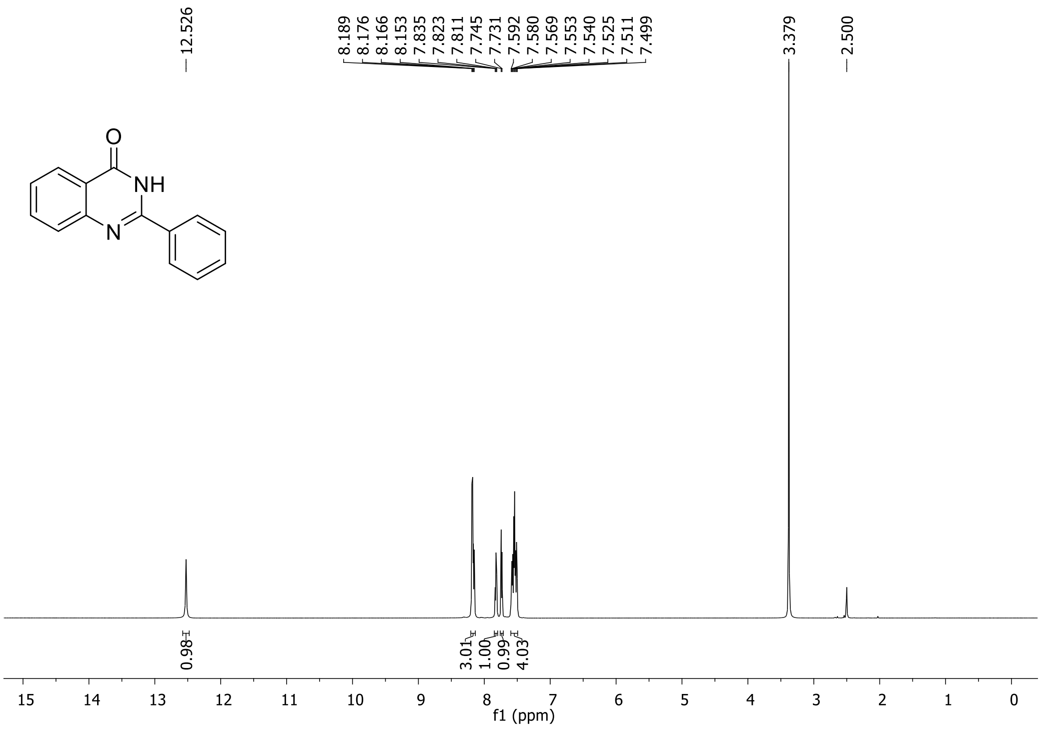

Figure S1. ^{1}H NMR spectrum of 2-Phenylquinazolin-4(3*H*)-one (**3aa**)

^{13}C NMR (151 MHz, DMSO-d$_6$)

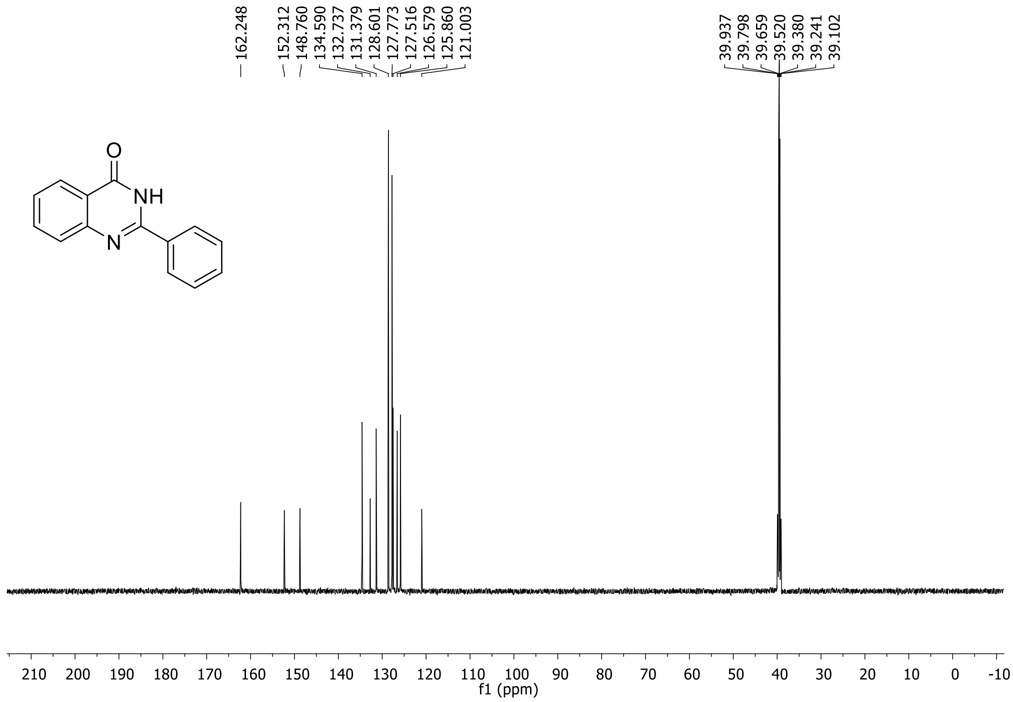

Figure S2. ^{13}C NMR spectrum of 2-Phenylquinazolin-4(3*H*)-one (**3aa**)

^{1}H NMR (600 MHz, DMSO-d$_6$)

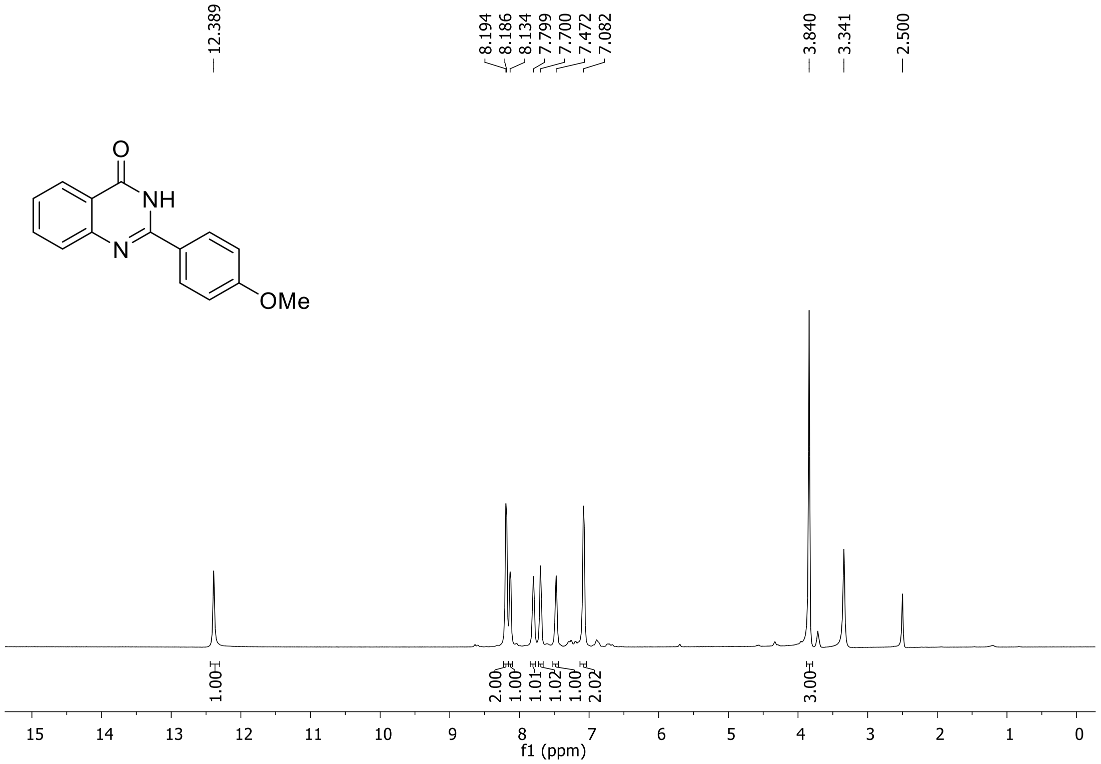

Figure S3. ^{1}H NMR spectrum of 2-(4-Methoxyphenyl)quinazolin-4(3*H*)-one (**3ab**)

^{13}C NMR (151 MHz, DMSO-d$_6$)

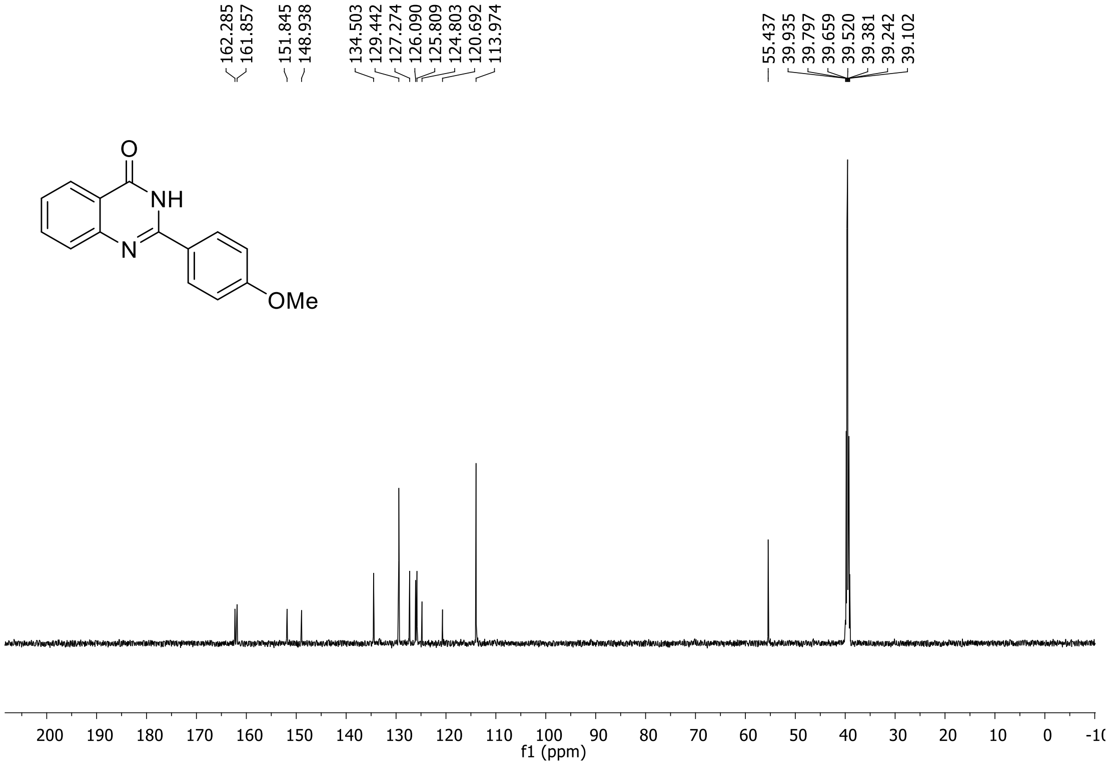

Figure S4. ^{13}C NMR spectrum of 2-(4-Methoxyphenyl)quinazolin-4(3*H*)-one (**3ab**)

^{1}H NMR (600 MHz, DMSO-d_6)

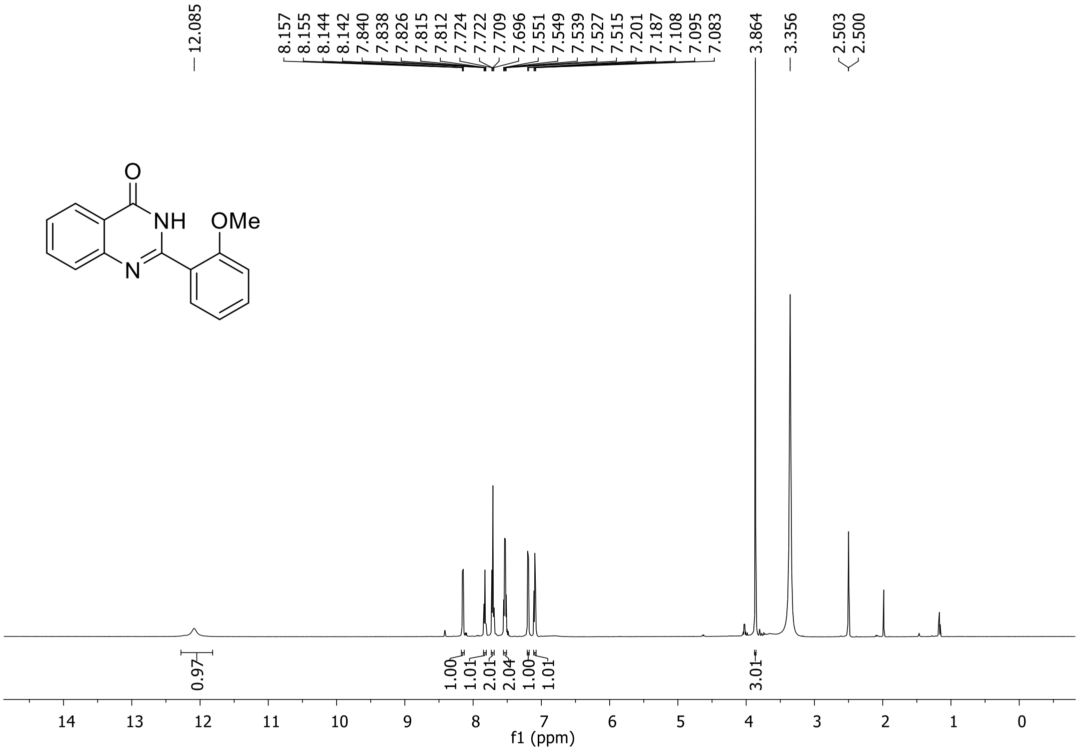

Figure S5. ^{1}H NMR spectrum of 2-(2-Methoxyphenyl)quinazolin-4(3*H*)-one (**3ac**)

^{13}C NMR (151 MHz, DMSO-d_6)

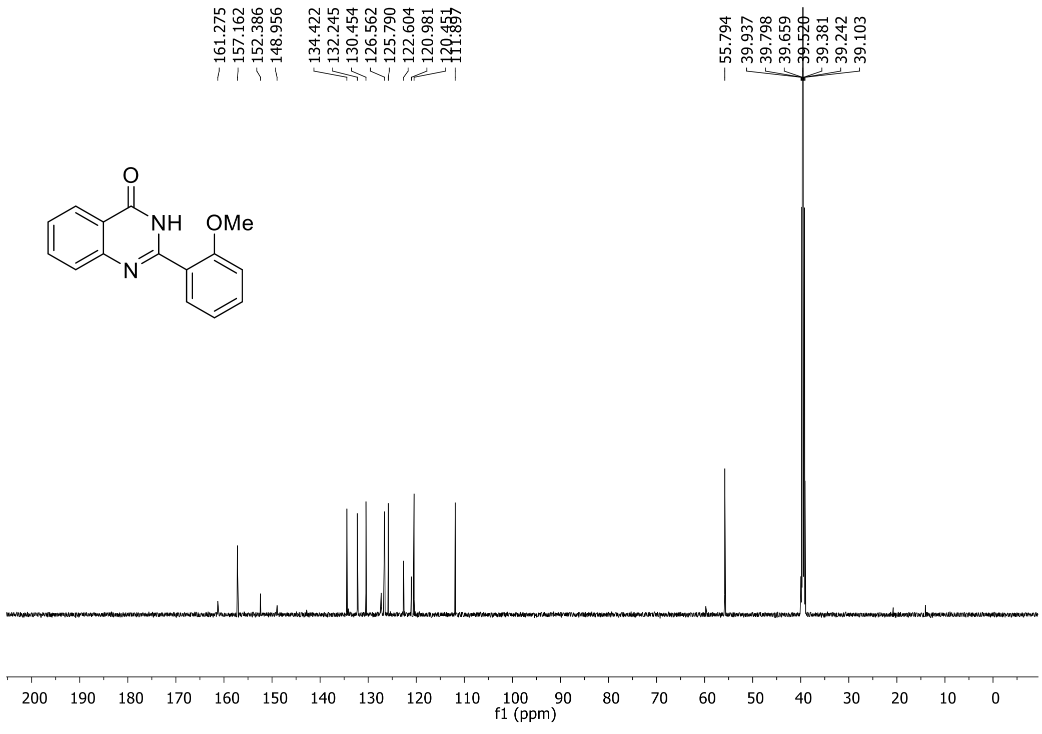

Figure S6. ^{13}C NMR spectrum of 2-(2-Methoxyphenyl)quinazolin-4(3*H*)-one (**3ac**)

^{1}H NMR (600 MHz, DMSO-d$_6$)

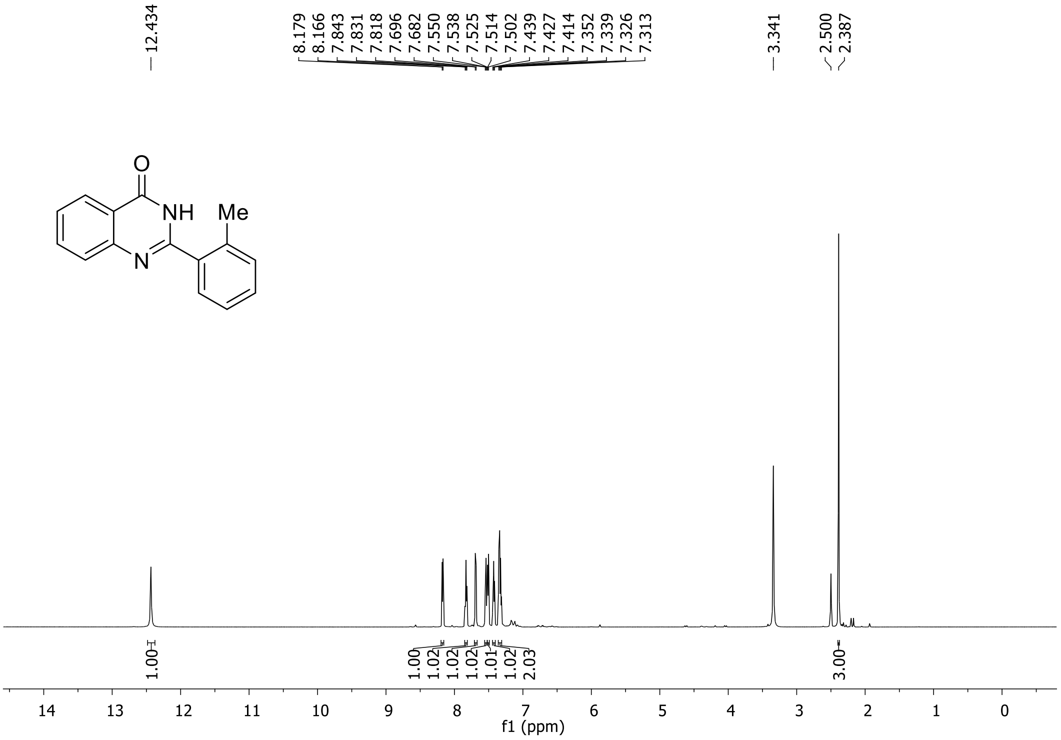

Figure S7. ^{1}H NMR spectrum of 2-(o-Tolyl)quinazolin-4(3*H*)-one (**3ad**)

^{13}C NMR (151 MHz, DMSO-d$_6$)

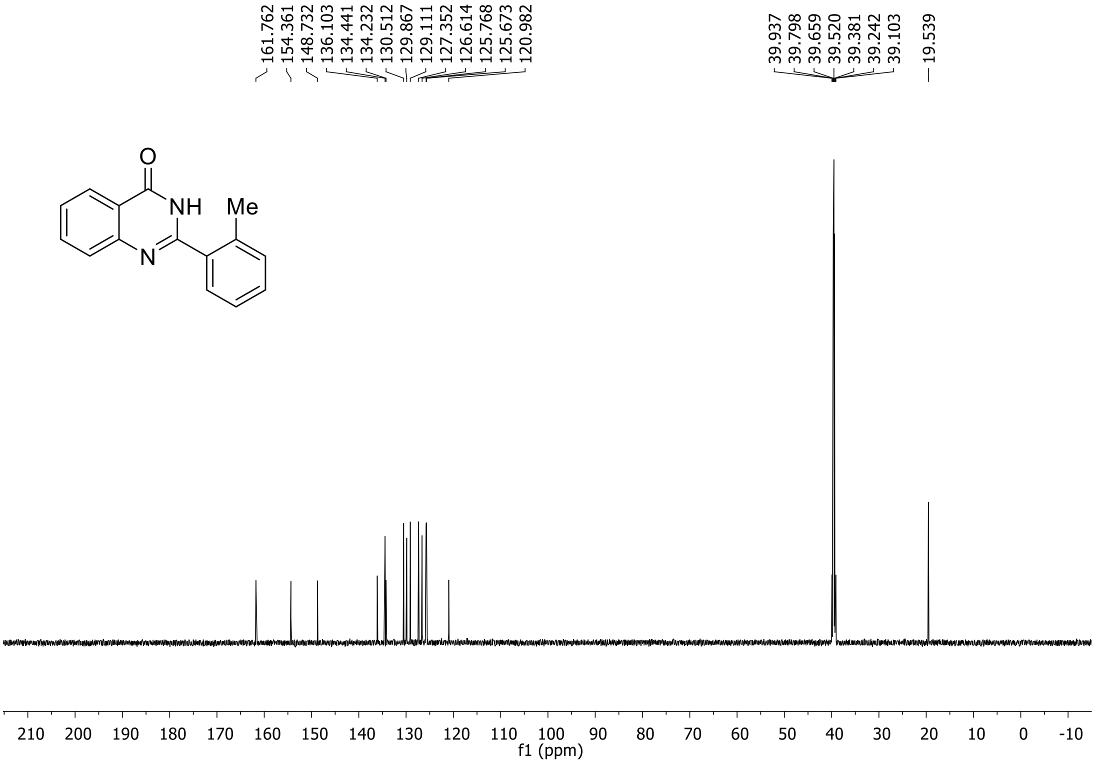

Figure S8. ^{13}C NMR spectrum of 2-(o-Tolyl)quinazolin-4(3*H*)-one (**3ad**)

^{1}H NMR (600 MHz, DMSO-d$_6$)

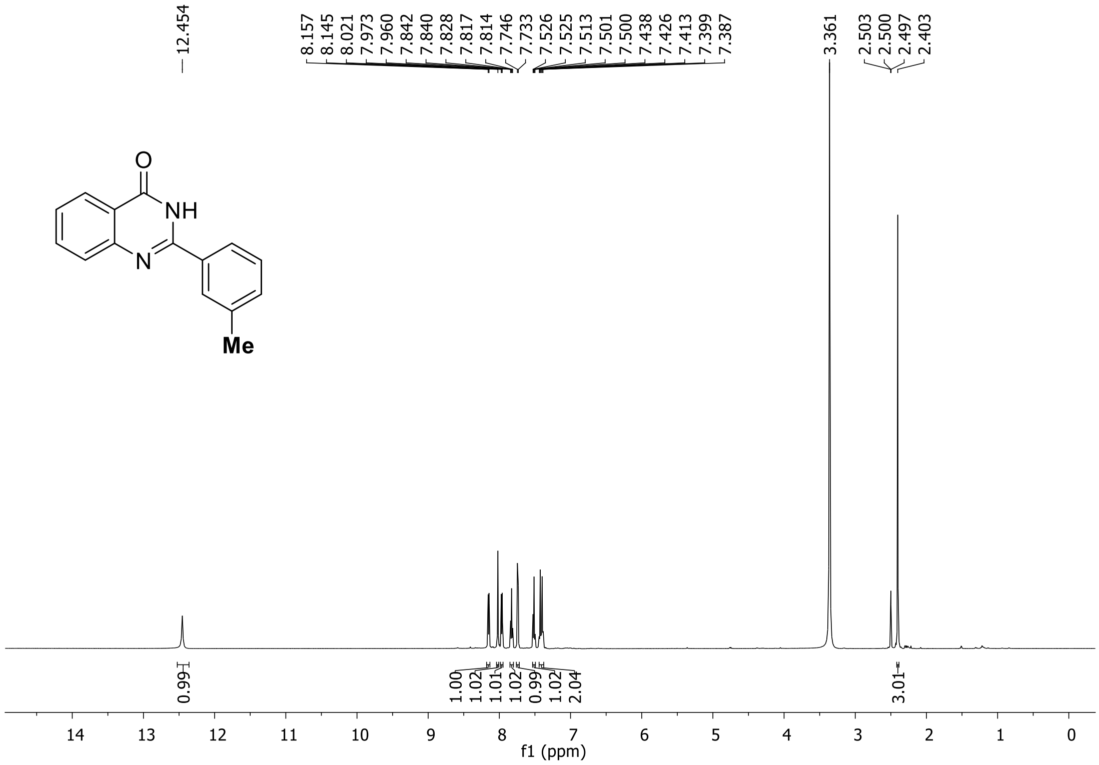

Figure S9. ^{1}H NMR spectrum of 2-(m-Tolyl)quinazolin-4(3*H*)-one (**3ae**)

^{13}C NMR (151 MHz, DMSO-d$_6$)

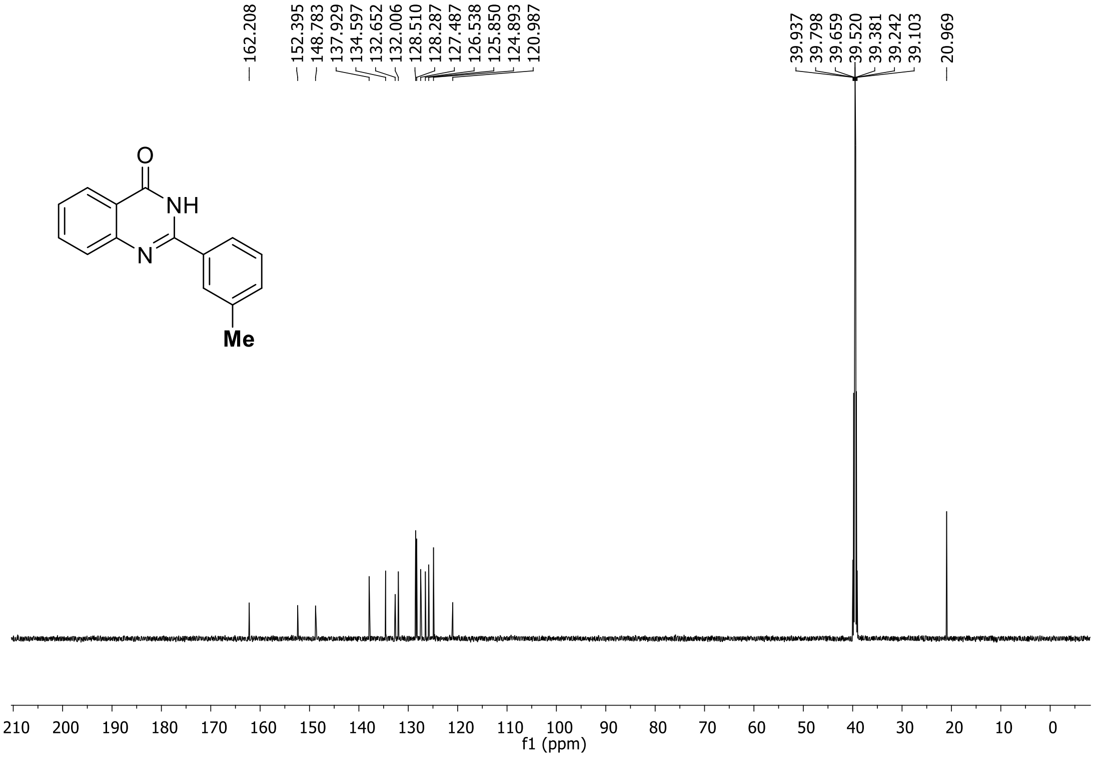

Figure S10. ^{13}C NMR spectrum of 2-(m-Tolyl)quinazolin-4(3*H*)-one (**3ae**)

^{1}H NMR (600 MHz, DMSO-d$_6$)

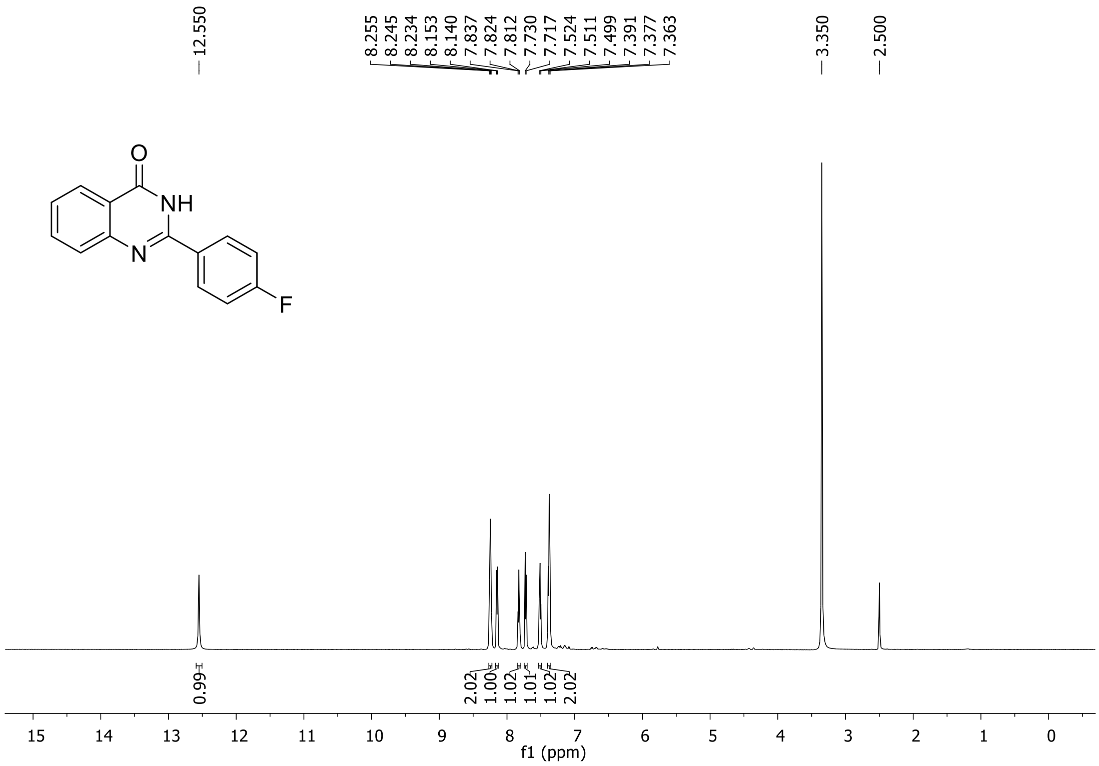

Figure S11. ^{1}H NMR spectrum of 2-(4-Fluorophenyl)quinazolin-4(3*H*)-one (**3af**)

^{13}C NMR (151 MHz, DMSO-d$_6$)

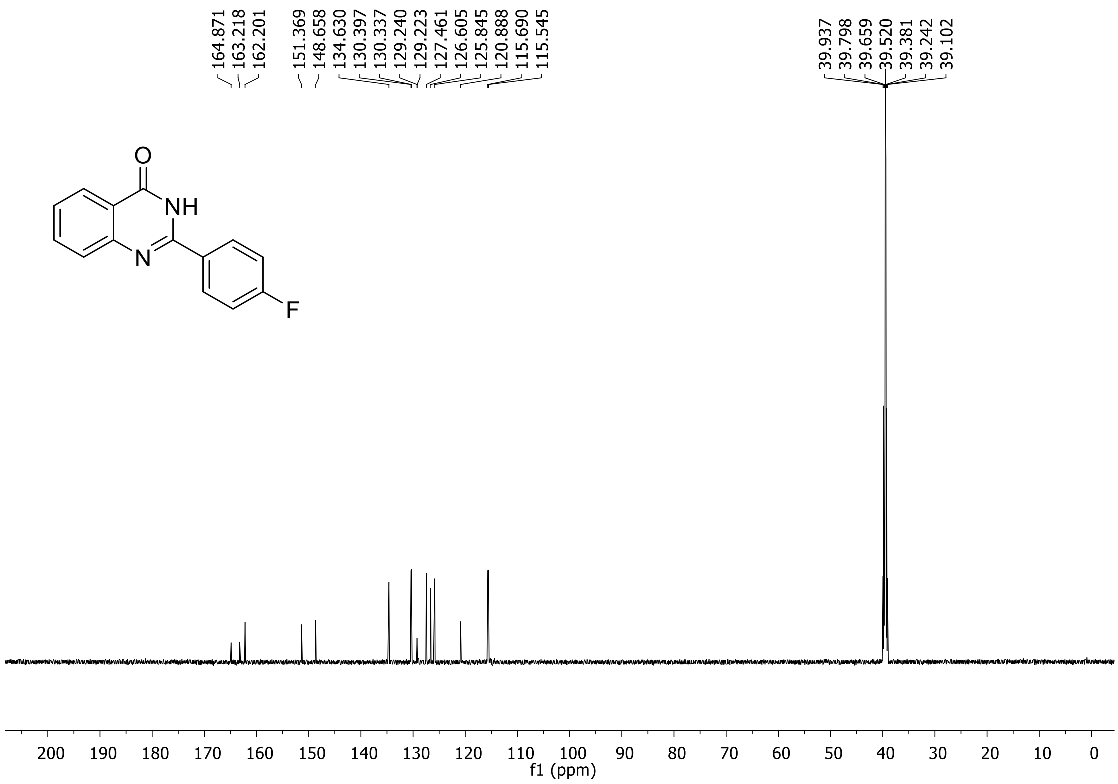

Figure S12. ^{13}C NMR spectrum of 2-(4-Fluorophenyl)quinazolin-4(3*H*)-one (**3af**)

^{1}H NMR (600 MHz, DMSO-d$_6$)

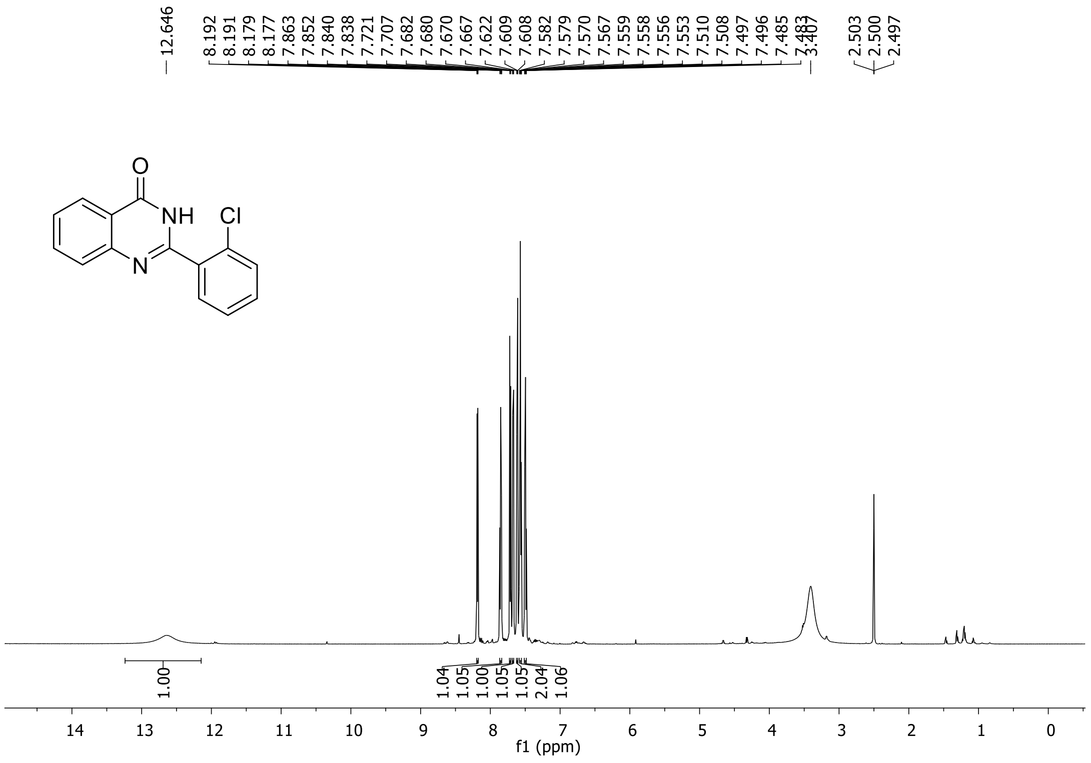

Figure S13. ^{1}H NMR spectrum of 2-(2-Chlorophenyl)quinazolin-4(3*H*)-one (**3ag**)

^{13}C NMR (151 MHz, DMSO-d$_6$)

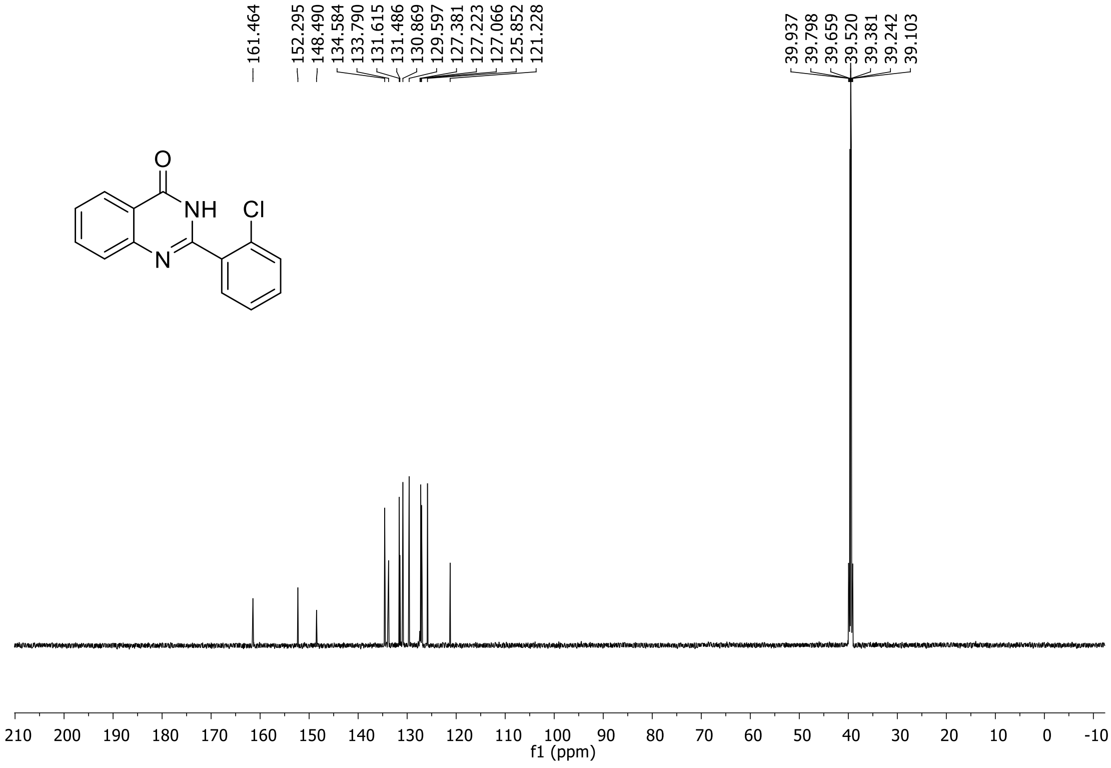

Figure S14. ^{13}C NMR spectrum of 2-(2-Chlorophenyl)quinazolin-4(3*H*)-one (**3ag**)

^{1}H NMR (600 MHz, DMSO-d$_6$)

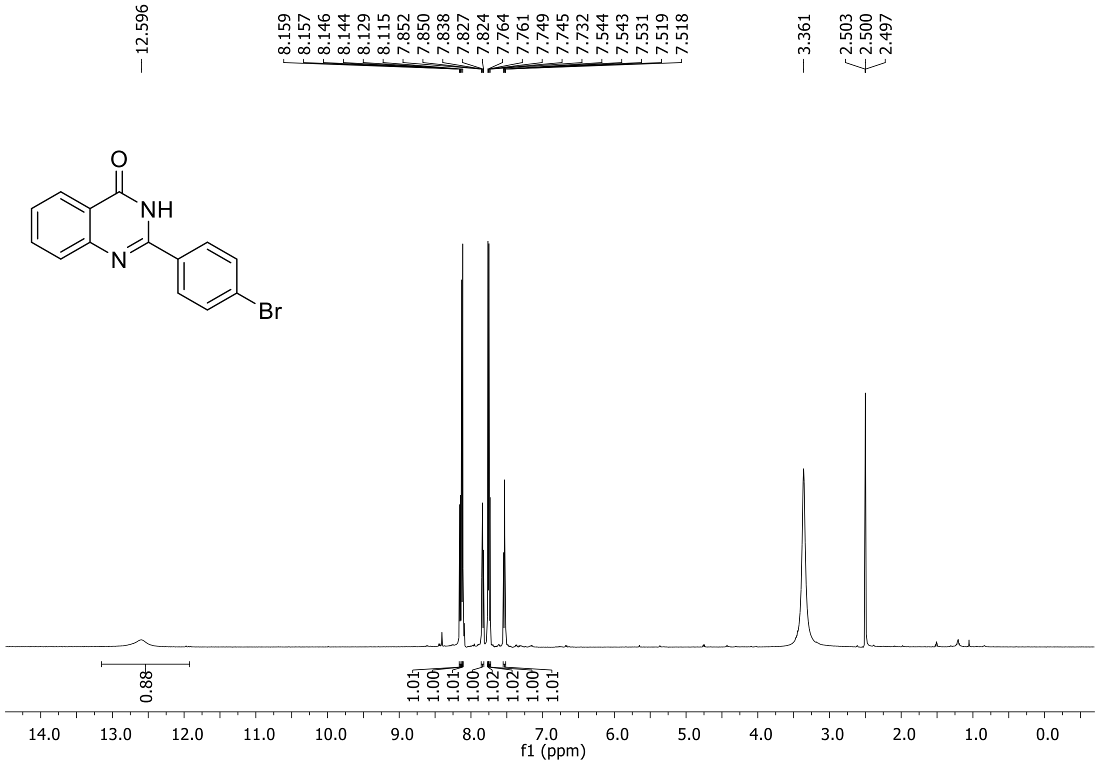

Figure S15. ^{1}H NMR spectrum of 2-(4-Bromophenyl)quinazolin-4(3*H*)-one (**3ai**)

^{13}C NMR (151 MHz, DMSO-d$_6$)

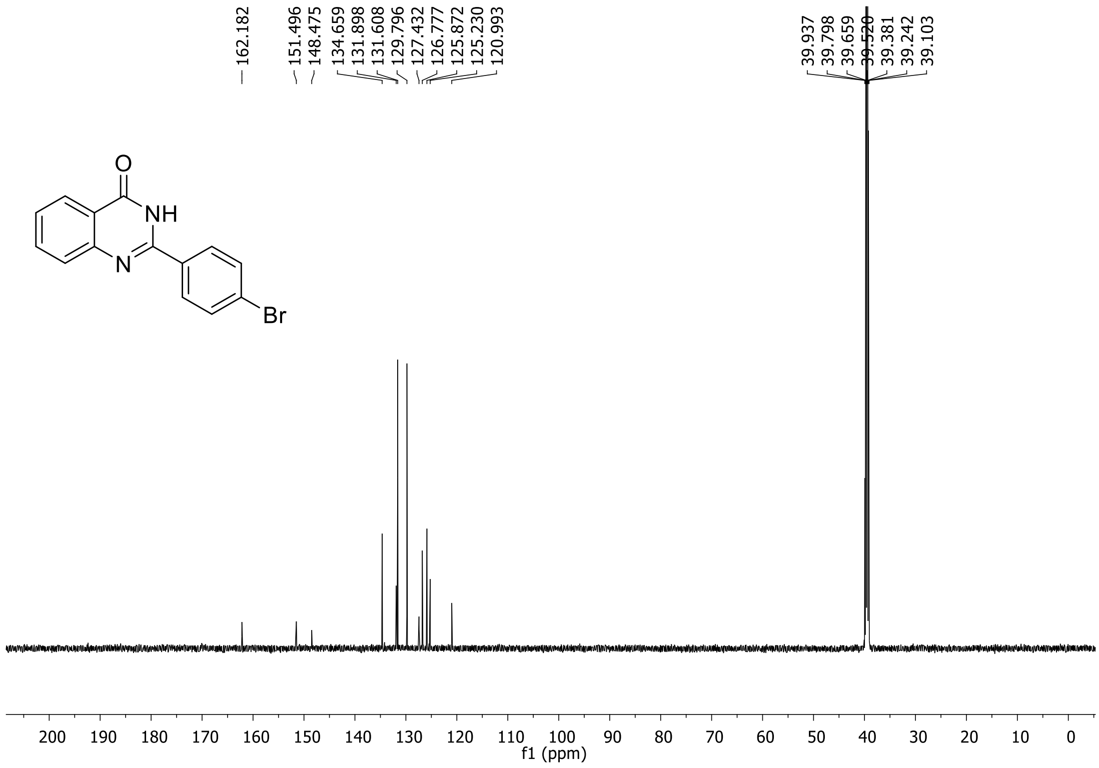

Figure S16. ^{13}C NMR spectrum of 2-(4-Bromophenyl)quinazolin-4(3*H*)-one (**3ai**)

^{1}H NMR (600 MHz, DMSO-d$_6$)

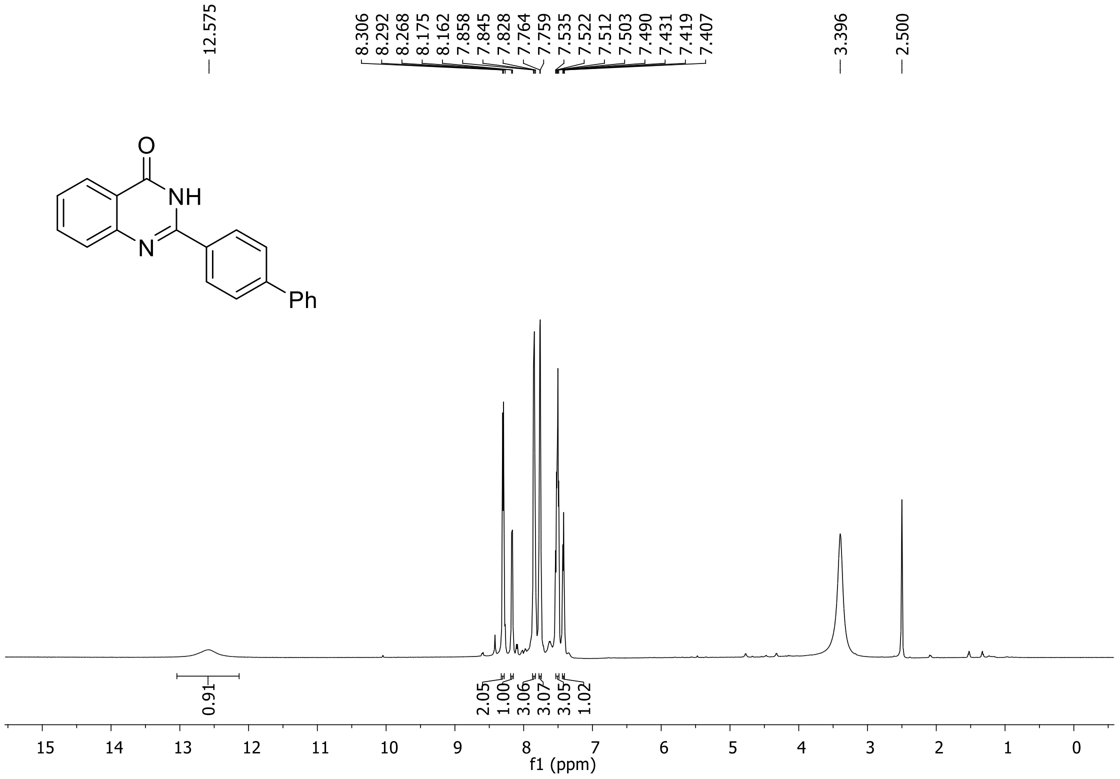

Figure S17. ^{1}H NMR spectrum of 2-([1,1'-Biphenyl]-4-yl)quinazolin-4(3*H*)-one (**3aj**)

^{13}C NMR (151 MHz, DMSO-d$_6$)

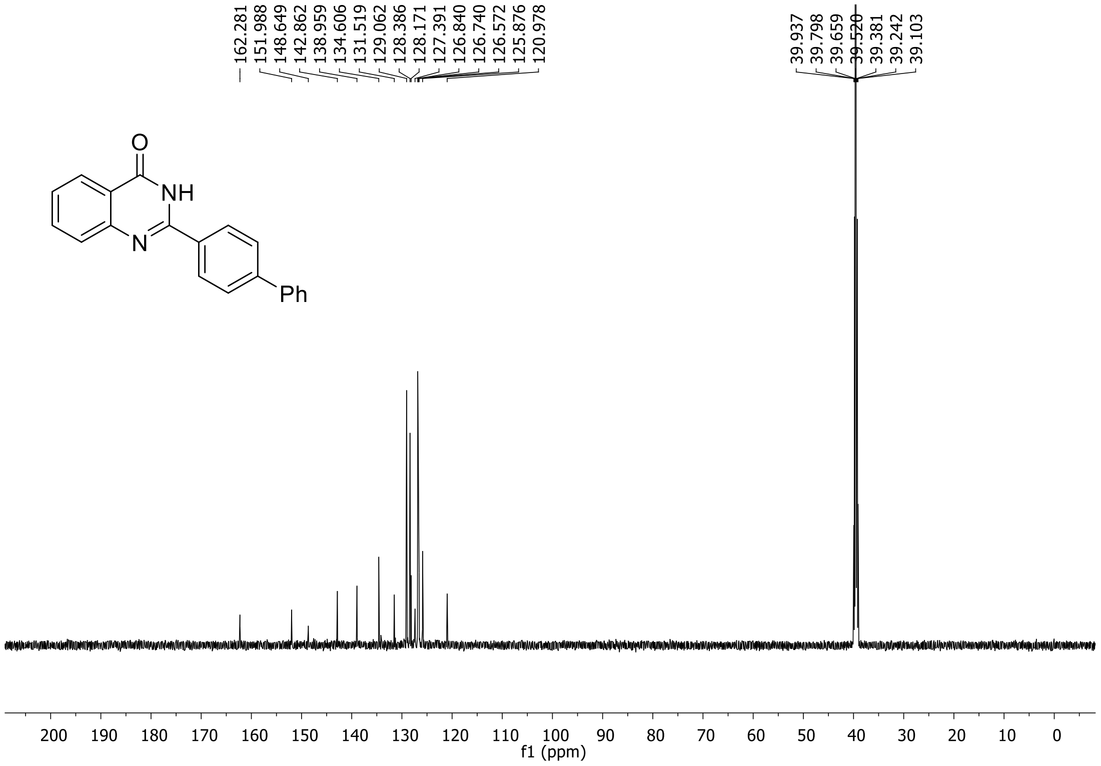

Figure S18. ^{13}C NMR spectrum of 2-([1,1'-Biphenyl]-4-yl)quinazolin-4(3*H*)-one (**3aj**)

^{1}H NMR (600 MHz, DMSO-d$_6$)

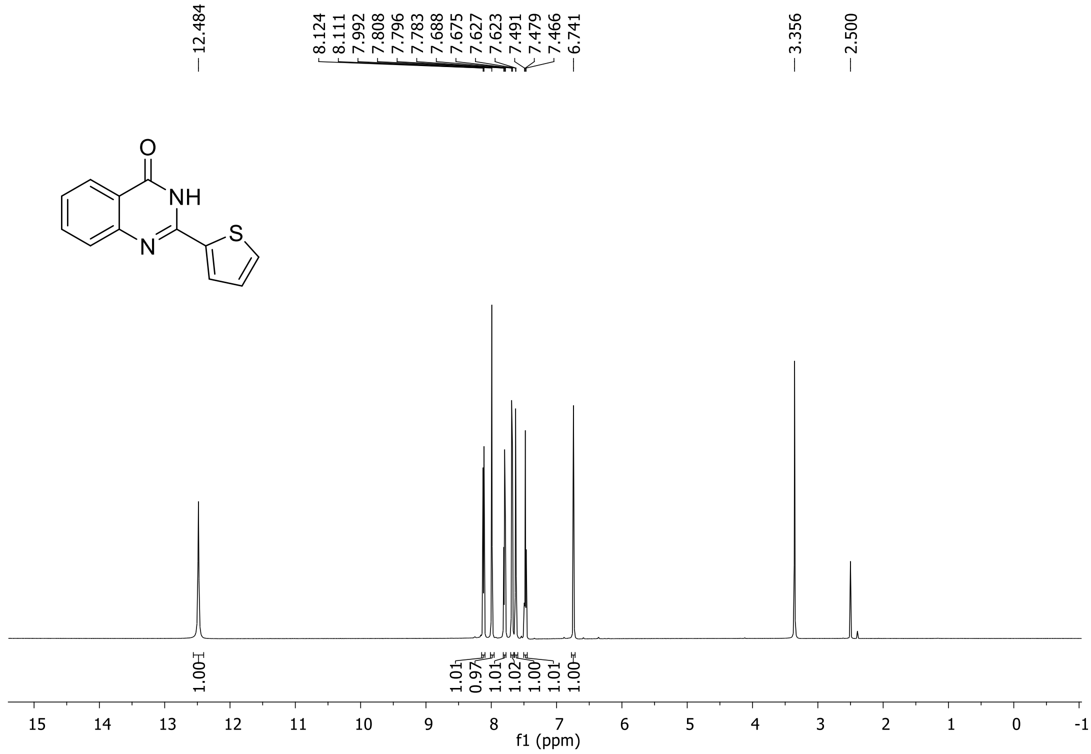

Figure S19. ^{1}H NMR spectrum of 2-(Thiophen-2-yl)quinazolin-4(3*H*)-one (**3ak**)

^{13}C NMR (151 MHz, DMSO-d$_6$)

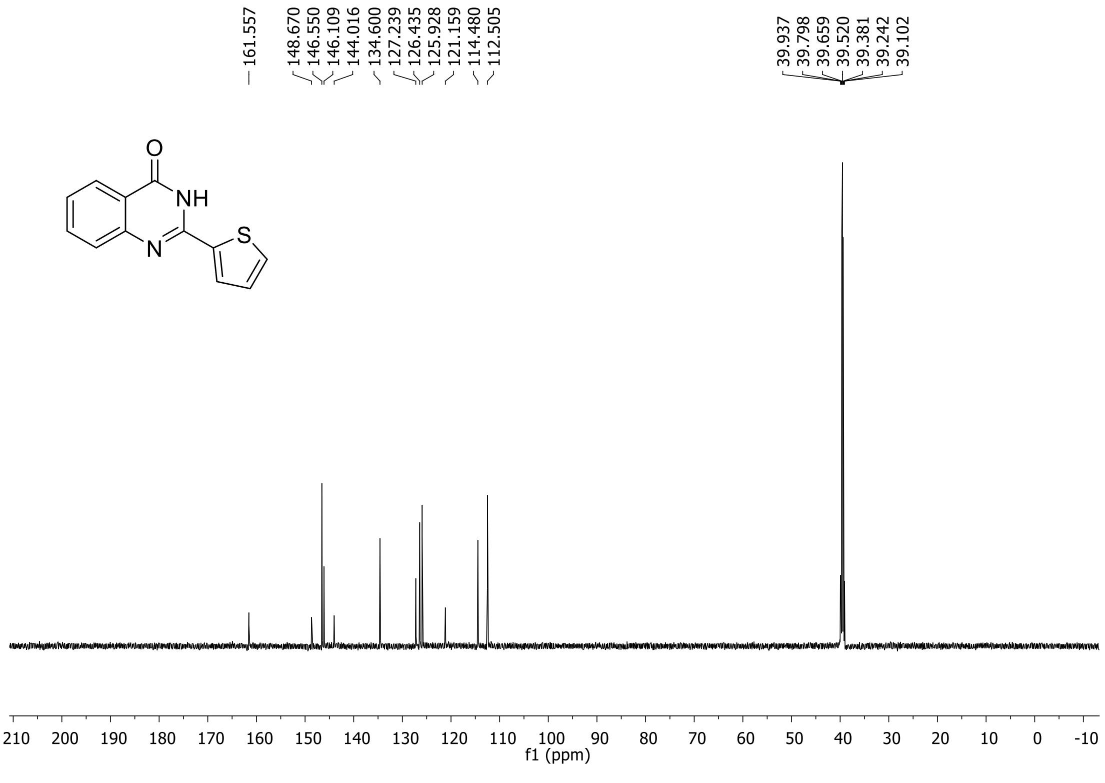

Figure S20. ^{13}C NMR spectrum of 2-(Thiophen-2-yl)quinazolin-4(3*H*)-one (**3ak**)

^{1}H NMR (600 MHz, DMSO-d$_6$)

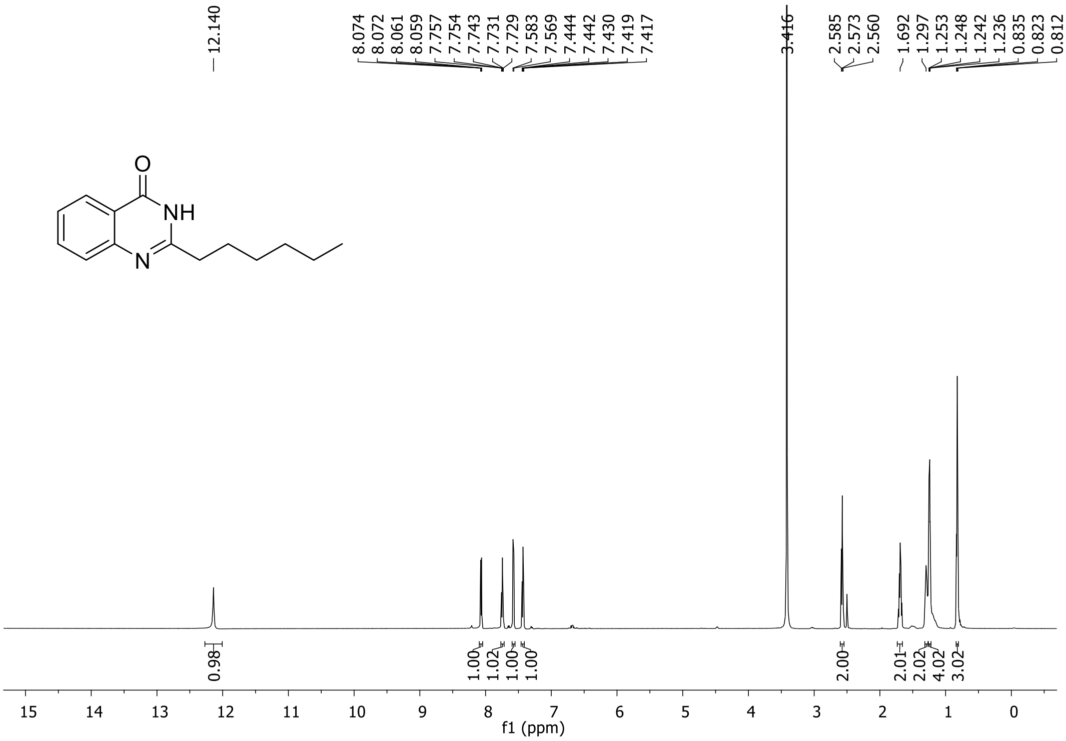

Figure S21. ^{1}H NMR spectrum of 2-Hexylquinazolin-4(3*H*)-one (**3al**)

^{13}C NMR (151 MHz, DMSO-d$_6$)

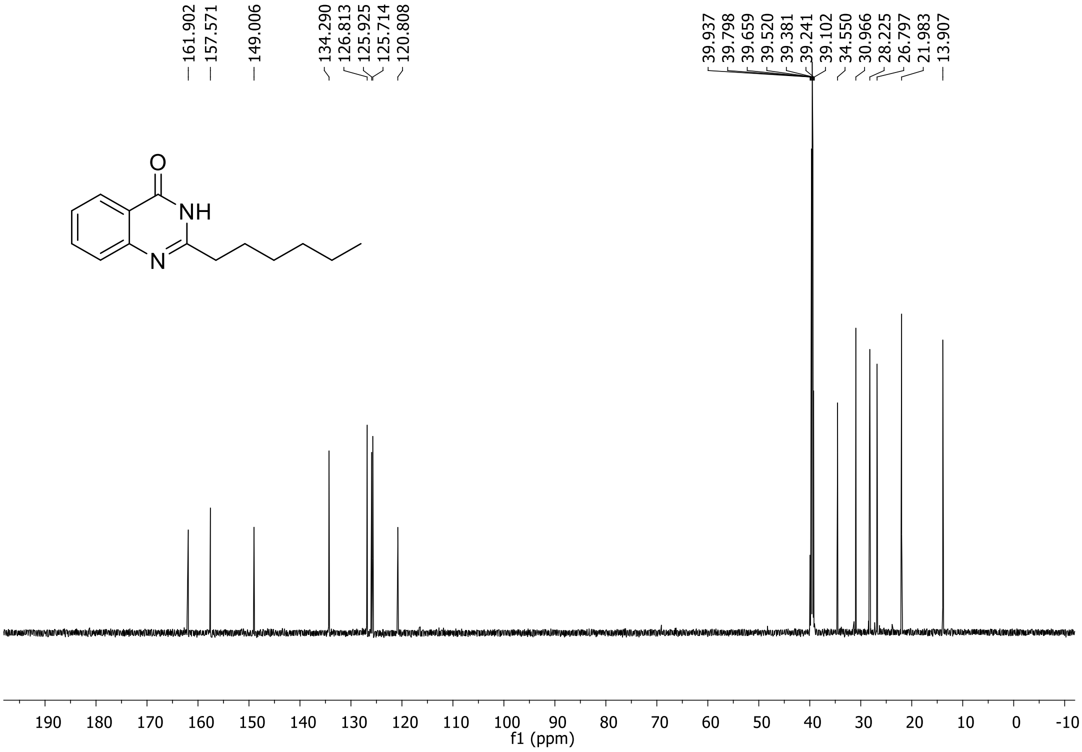

Figure S22. ^{13}C NMR spectrum of 2-Hexylquinazolin-4(3*H*)-one (**3al**)

^{1}H NMR (600 MHz, DMSO-d$_6$)

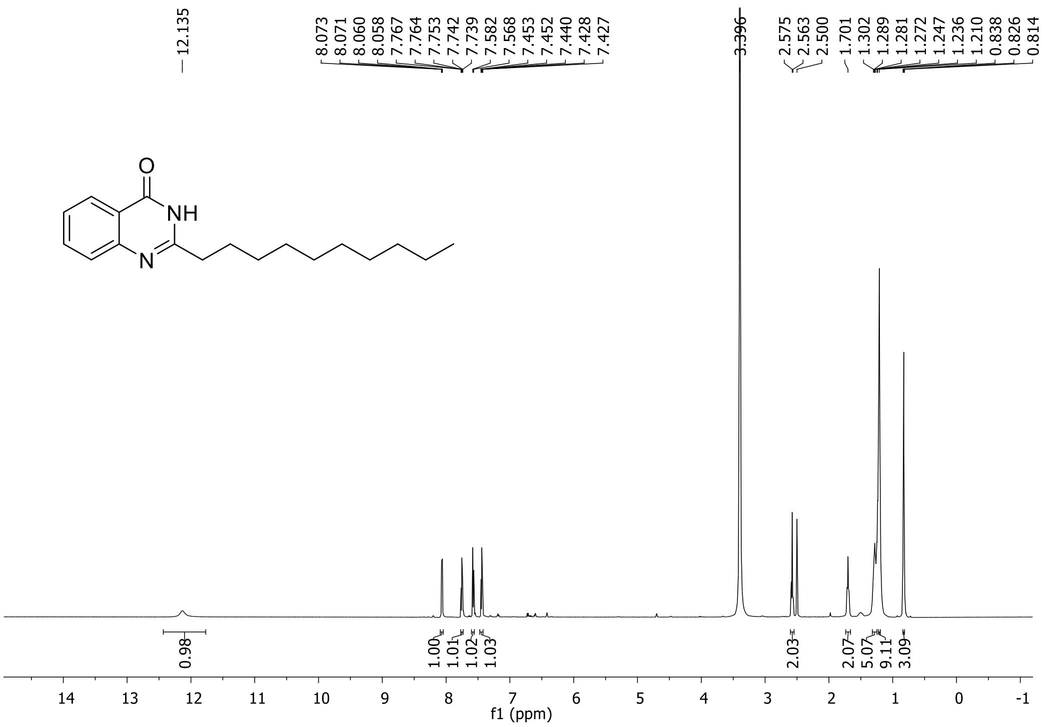

Figure S23. ^{1}H NMR spectrum of 2-Decylquinazolin-4(3*H*)-one (**3am**)

^{13}C NMR (151 MHz, DMSO-d$_6$)

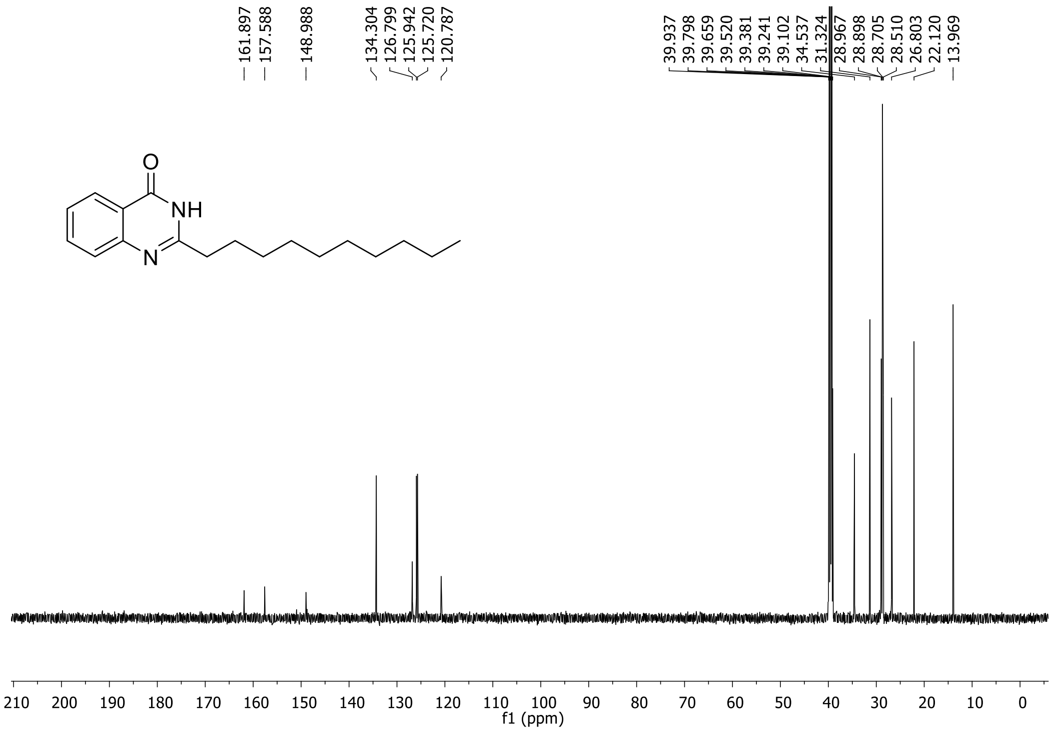

Figure S24. ^{13}C NMR spectrum of 2-Decylquinazolin-4(3*H*)-one (**3am**)

^{1}H NMR (600 MHz, DMSO-d$_6$)

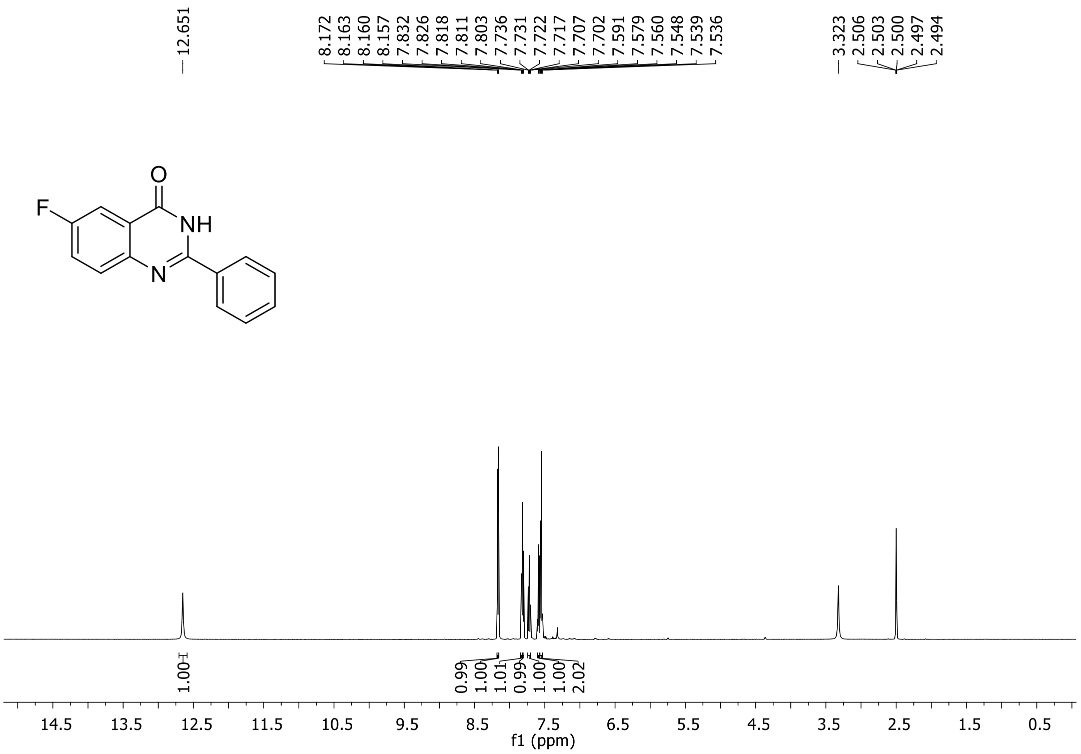

Figure S25. ^{1}H NMR spectrum of 6-Fluoro-2-phenylquinazolin-4(3*H*)-one (**3ba**)

^{13}C NMR (151 MHz, DMSO-d$_6$)

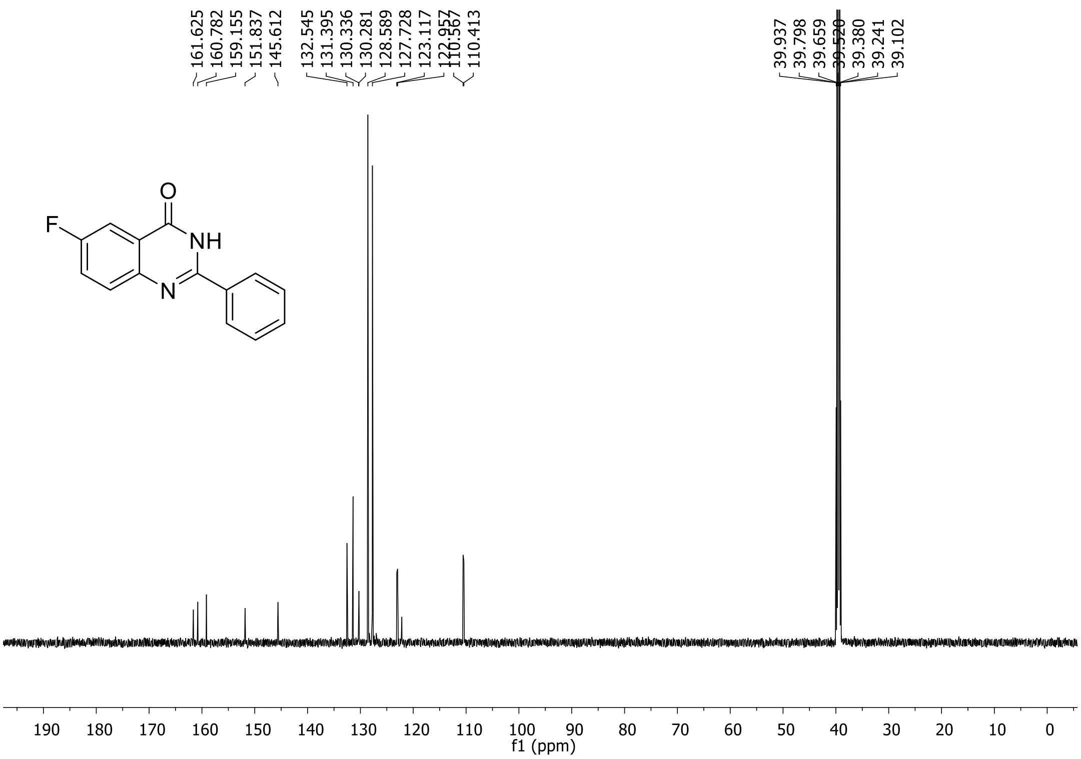

Figure S26. ^{13}C NMR spectrum of 6-Fluoro-2-phenylquinazolin-4(3*H*)-one (**3ba**)

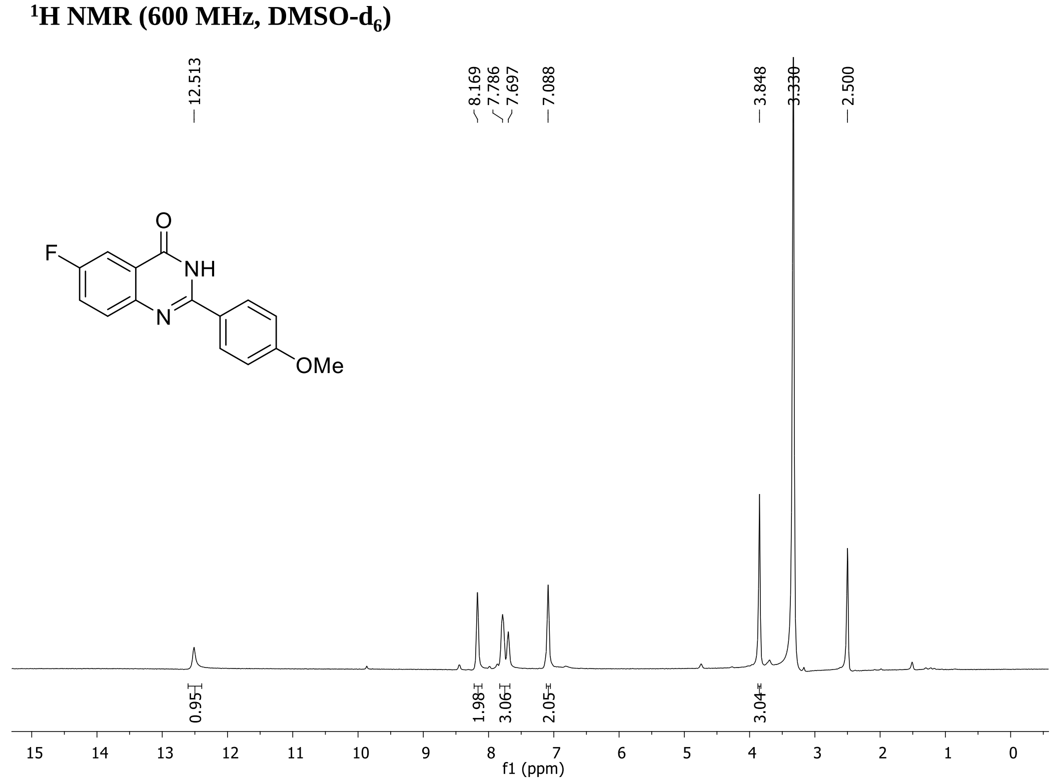

Figure S27. ¹H NMR spectrum of 6-Fluoro-2-(4-methoxyphenyl)quinazolin-4(3*H*)-one (**3bb)**

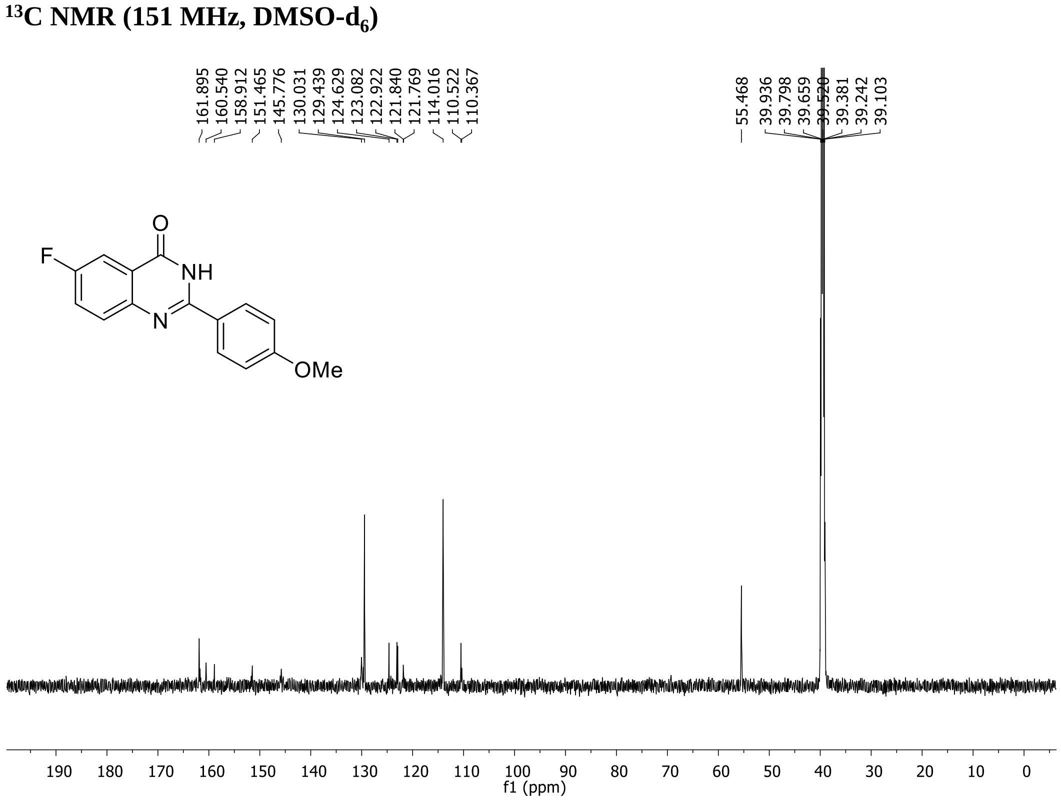

Figure S28. ¹³C NMR spectrum of 6-Fluoro-2-(4-methoxyphenyl)quinazolin-4(3*H*)-one (**3bb)**

^{1}H NMR (600 MHz, DMSO-d$_6$)

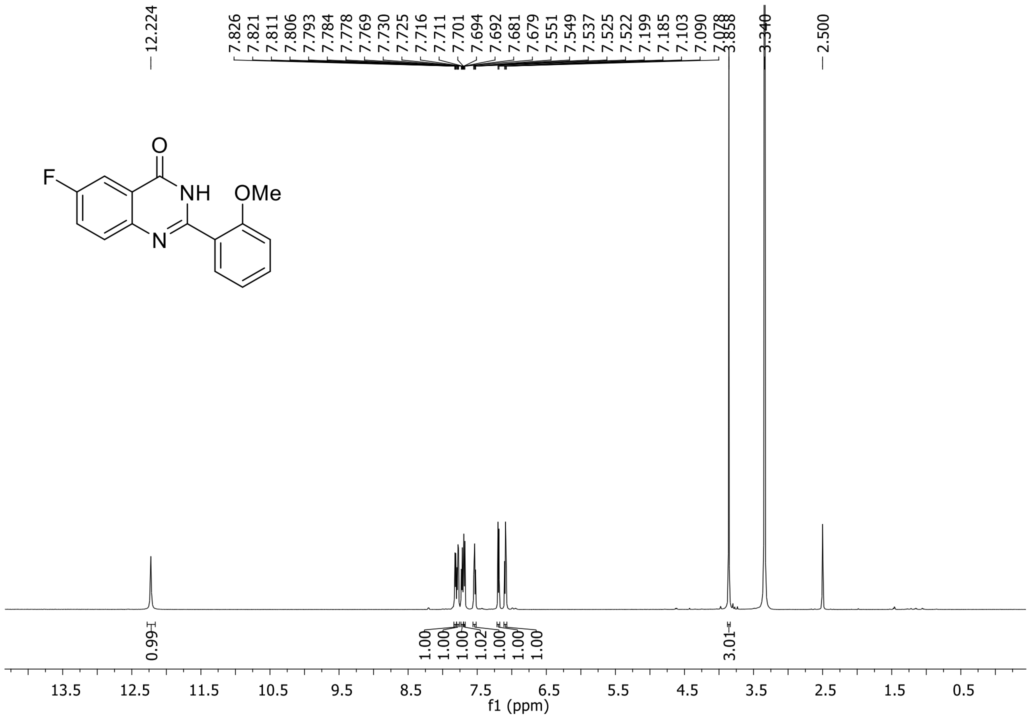

Figure S29. ^{1}H NMR spectrum of 6-Fluoro-2-(2-methoxyphenyl)quinazolin-4(3*H*)-one **(3bc)**

^{13}C NMR (151 MHz, DMSO-d$_6$)

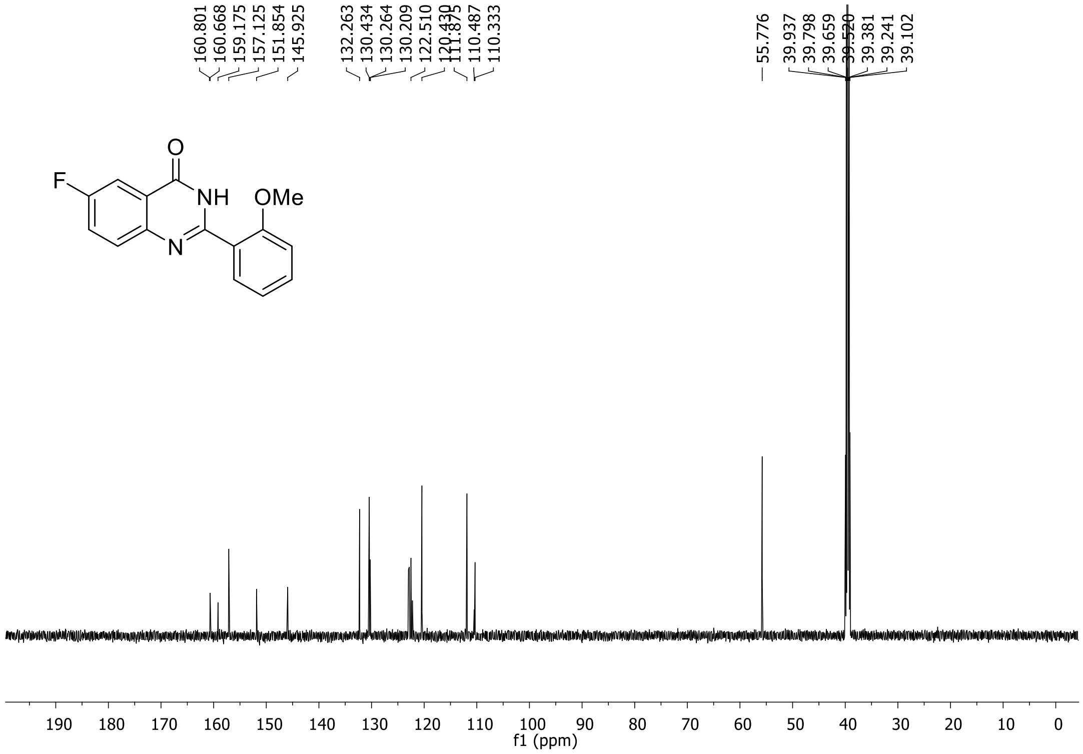

Figure S30. ^{13}C NMR spectrum of 6-Fluoro-2-(2-methoxyphenyl)quinazolin-4(3*H*)-one **(3bc)**

^{1}H NMR (600 MHz, DMSO-d$_6$)

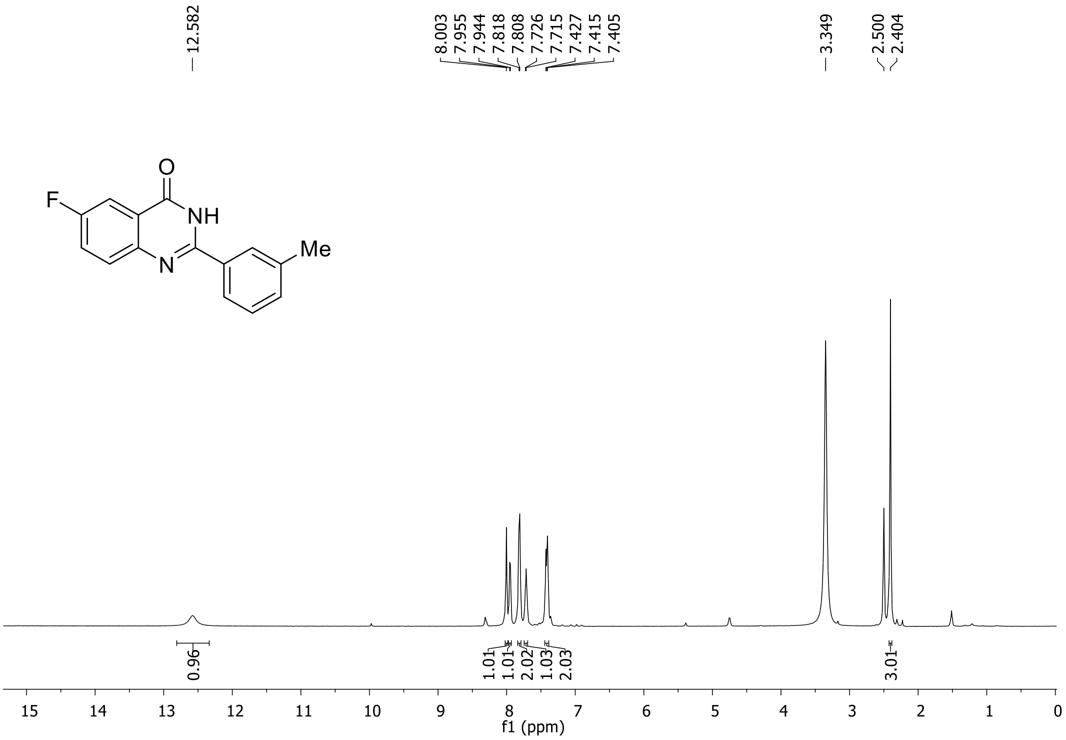

Figure S31. ^{1}H NMR spectrum of 6-Fluoro-2-(m-tolyl)quinazolin-4(3*H*)-one (**3be**)

^{13}C NMR (151 MHz, DMSO-d$_6$)

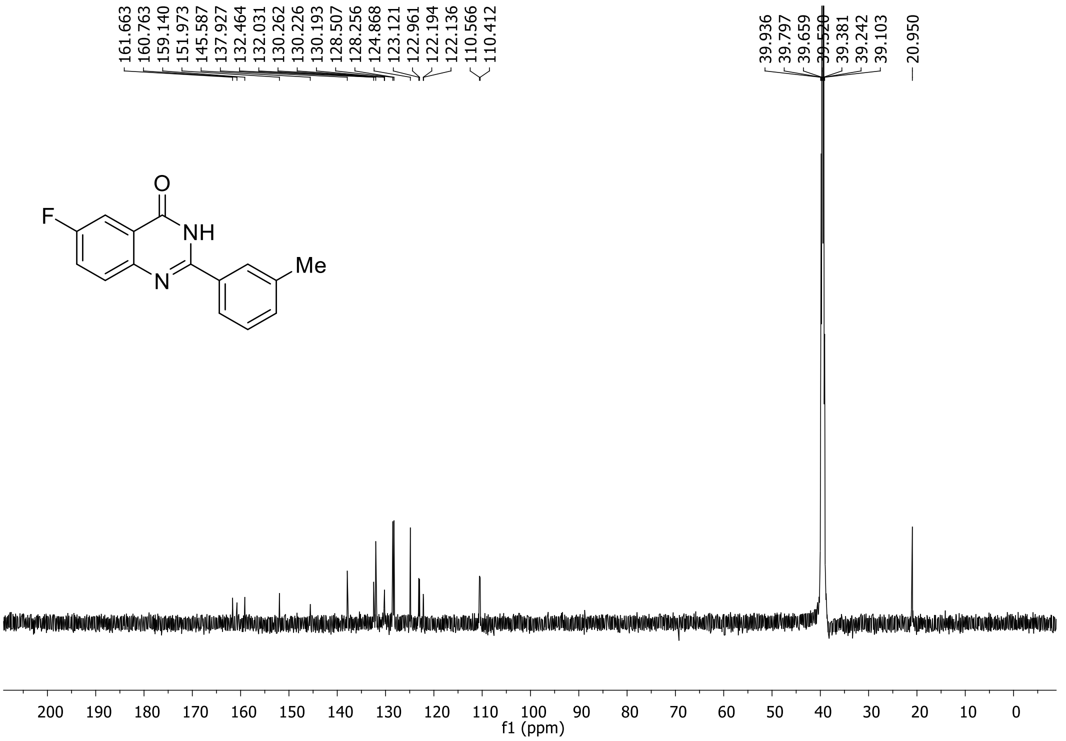

Figure S32. ^{13}C NMR spectrum of 6-Fluoro-2-(m-tolyl)quinazolin-4(3*H*)-one (**3be**)

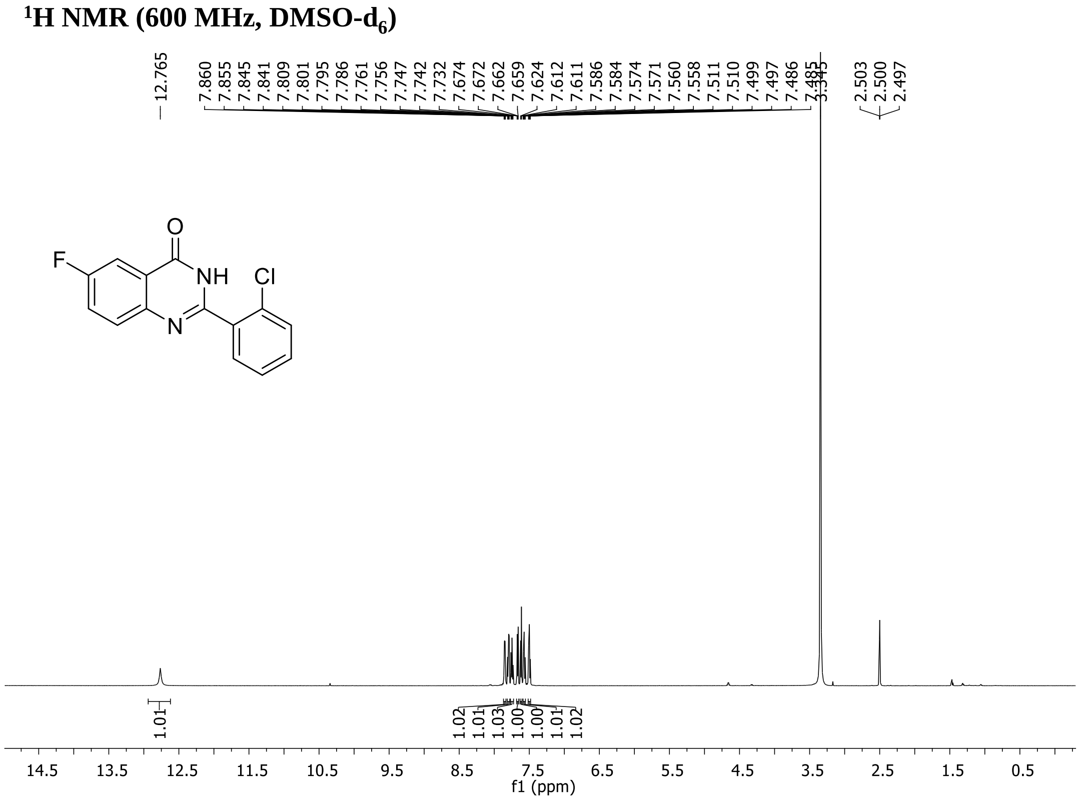

Figure S33. ^{1}H NMR spectrum of 2-(2-Chlorophenyl)-6-fluoroquinazolin-4(3*H*)-one (**3bg**)

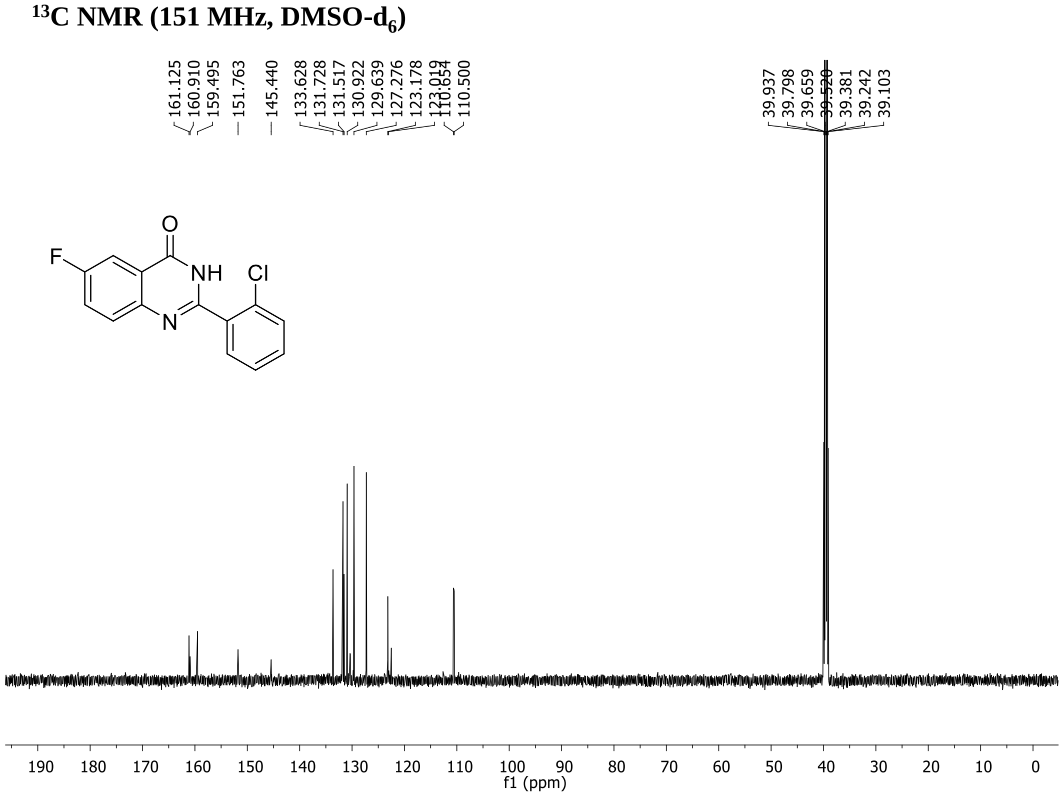

Figure S34. ^{13}C NMR spectrum of 2-(2-Chlorophenyl)-6-fluoroquinazolin-4(3*H*)-one (**3bg**)

^{1}H NMR (600 MHz, DMSO-d$_6$)

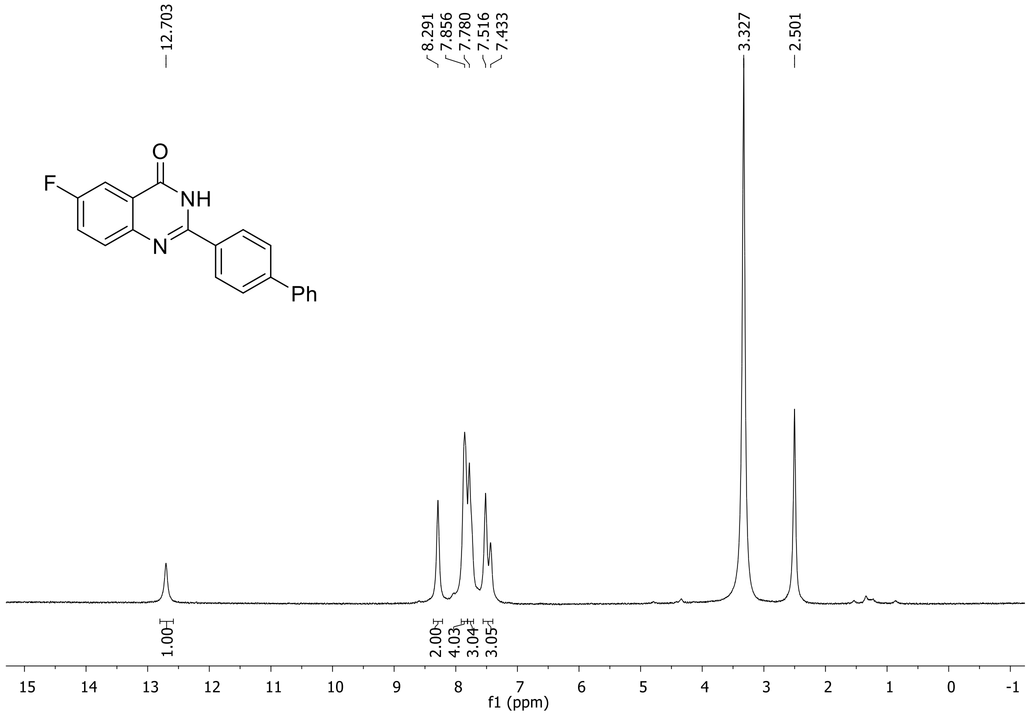

Figure S35. ^{1}H NMR spectrum of 2-([1,1'-Biphenyl]-4-yl)-6-fluoroquinazolin-4(3*H*)-one (**3bj**)

^{13}C NMR (151 MHz, DMSO-d$_6$)

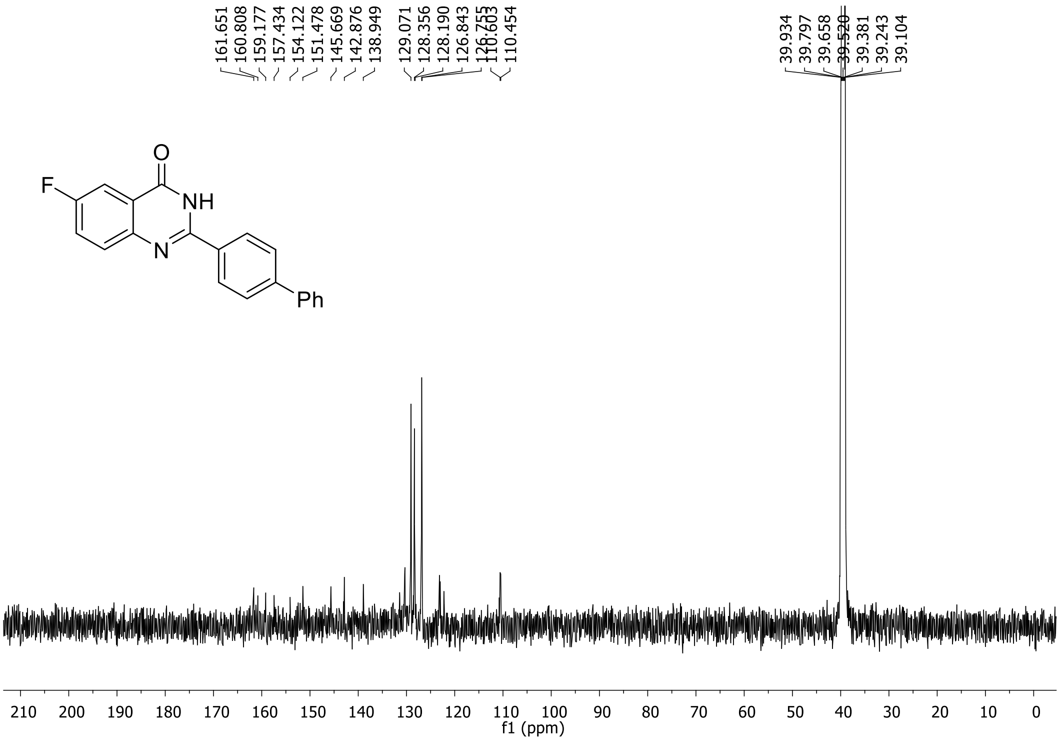

Figure S36. ^{13}C NMR spectrum of 2-([1,1'-Biphenyl]-4-yl)-6-fluoroquinazolin-4(3*H*)-one (**3bj**)

^{1}H NMR (600 MHz, DMSO-d$_6$)

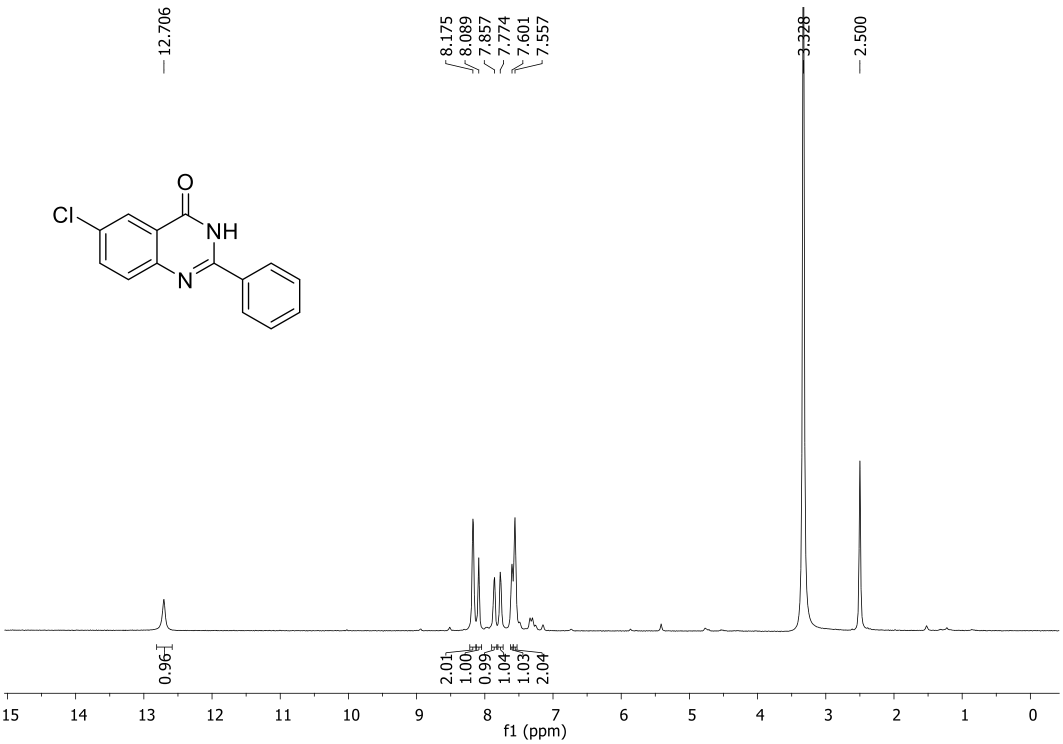

Figure S37. ^{1}H NMR spectrum of 6-Chloro-2-phenylquinazolin-4(3*H*)-one (**3ca**)

^{13}C NMR (151 MHz, DMSO-d$_6$)

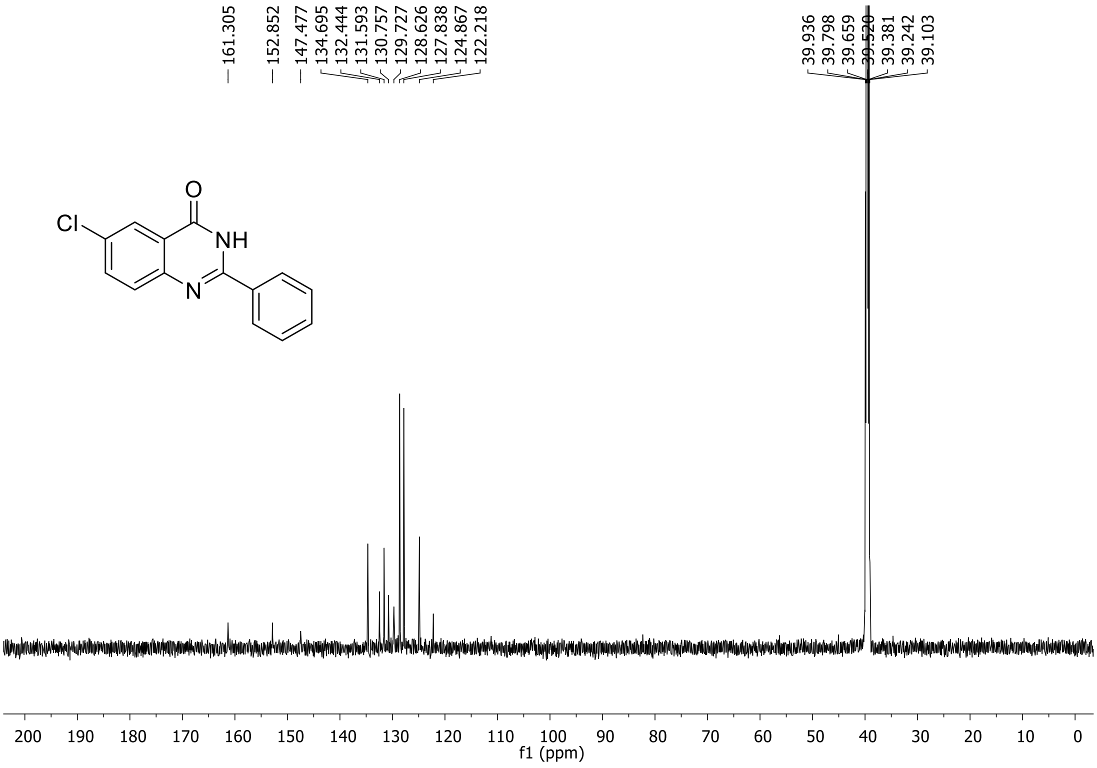

Figure S38. ^{13}C NMR spectrum of 6-Chloro-2-phenylquinazolin-4(3*H*)-one (**3ca**)

^{1}H NMR (600 MHz, DMSO-d$_6$)

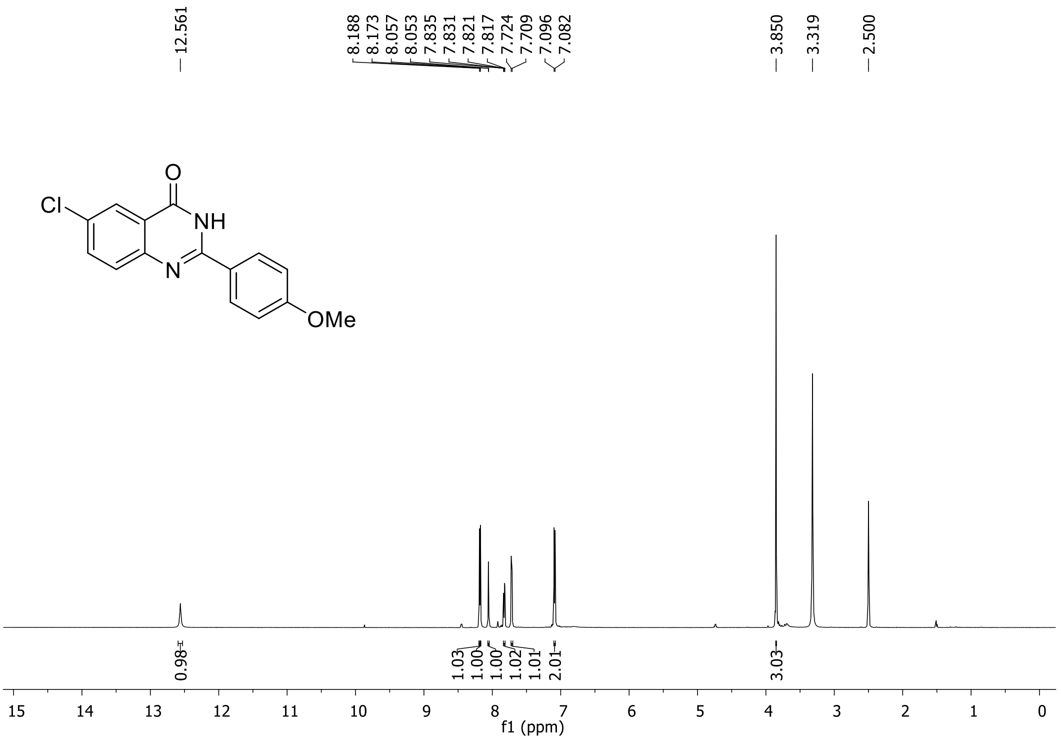

Figure S39. ^{1}H NMR spectrum of 6-Chloro-2-(4-methoxyphenyl)quinazolin-4(3*H*)-one (**3cb**)

^{13}C NMR (151 MHz, DMSO-d$_6$)

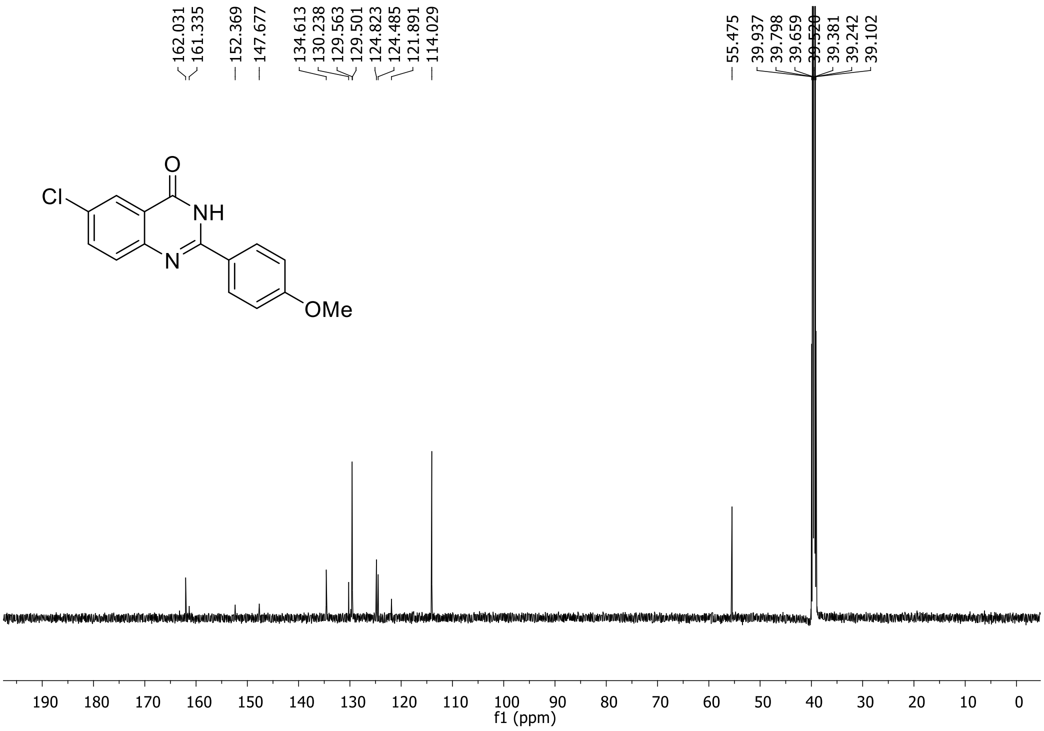

Figure S40. ^{13}C NMR spectrum of 6-Chloro-2-(4-methoxyphenyl)quinazolin-4(3*H*)-one (**3cb**)

^{1}H NMR (600 MHz, DMSO-d$_6$)

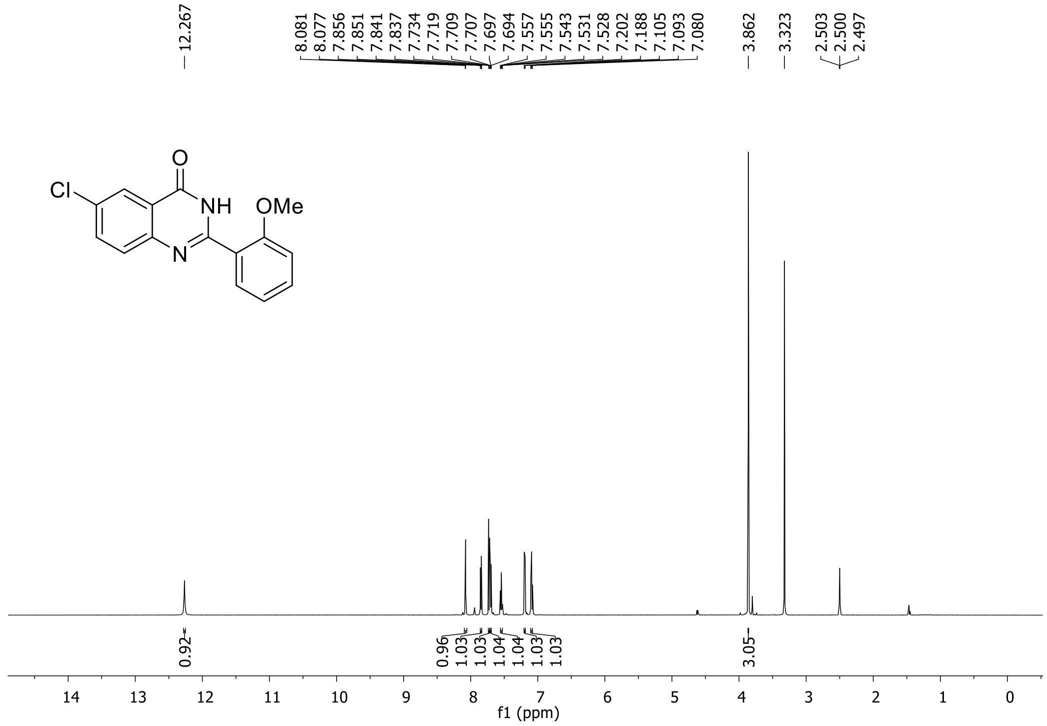

Figure S41. ^{1}H NMR spectrum of 6-Chloro-2-(2-methoxyphenyl)quinazolin-4(3*H*)-one (**3cc**)

^{13}C NMR (151 MHz, DMSO-d$_6$)

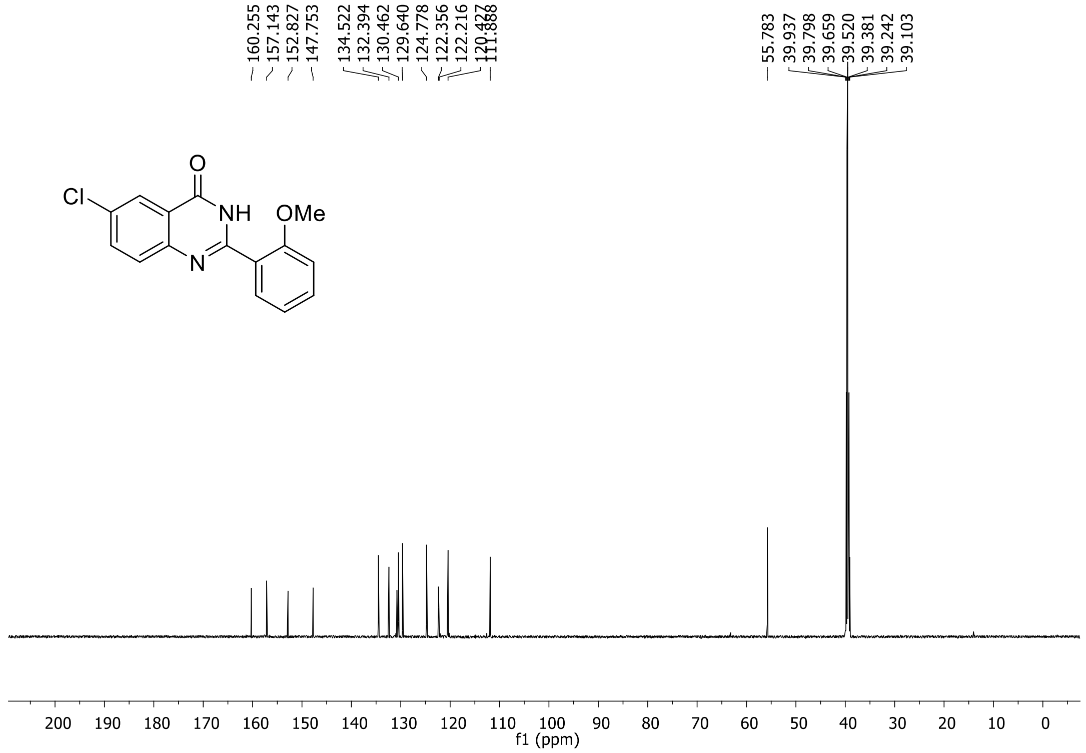

Figure S42. ^{13}C NMR spectrum of 6-Chloro-2-(2-methoxyphenyl)quinazolin-4(3*H*)-one (**3cc**)

^{1}H NMR (600 MHz, DMSO-d$_6$)

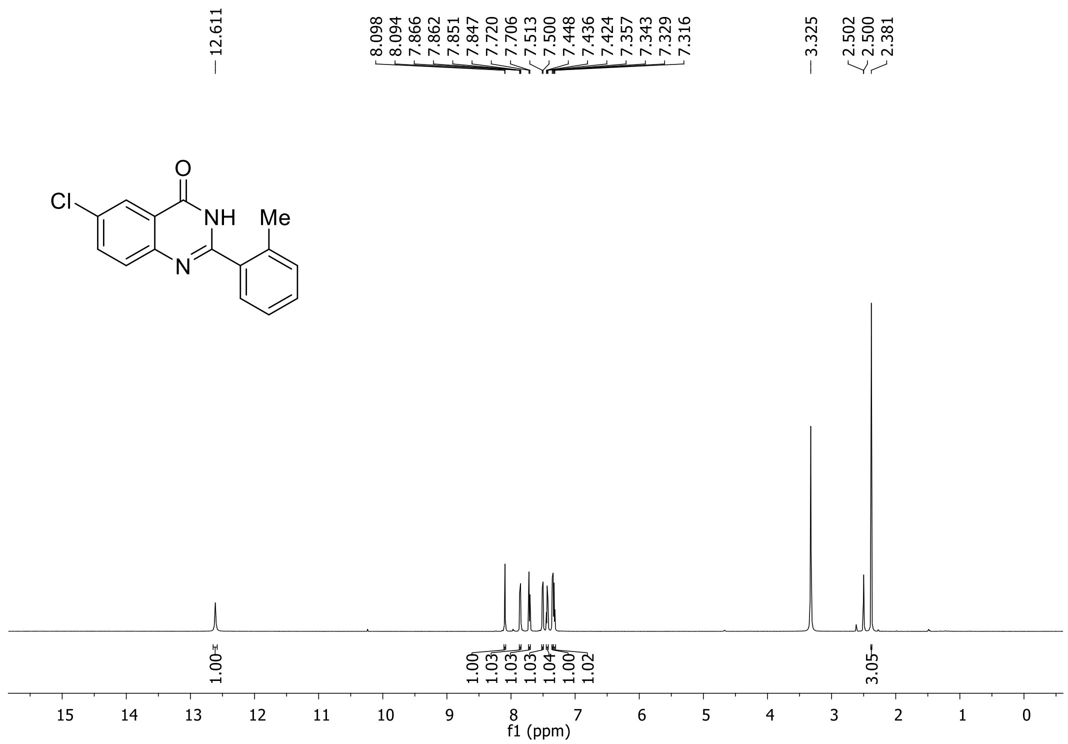

Figure S43. ^{1}H NMR spectrum of 6-Chloro-2-(o-tolyl)quinazolin-4(3*H*)-one (**3cd)**

^{13}C NMR (151 MHz, DMSO-d$_6$)

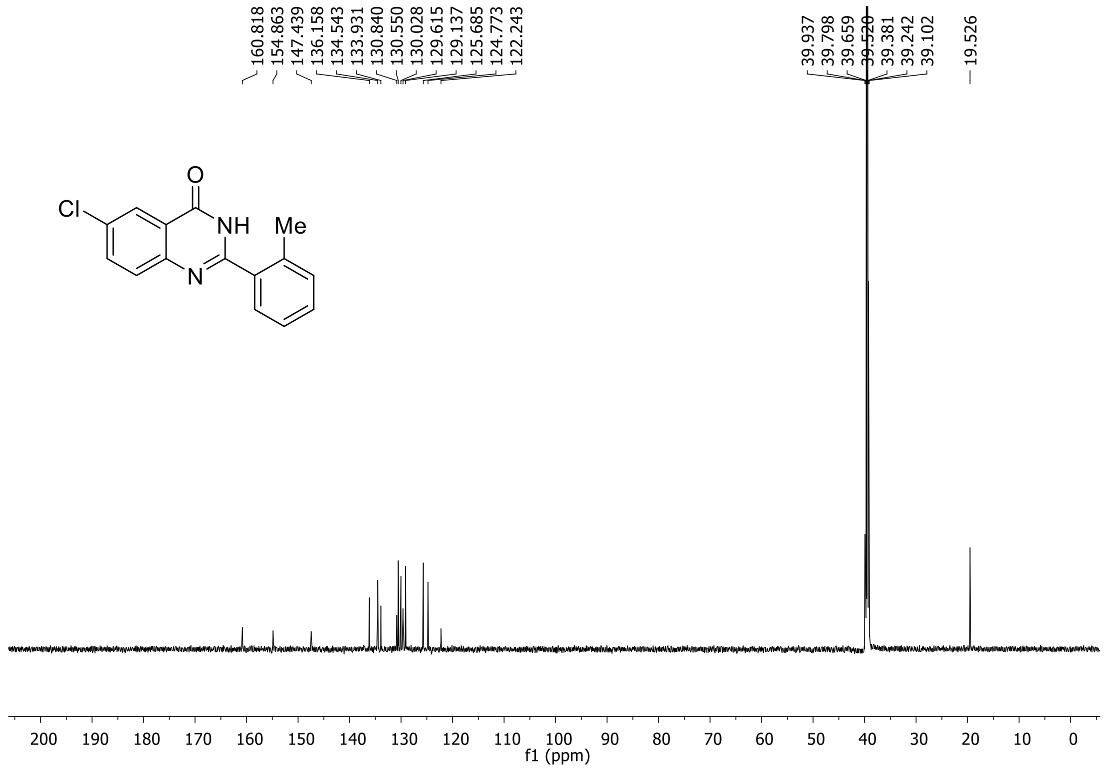

Figure S44. ^{13}C NMR spectrum of 6-Chloro-2-(o-tolyl)quinazolin-4(3*H*)-one (**3cd)**

^{1}H NMR (600 MHz, DMSO-d_6)

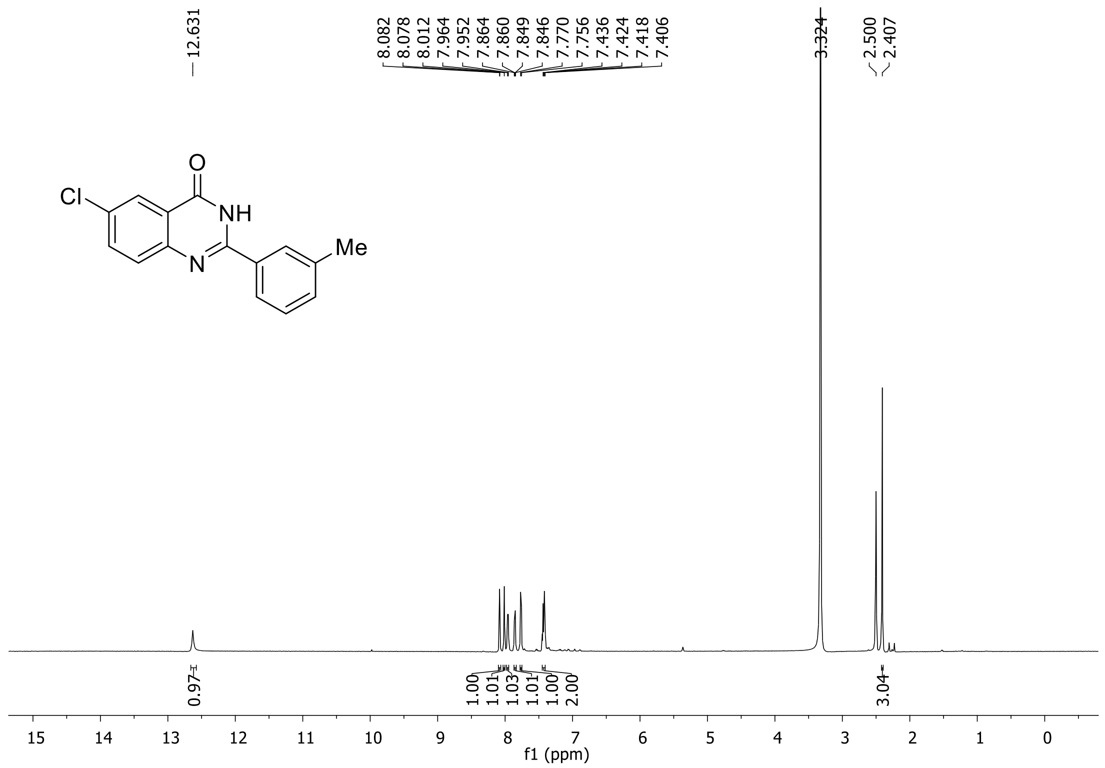

Figure S45. ^{1}H NMR spectrum of 6-Chloro-2-(m-tolyl)quinazolin-4(3*H*)-one (**3ce**)

^{13}C NMR (151 MHz, DMSO-d_6)

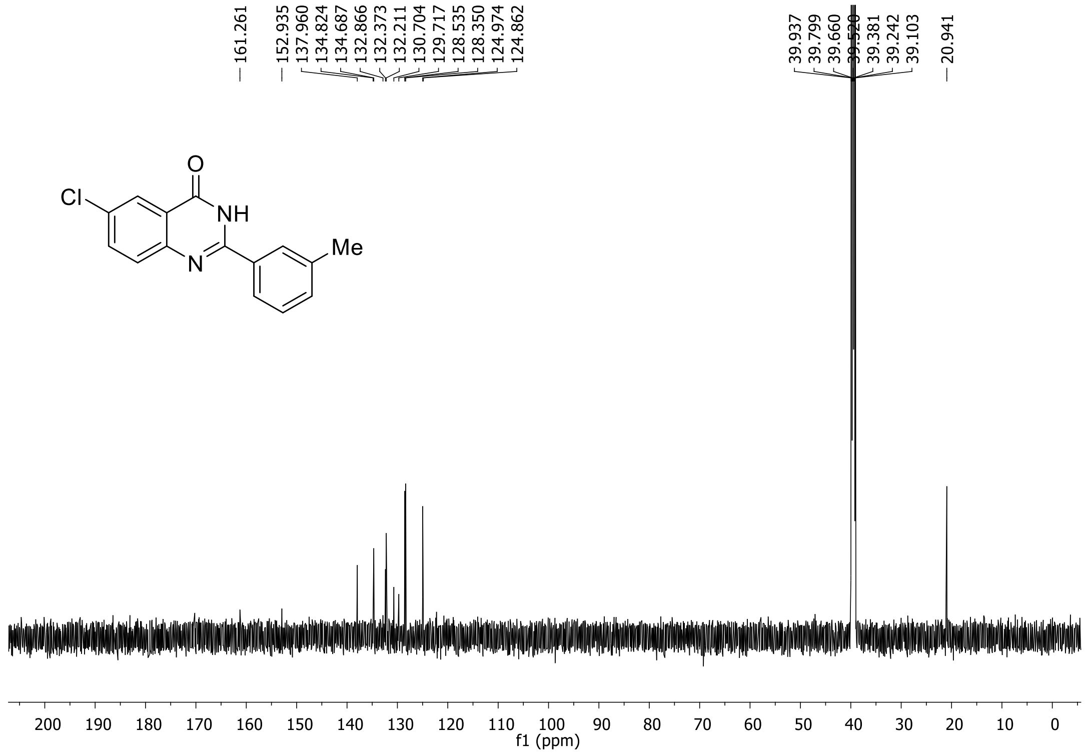

Figure S46. ^{13}C NMR spectrum of 6-Chloro-2-(m-tolyl)quinazolin-4(3*H*)-one (**3ce**)

^{1}H NMR (600 MHz, DMSO-d$_6$)

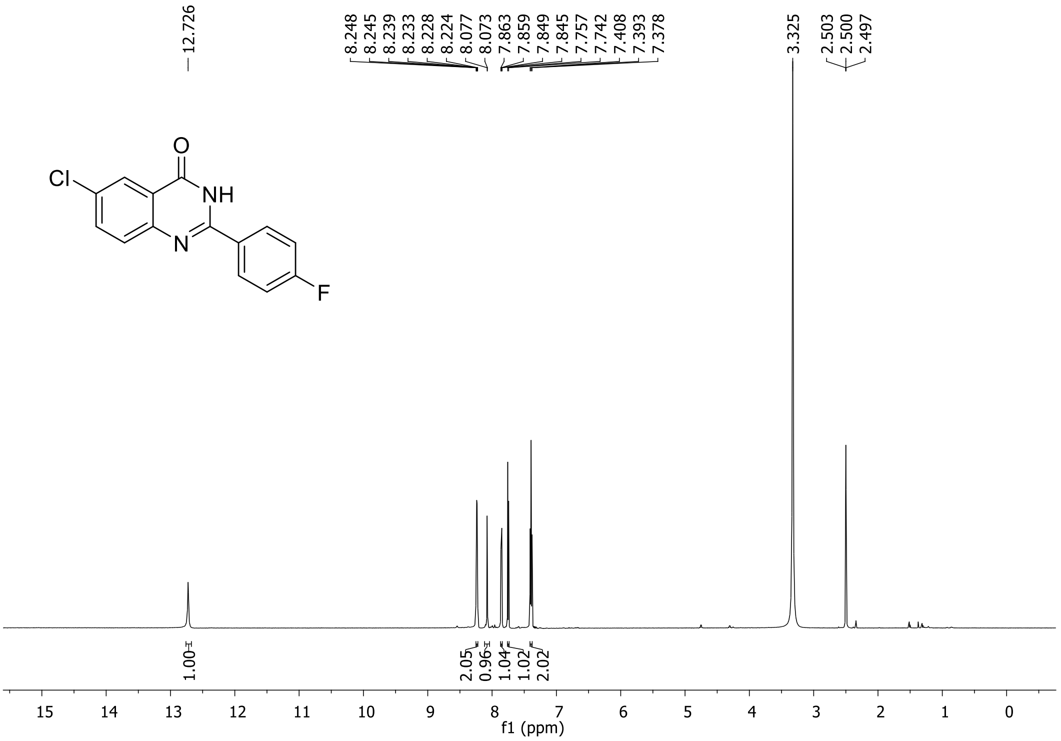

Figure S47. ^{1}H NMR spectrum of 6-Chloro-2-(4-fluorophenyl)quinazolin-4(3*H*)-one (**3cf)**

^{13}C NMR (151 MHz, DMSO-d$_6$)

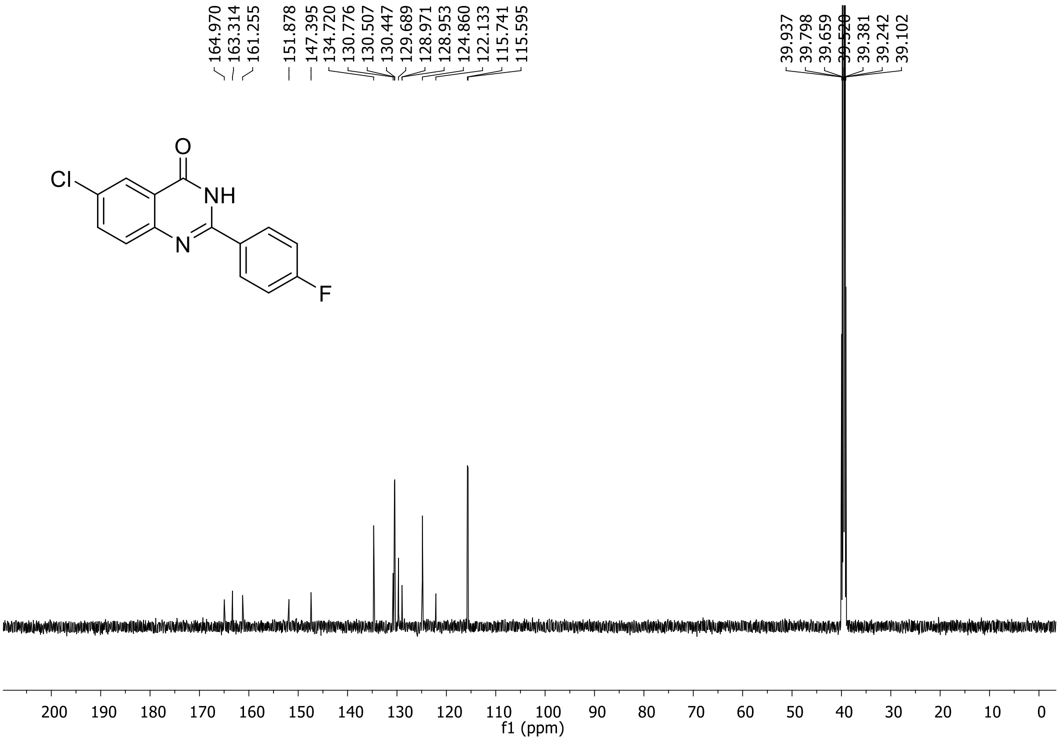

Figure S48. ^{13}C NMR spectrum of 6-Chloro-2-(4-fluorophenyl)quinazolin-4(3*H*)-one (**3cf)**

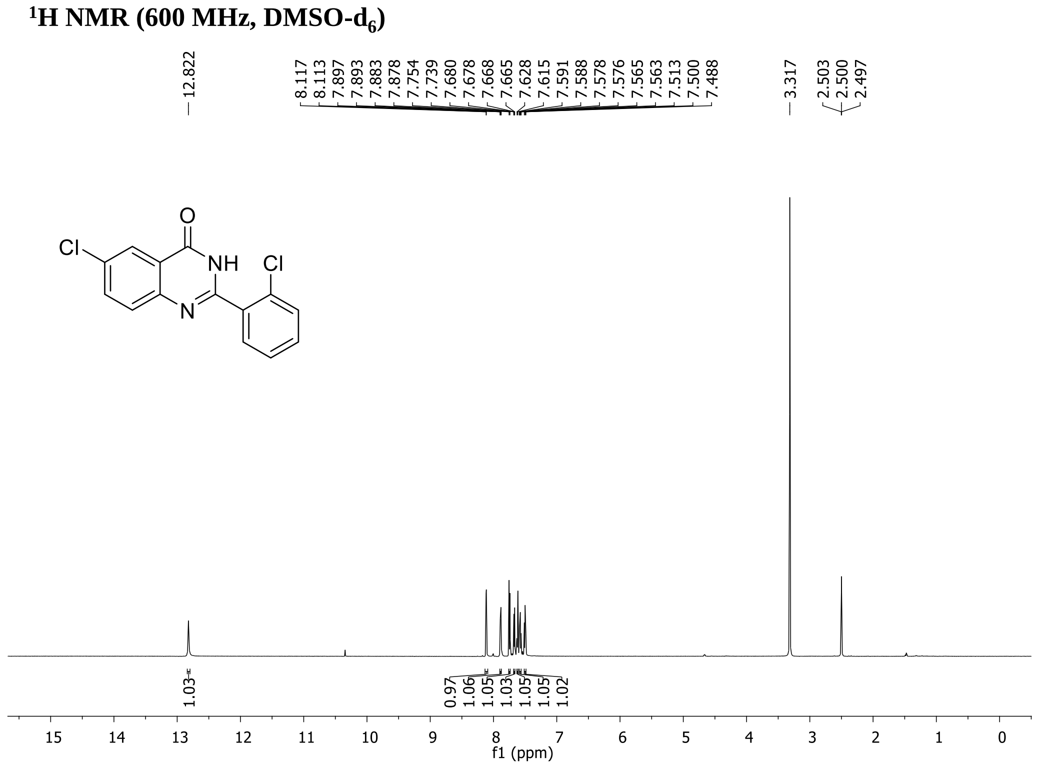

Figure S49. ^{1}H NMR spectrum of 6-Chloro-2-(2-chlorophenyl)quinazolin-4(3*H*)-one (**3cg)**

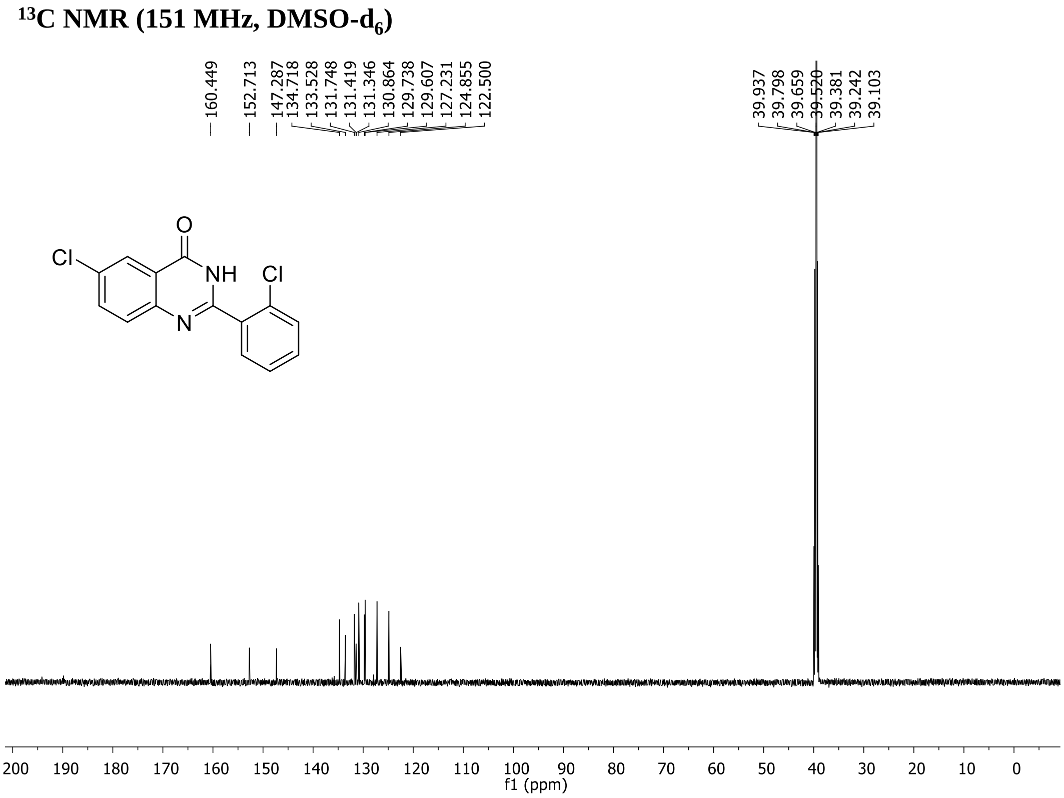

Figure S50. ^{13}C NMR spectrum of 6-Chloro-2-(2-chlorophenyl)quinazolin-4(3*H*)-one (**3cg)**

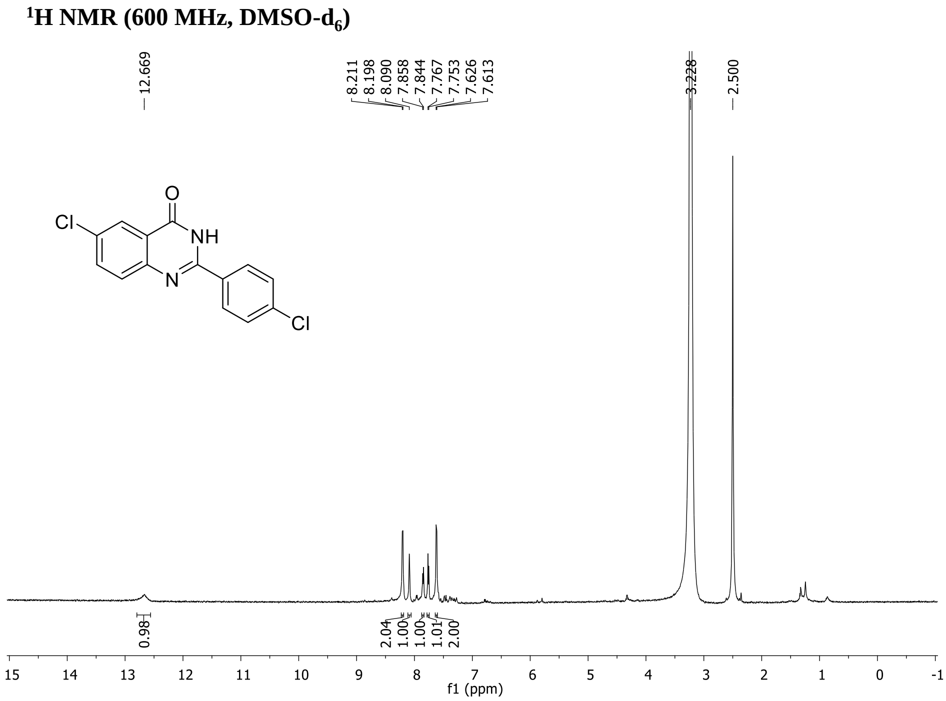

Figure S51. ^{1}H NMR spectrum of 6-Chloro-2-(4-chlorophenyl)quinazolin-4(3*H*)-one (**3ch)**

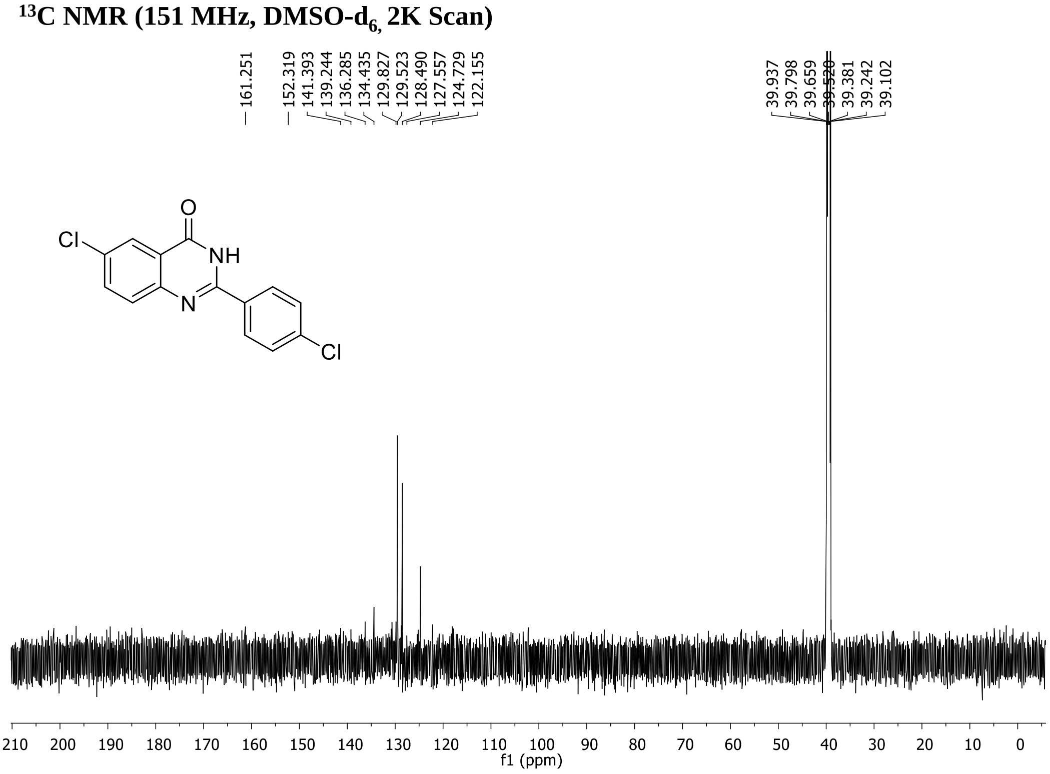

Figure S52. ^{13}C NMR spectrum of 6-Chloro-2-(4-chlorophenyl)quinazolin-4(3*H*)-one (**3ch)**

^{1}H NMR (600 MHz, DMSO-d$_6$)

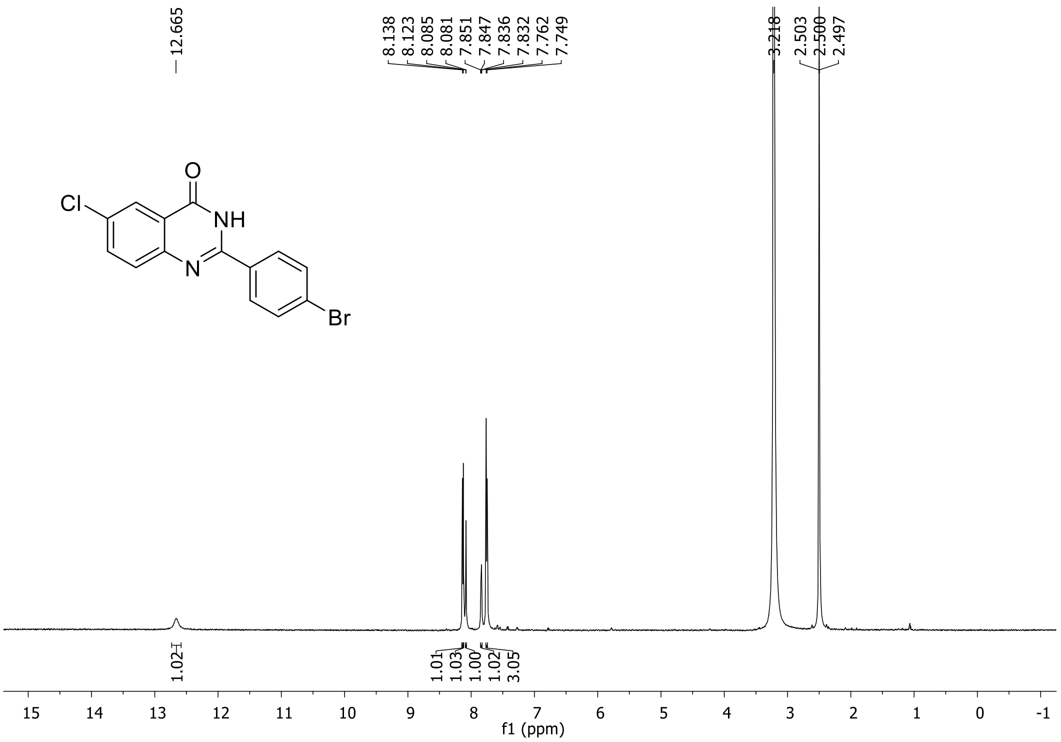

Figure S53. ^{1}H NMR spectrum of 2-(4-Bromophenyl)-6-chloroquinazolin-4(3*H*)-one (**3ci)**

^{13}C NMR (151 MHz, DMSO-d$_6$, 2K Scan)

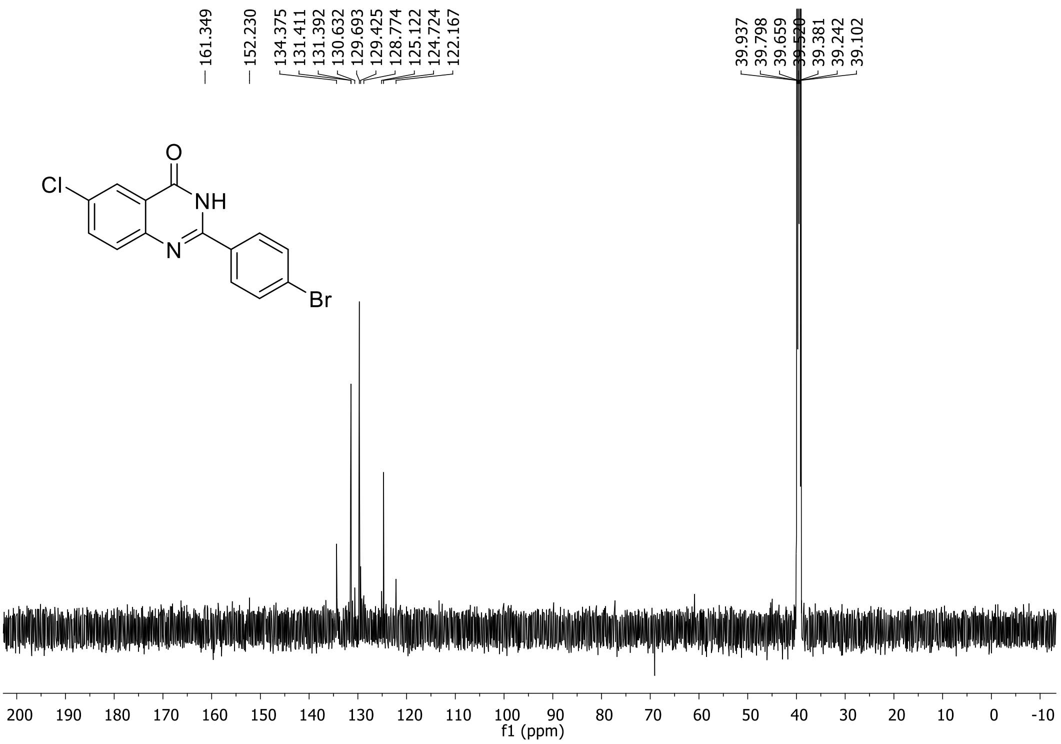

Figure S54. ^{13}C NMR spectrum of 2-(4-Bromophenyl)-6-chloroquinazolin-4(3*H*)-one (**3ci)**

^{1}H NMR (600 MHz, DMSO-d$_6$)

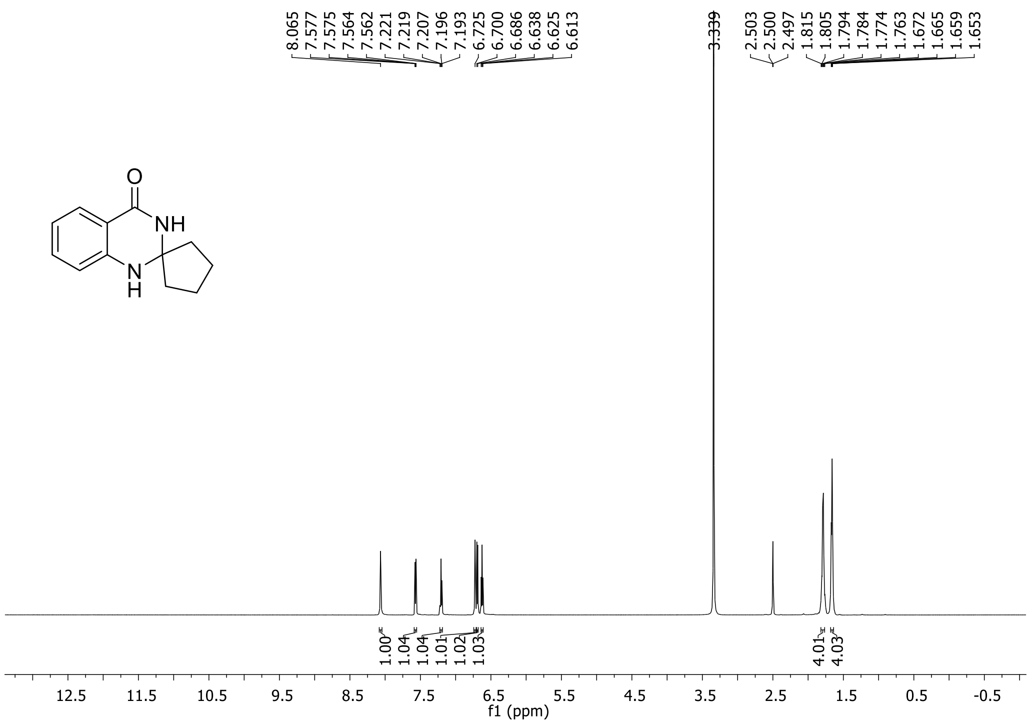

Figure S55. ^{1}H NMR spectrum of 1'*H*-spiro[cyclopentane-1,2'-quinazolin]-4'(3'*H*)-one (**3an**)

^{13}C NMR (151 MHz, DMSO-d$_6$)

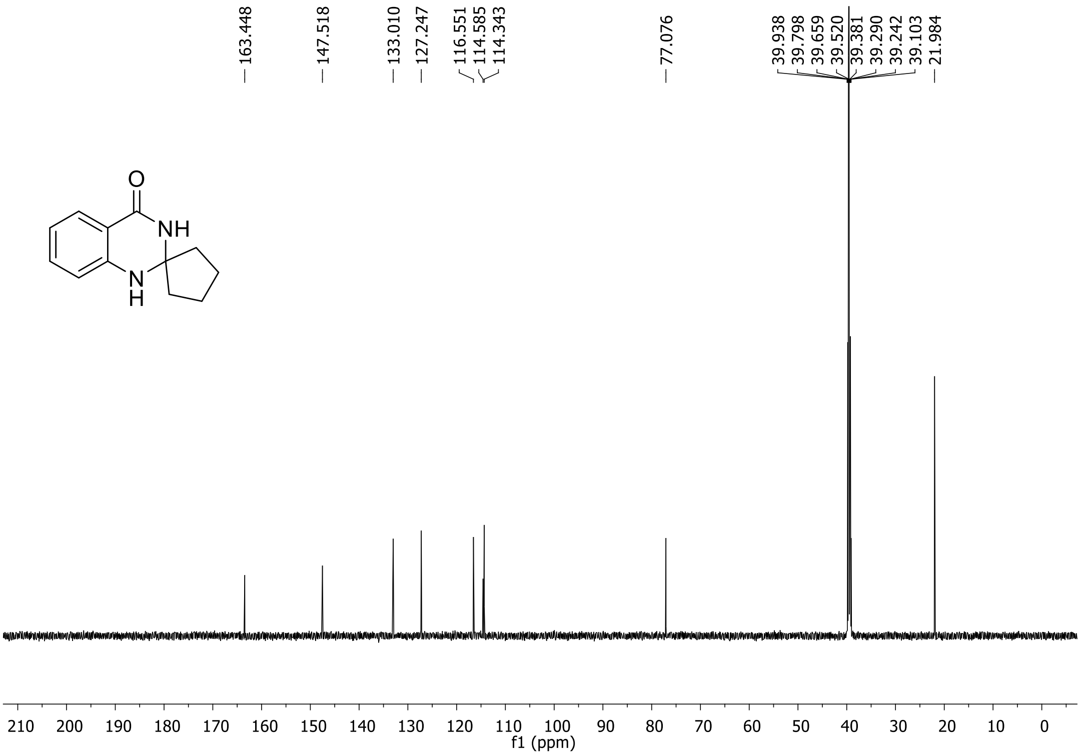

Figure S56. ^{13}C NMR spectrum of 1'*H*-spiro[cyclopentane-1,2'-quinazolin]-4'(3'*H*)-one (**3an**)

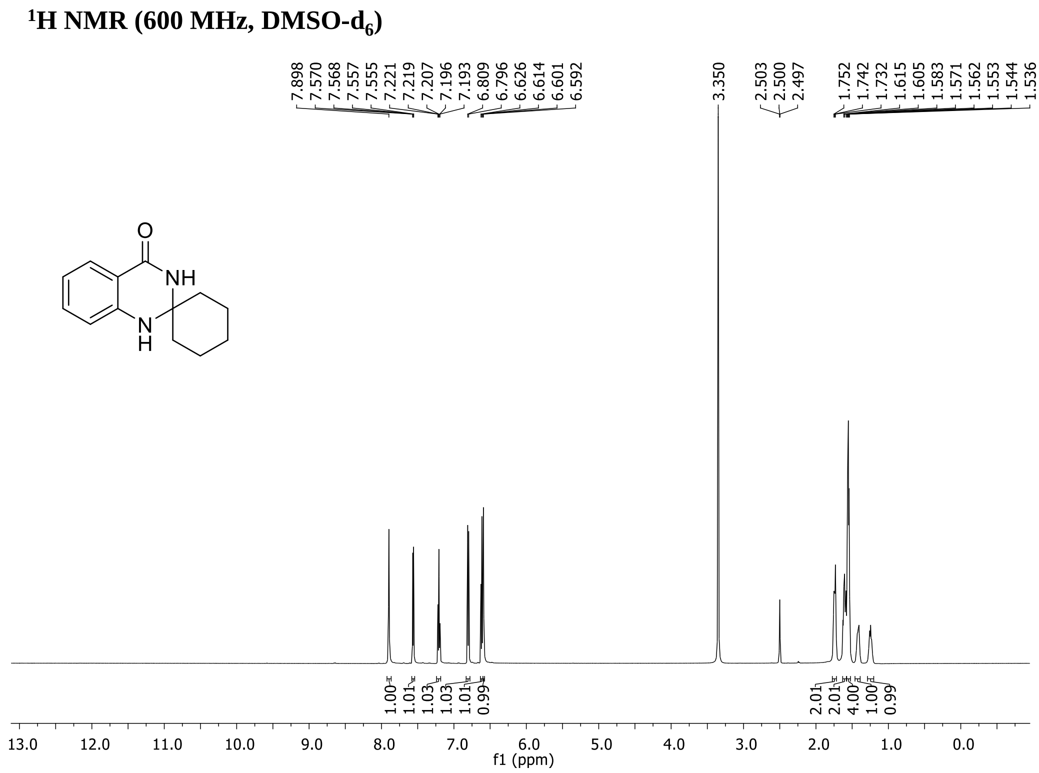

Figure S57. ^{1}H NMR spectrum of 1'*H*-spiro[cyclohexane-1,2'-quinazolin]-4'(3'*H*)-one (**3ao**)

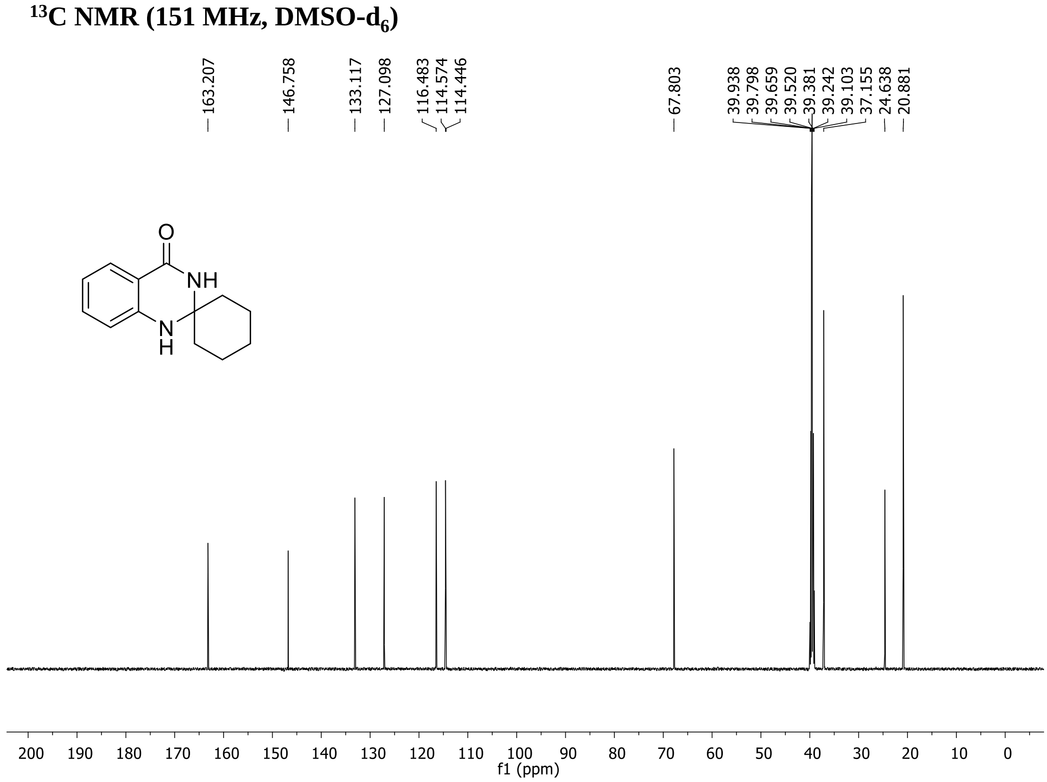

Figure S58. ^{13}C NMR spectrum of 1'*H*-spiro[cyclohexane-1,2'-quinazolin]-4'(3'*H*)-one (**3ao**)

^{1}H NMR (600 MHz, DMSO-d$_6$)

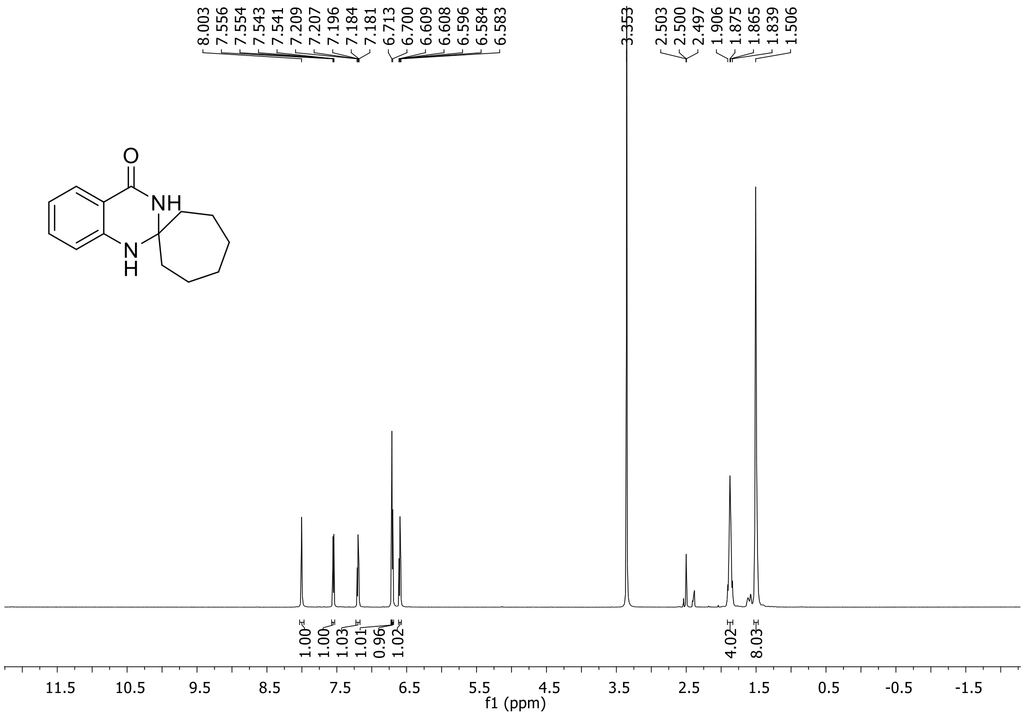

Figure S59. ^{1}H NMR spectrum of 1'*H*-spiro[cycloheptane-1,2'-quinazolin]-4'(3'*H*)-one **(3ap)**

^{13}C NMR (151 MHz, DMSO-d$_6$)

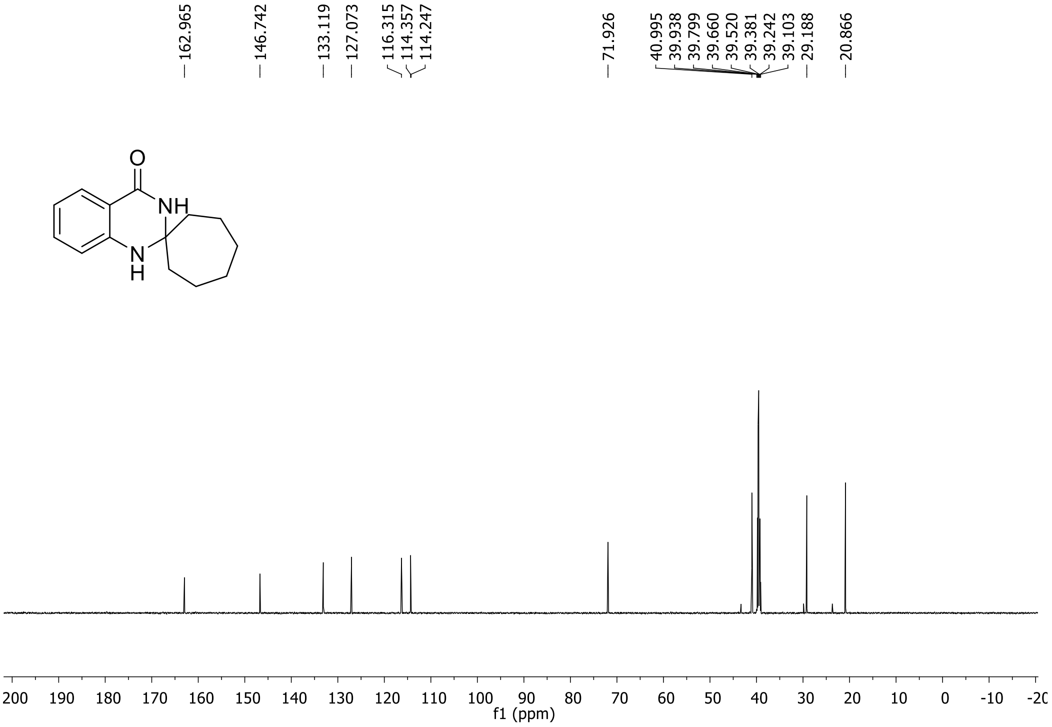

Figure S60. ^{13}C NMR spectrum of 1'*H*-spiro[cycloheptane-1,2'-quinazolin]-4'(3'*H*)-one **(3ap)**

^{1}H NMR (600 MHz, DMSO-d$_6$)

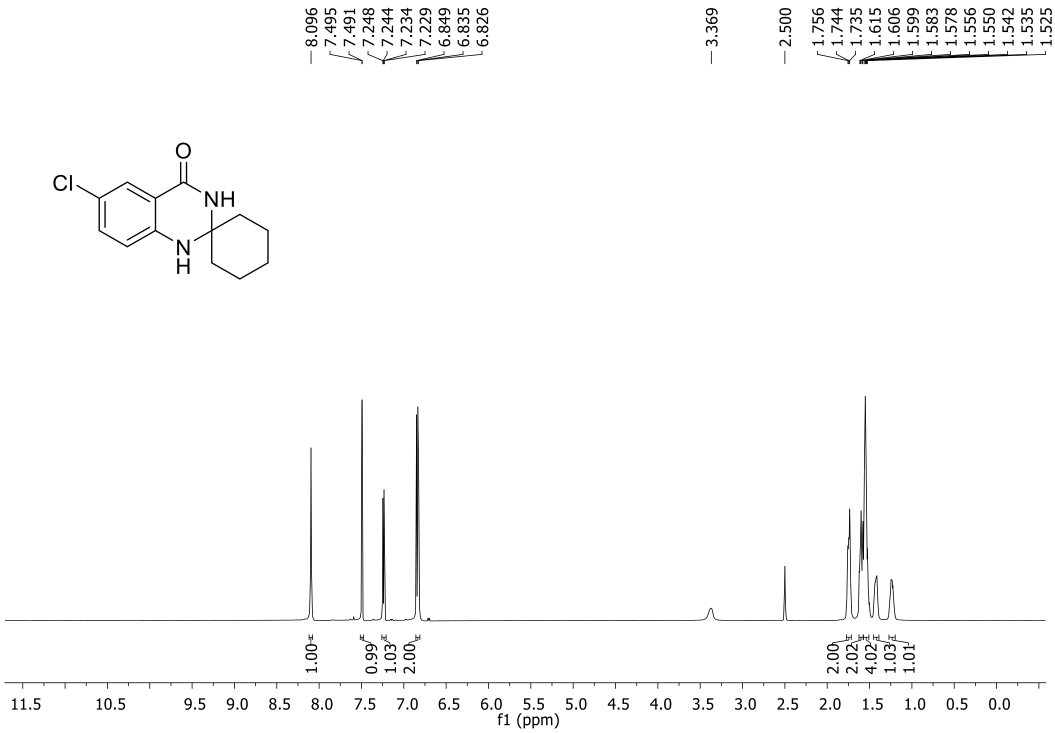

Figure S61. ^{1}H NMR spectrum of 6'-Chloro-1'*H*-spiro[cyclohexane-1,2'-quinazolin]-4'(3'*H*)-one (**3co**)

^{13}C NMR (151 MHz, DMSO-d$_6$)

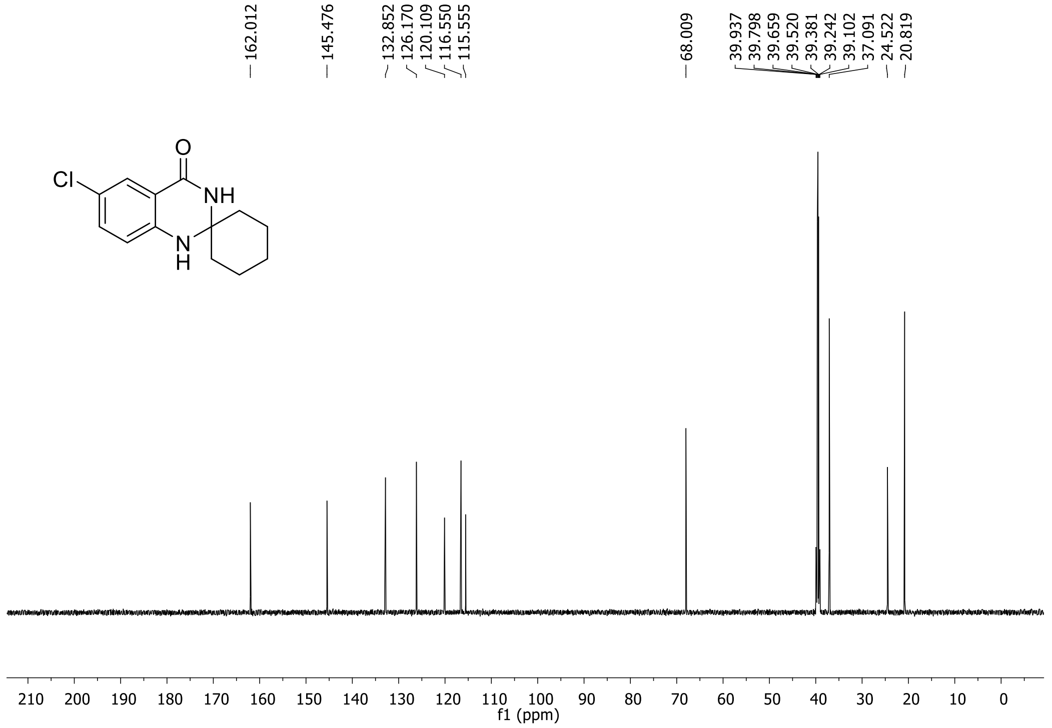

Figure S62. ^{13}C NMR spectrum of 6'-Chloro-1'*H*-spiro[cyclohexane-1,2'-quinazolin]-4'(3'*H*)-one (**3co**)

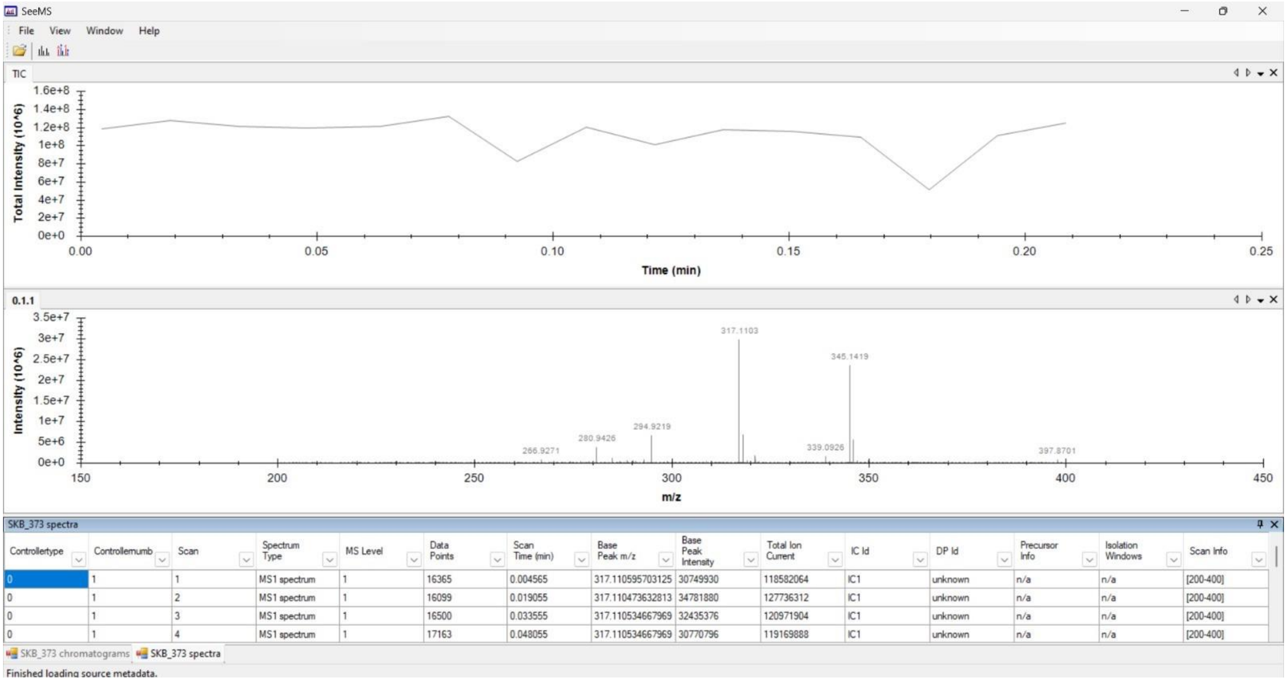

HRMS data for **3bj**: calculated for $C_{20}H_{14}FN_2O$: 317.1090 [M+H]$^+$; found: 317.1103.

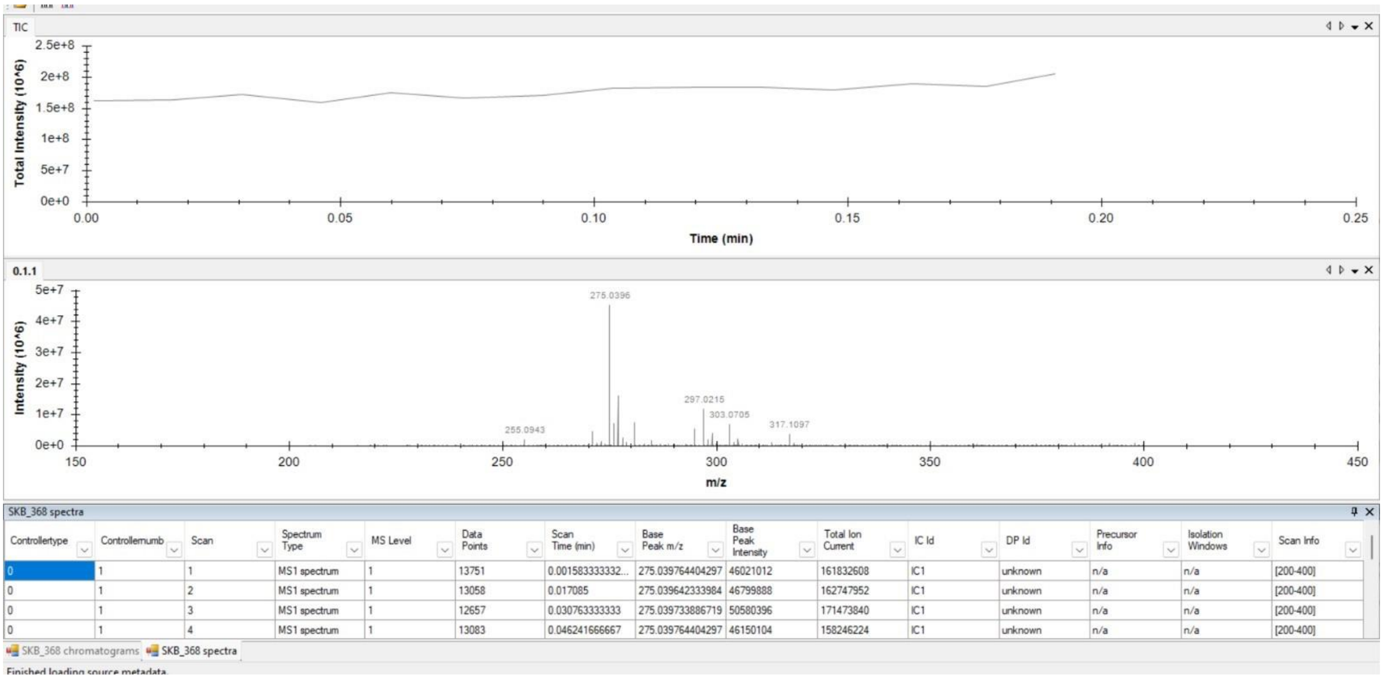

HRMS data for **3bg**: calculated for $C_{14}H_9ClFN_2O$: 275.0387 [M+H]$^+$; found: 275.0396.

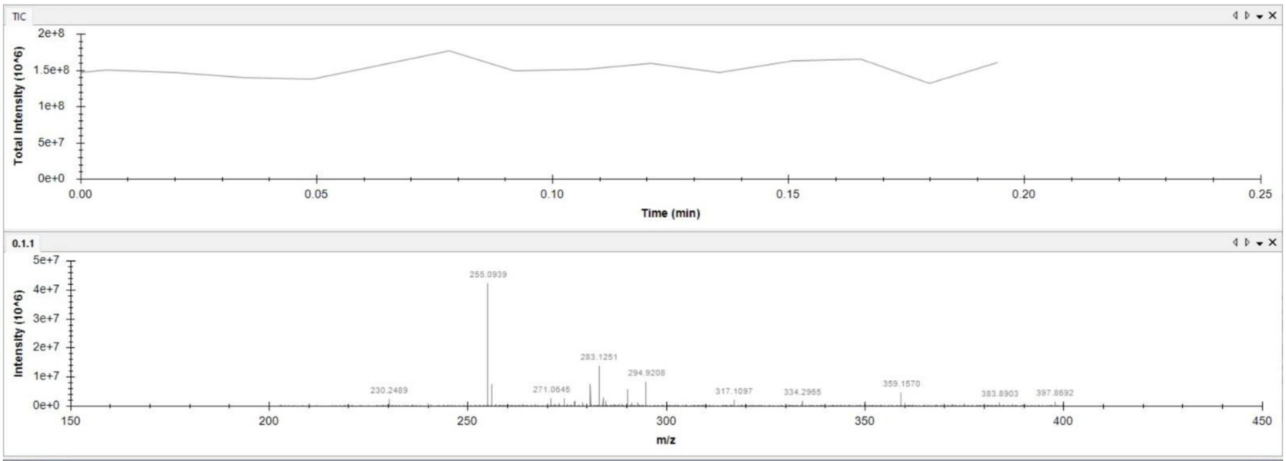

SKB_371 spectra

Controllertype	Controllernumb	Scan	Spectrum Type	MS Level	Data Points	Scan Time (min)	Base Peak m/z	Base Peak Intensity	Total Ion Current	IC Id	DP Id	Precursor Info	Isolation Windows	Scan Info
0	1	1	MS1 spectrum	1	14556	0	255.093994140625	42994028	146526736	IC1	unknown	n/a	n/a	[200-400]
0	1	2	MS1 spectrum	1	14127	0.005551666667	255.093994140625	44289932	149778576	IC1	unknown	n/a	n/a	[200-400]
0	1	3	MS1 spectrum	1	13408	0.020053333333	255.094039916992	43399924	147128400	IC1	unknown	n/a	n/a	[200-400]
0	1	4	MS1 spectrum	1	13831	0.034563333333	255.09407043457	42029788	139972976	IC1	unknown	n/a	n/a	[200-400]

SKB_371 chromatograms SKB_371 spectra

HRMS data for **3be**: calculated for $C_{15}H_{12}FN_2O$: 255.0928 $[M+H]^+$; found: 255.0939.

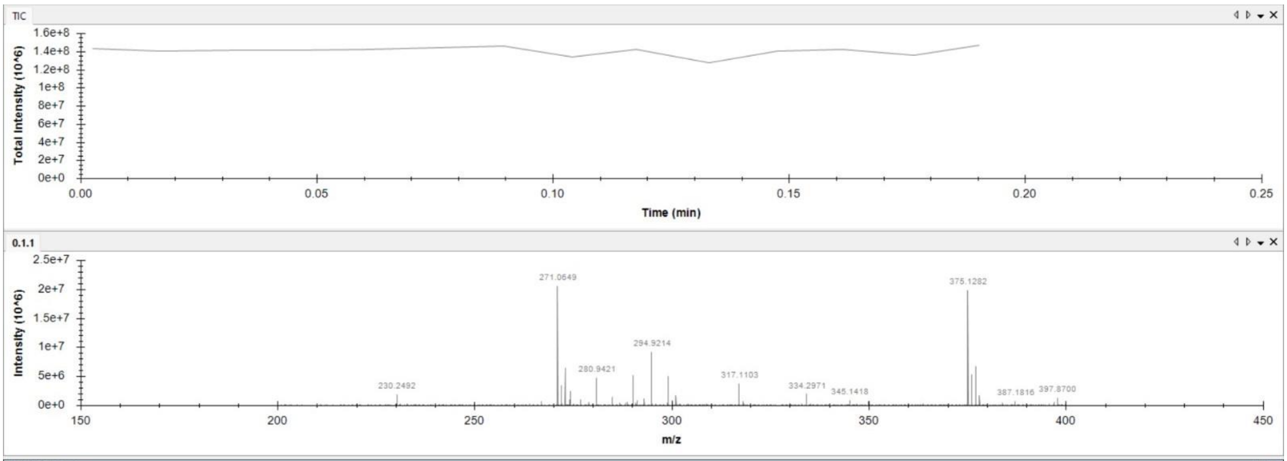

SKB_386 spectra

Controllertype	Controllernumb	Scan	Spectrum Type	MS Level	Data Points	Scan Time (min)	Base Peak m/z	Base Peak Intensity	Total Ion Current	IC Id	DP Id	Precursor Info	Isolation Windows	Scan Info
0	1	1	MS1 spectrum	1	14061	0.002576666667	271.065032958984	20645774	143097456	IC1	unknown	n/a	n/a	[200-400]
0	1	2	MS1 spectrum	1	15037	0.017075	375.127990722656	20521748	140082640	IC1	unknown	n/a	n/a	[200-400]
0	1	3	MS1 spectrum	1	14374	0.03158	375.127777099609	20751224	141437040	IC1	unknown	n/a	n/a	[200-400]
0	1	4	MS1 spectrum	1	14763	0.045241666667	375.128051757813	20808296	141582992	IC1	unknown	n/a	n/a	[200-400]

SKB_386 chromatograms SKB_386 spectra

HRMS data for **3ce**: calculated for $C_{15}H_{12}ClN_2O$: 271.0638 $[M+H]^+$; found: 271.0649.

References

1. Bera, S.K.; Bhanja, R.; Mal, P., DDQ in mechanochemical C–N coupling reactions. *Beilstein J. Org. Chem.* **2022,** *18*, 639–646.
2. Li, Z.; Dong, J.; Chen, X.; Li, Q.; Zhou, Y.; Yin, S.-F., Metal- and Oxidant-Free Synthesis of Quinazolinones from β-Ketoesters with o-Aminobenzamides via Phosphorous Acid-Catalyzed Cyclocondensation and Selective C–C Bond Cleavage. *J. Org. Chem.* **2015,** *80*, 9392–9400.
3. Sardar, B.; Jamatia, R.; Samanta, A.; Srimani, D., Ru Doped Hydrotalcite Catalyzed Borrowing Hydrogen-Mediated *N*-Alkylation of Benzamides, Sulfonamides, and Dehydrogenative Synthesis of Quinazolinones. *J. Org. Chem.* **2022,** *87*, 5556–5567.
4. Alam, M.T.; Maiti, S.; Mal, P., The mechanochemical synthesis of quinazolin-4(3*H*)-ones by controlling the reactivity of IBX. *Beilstein J. Org. Chem.* **2018,** *14*, 2396–2403.
5. Huang, S.; Jin, L.; Liu, Y.; Yang, G.; Wang, A.; Le, Z.; Jiang, G.; Xie, Z., Visible light-mediated synthesis of quinazolinones from benzyl bromides and 2-aminobenzamides without using any photocatalyst or additive. *Org. Biomol. Chem.* **2024,** *22*, 784–789.
6. Huang, J.; Chen, W.; Liang, J.; Yang, Q.; Fan, Y.; Chen, M.-W.; Peng, Y., α-Keto Acids as Triggers and Partners for the Synthesis of Quinazolinones, Quinoxalinones, Benzooxazinones, and Benzothiazoles in Water. *J. Org. Chem.* **2021,** *86*, 14866–14882.
7. Dutta, B.; Dutta, N.; Dutta, A.; Gogoi, M.; Mehra, S.; Kumar, A.; Deori, K.; Sarma, D., [DDQM][HSO(4)]/TBHP as a Multifunctional Catalyst for the Metal Free Tandem Oxidative Synthesis of 2-Phenylquinazolin-4(3*H*)-ones. *J. Org. Chem.* **2023,** *88*, 14748–14752.
8. Yang, X.; Cheng, G.; Shen, J.; Kuai, C.; Cui, X., Cleavage of the C–C triple bond of ketoalkynes: synthesis of 4(3*H*)-quinazolinones. *Org. Chem. Front.* **2015,** *2*, 366–368.
9. Zhang, Z.; Wang, M.; Zhang, C.; Zhang, Z.; Lu, J.; Wang, F., The cascade synthesis of quinazolinones and quinazolines using an α-MnO_2 catalyst and tert-butyl hydroperoxide (TBHP) as an oxidant. *Chem. Commun.* **2015,** *51*, 9205–9207.
10. Mahmoud, M.R.; El-Bordany, E.A.A.; Hassan, N.F.; El-Azm, F.S.M.A., New 2,3-Disubstituted Quinazolin-4(3*H*)-Ones from 2-Undecyl-3,1-Benzoxazin-4-One. *Journal of Chemical Research* **2007,** *2007*, 541–544.
11. Feng, Y.; Li, Y.; Cheng, G.; Wang, L.; Cui, X., Copper-Catalyzed Synthesis of 2-Arylquinazolinones from 2-Arylindoles with Amines or Ammoniums. *J. Org. Chem.* **2015,** *80*, 7099–7107.
12. Liu, Z.; Zeng, L.Y.; Li, C.; Yang, F.; Qiu, F.; Liu, S.; Xi, B., "On-Water" Synthesis of Quinazolinones and Dihydroquinazolinones Starting from o-Bromobenzonitrile. *Molecules* **2018,** *23*, 2325.
13. Xu, G.; Wang, L.; Li, M.; Tao, M.; Zhang, W., Phosphorous acid functionalized polyacrylonitrile fibers with a polarity tunable surface micro-environment for one-pot C–C and C–N bond formation reactions. *Green Chem.* **2017,** *19*, 5818–5830.
14. Shang, Y.-H.; Fan, L.-Y.; Li, X.-X.; Liu, M.-X., $Y(OTf)_3$-catalyzed heterocyclic formation via aerobic oxygenation: An approach to dihydro quinazolinones and quinazolinones. *Chin. Chem. Lett.* **2015,** *26*, 1355–1358.
15. Li, H.; He, L.; Neumann, H.; Beller, M.; Wu, X.-F., Cascade synthesis of quinazolinones from 2-aminobenzonitriles and aryl bromides via palladium-catalyzed carbonylation reaction. *Green Chem.* **2014,** *16*, 1336–1343.
16. Patel, S.M.; Chada, H.; Biswal, S.; Sharma, S.; Sharada, D.S., Copper-Catalyzed Intramolecular α-C–H Amination via Ring-Opening Cyclization Strategy to Quinazolin-4-ones: Development and Application in Rutaecarpine Synthesis. *Synthesis* **2019,** *51*, 3160–3170.
17. Majumdar, B.; Sarma, D.; Jain, S.; Sarma, T.K., One-Pot Magnetic Iron Oxide–Carbon Nanodot Composite-Catalyzed Cyclooxidative Aqueous Tandem Synthesis of Quinazolinones in the Presence of tert-Butyl Hydroperoxide. *ACS Omega* **2018,** *3*, 13711–13719.
18. Gnyawali, K.; Kirinde Arachchige, P.T.; Yi, C.S., Synthesis of Flavanone and Quinazolinone Derivatives from the Ruthenium-Catalyzed Deaminative Coupling Reaction of 2′-Hydroxyaryl Ketones and 2-Aminobenzamides with Simple Amines. *Org. Lett.* **2022,** *24*, 218–222.

Disclaimer/Publisher's Note: The statements, opinions and data contained in all publications are solely those of the individual author(s) and contributor(s) and not of MDPI and/or the editor(s). MDPI and/or the editor(s) disclaim responsibility for any injury to people or property resulting from any ideas, methods, instructions or products referred to in the content.
